# A Novel Bis-Spiroketal Scaffold and Other Secondary Metabolites from the Marine-Derived Fungus *Talaromyces stipitatus* HF05001: Structural Diversity and Bioactivities

**DOI:** 10.3390/md24010047

**Published:** 2026-01-19

**Authors:** Longhe Yang, Yan Qiu, Ying Liu, Xiaoyu Wei, Xiwen He, Yiling Wang, Yajun Yan, Kaikai Bai, Zhaokai Wang, Jie Ren

**Affiliations:** 1 Technical Innovation Center for Utilization of Marine Biological Resources, Third Institute of Oceanography, Ministry of Natural Resources, Xiamen 361000, China; 2 School of Medicine, Xiamen University, Xiamen 361102, China; 3 Xiamen Key Laboratory of Chiral Drugs, Xiamen 361102, China

**Keywords:** secondary metabolites, marine-derived fungus, anti-Inflammation, anti-adipogenic activities

## Abstract

Marine-derived fungi have become a vital resource for the discovery of novel secondary metabolites with diverse structures and significant biological activities. This study focuses on a systematic chemical investigation of the sponge-associated fungus *Talaromyces stipitatus* HF05001, leading to the isolation and identification of 20 compounds, including one new marine ketal natural product (Compound **17**, Talarobispiral A). These compounds were structurally elucidated using comprehensive spectroscopic analyses, including 1D and 2D NMR, HRESIMS. All isolates were screened for their anti-inflammatory and anti-adipogenic properties. Among them, compound **4** (Secalonic acid D, SAD), **7** (Sch 725680) and **16** (bacillisporins C) demonstrated significant anti-inflammatory potential by markedly suppressing nitric oxide (NO) production in lipopolysaccharide (LPS)-stimulated RAW264.7 macrophages. Notably, compound **4** showed superior inhibitory effect, with an IC_50_ value of 0.22 μM. Additionally, compound **4** exhibited the strongest dose-dependent inhibition of lipid droplet accumulation in 3T3-L1 preadipocytes. These findings highlight the dual therapeutic potential of metabolites from *Talaromyces stipitatus*, identifying promising lead compounds for the development of novel treatments for inflammatory and metabolic disorders.

## 1. Introduction

Marine ecosystems, especially those with extreme conditions like high pressure, low oxygen, and temperature fluctuations, are home to an extraordinary diversity of microorganisms [1]. These unique environmental pressures have driven the evolution of marine microbes to develop intricate metabolic pathways, enabling them to produce a wide array of secondary metabolites with novel structures and potent bioactivities [2,3,4]. Among these, marine-derived fungi have emerged as a rich source of bioactive secondary metabolites, including polyketides [5], terpenoids [6], alkaloids [7], and steroids [8]. These metabolites often exhibit significant biological activities, such as antimicrobial, antiviral, anticancer, and anti-inflammatory effects, thus positioning them as promising candidates for drug discovery [3,9].

*Talaromyces stipitatus* is a marine-derived fungus that has garnered attention for its potential to produce bioactive secondary metabolites. Previous studies on this fungus have led to the isolation of various compounds, including ergosterol derivatives, anthraquinones [10], phenalenones and polyesters [11,12], steroids [13] which have demonstrated activities such as antibacterial, antiviral, antioxidant, and anti-inflammatory effects. For example, Questinol and citreorosein showed significant anti-obesity activity in zebrafish models, while some anthraquinones were toxic at tested doses [10]. emodin, a representative anthraquinone compound, has been reported to exhibit immunomodulatory, antibacterial, and anti-inflammatory activities [14]. Other compounds like talaromycin and secalonic acid D (SAD) have also shown significant biological activities [11,15]. Notably, spiroketal scaffolds, such as those in bis(oxaphenalenone) systems, are exceptionally rare in marine natural products, with only a handful of terrestrial analogs reported, underscoring the potential novelty of such structures in oceanic ecosystems [16]. These findings suggest that *Talaromyces stipitatus* is a promising source of novel bioactive compounds with potential therapeutic applications.

Chronic, low-grade inflammation is now recognized as a key pathological driver in the development and progression of obesity and related metabolic disorders. This intricate link, often termed "meta-inflammation", suggests that agents possessing dual anti-inflammatory and anti-adipogenic properties could offer superior therapeutic advantages [17]. Therefore, the discovery of natural products that can modulate both pathways is of significant scientific interest.

In our continuous efforts to discover novel bioactive compounds from marine microorganisms, we conducted a systematic investigation of the secondary metabolites from *Talaromyces stipitatus* HF05001. This study aimed not only to uncover the chemical diversity of this fungus but also to specifically evaluate its potential to yield compounds targeting the interconnected pathways of inflammation and adipogenesis, offering new avenues for therapeutic intervention.

## 2. Results and Discussion

**Figure 1:**
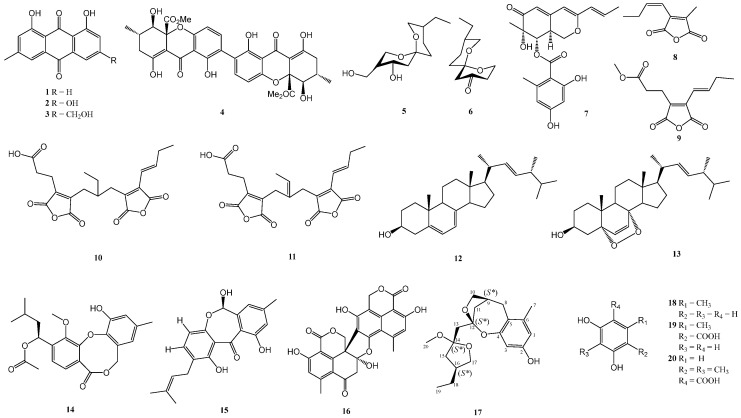
Chemical structures of the isolated compounds **1**–**20** from *Talaromyces stipitatus* HF05001.

The EtOAc extract of Talaromyces stipitatus HF05001 was subjected to a series of chromatographic techniques, including silica gel column chromatography, ODS column chromatography, Sephadex LH-20 gel permeation chromatography, and semi-preparative HPLC, yielding a total of 20 compounds (**1**–**20,** Figure 1). The structures of these compounds were elucidated using comprehensive analyses of 1D and 2D NMR, HRESIMS. 

Compound **17** was obtained as a pale-yellow oil. HRESIMS data showed a protonated molecule peak [M+H]^+^ at *m*/*z* 349.20135 (Figure S7), consistent with a molecular weight of 348 and molecular formula of C_20_H_28_O_5_ (calcd. 348.1937). Further evidence for the molecular formula was provided by the sodium adduct [M+Na]^+^ at *m*/*z* 371.18317 and the dimeric sodium adduct [2M+Na]^+^ at *m*/*z* 719.37726.

**Figure 2:**
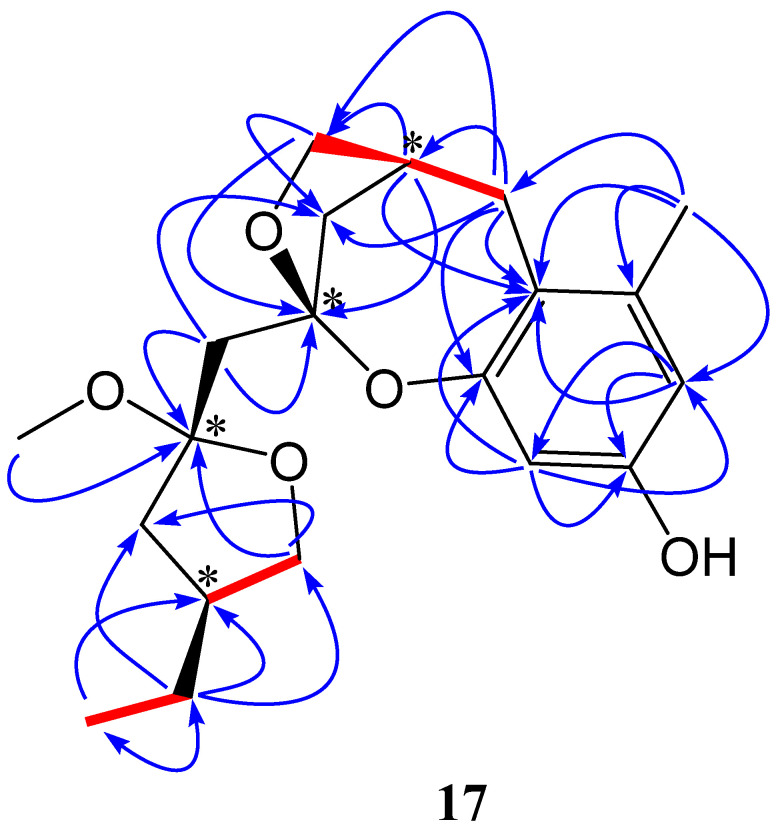
The key HMBC (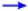) and COSY (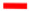) correlations of compound **17**.

The ^13^C-NMR data for compound **17** revealed 20 carbon atoms (Table 1, Figure S2). DEPT spectra further classified these as three methyls, seven methylenes, four methines, and six quaternary carbons (Figure S3). The ^1^H-NMR spectrum showed two characteristic meta-coupled proton signals in the aromatic region: δ_H_ 6.34 (1H, d, J = 2.4 Hz, H-1) and δ_H_ 6.44 (1H, d, J = 2.4 Hz, H-3) (Figure S1). This suggested the presence of a 2,4,5,6-tetrasubstituted benzene ring. HMBC correlations further supported this assignment (Figure S6). The proton signal at δ_H_ 6.34 showed correlations with carbons at δ_C_ 19.1, 101.8, and 111.3. The proton at δ_H_ 6.44 correlated with carbons at δ_C_ 110.1, 111.3, 151.8, and 154.3. These correlations definitively established the substitution pattern on the benzene ring (Figure 2). The methyl signal at δ_H_ 2.17 (s), corresponding to δ_C_ 19.1, exhibited HMBC correlations with carbons at δ_C_ 110.1, 111.3, and 138.2. This further corroborated the proposed structure shown in Figure 2. The downfield chemical shifts in C-2 (δ_C_ 154.3) and C-4 (δ_C_ 151.8) indicated oxygen substituents at these positions.

The ^1^H-NMR spectrum displayed two downfield doublet of doublets signals (Figure S1). The first set included δ_H_ 3.56 (1H, dd, *J* = 11.2, 4.5 Hz) and 3.24 (1H, dd, *J* = 11.2, 11.0 Hz) and the other one comprised δ_H_ 3.45 (1H, dd, *J* = 11.7, 4.7 Hz) and 3.68 (1H, dd, *J* = 11.8, 11.7 Hz). HSQC correlations assigned these proton signals to carbons at δ_C_ 65.5 and 62.0, respectively (Figure S5). This indicated the presence of two -CH_2_O- moieties directly attached to -CH groups. Further analysis of HMBC correlations confirmed a furan ring (Figure 2). Specifically, δ_H_ 3.68 and 3.45 (H-10) showed correlations with δ_C_ 96.6 (C-12). COSY correlations between δ_H_ 3.68, 3.45 (H-10) and δ_H_ 2.29 (H-9), and between δ_H_ 2.92, 2.19 (H-8) and H-9, further supported the furan ring’s presence. The unusually downfield chemical shift in C-12 (δ_C_ 96.6) suggested that this quaternary carbon was disubstituted by two oxygen atoms (Figure 2). DEPT (Figure S3) and HSQC (Figure S5) spectra revealed a characteristic methylene (-CH_2_-) signal at δ_H_ 2.92 (1H, dd, *J* = 16.8, 7.8 Hz) and 2.19 (1H, dd, *J* = 16.8, 2.4 Hz), corresponding to δ_C_ 20.4 (C-8). This methylene showed HMBC correlations with carbons at δ_C_ 36.2, 62.0, 111.3, 138.2, and 151.8. These connections unequivocally linked the furan ring to the previously established benzene ring.

HMBC correlations between δ_H_ 3.56, 3.24, and δ_C_ 96.7 suggested the presence of another furan ring (Figure 2). The exceptionally downfield shift in C-12 (δ_C_ 96.7) indicated another quaternary carbon disubstituted by two oxygen atoms. DEPT (Figure S3) and HSQC (Figure S5) spectra identified a methyl signal at δ_H_ 0.88 (3H, t, *J* = 7.5 Hz), corresponding to C-19 (δ_C_ 11.1). COSY correlations linked δ_H_ 0.88 to δ_H_ 1.16 (2H, m, H-18), confirming the adjacency of C-19 and C-18. HMBC correlations from δ_H_ 0.88 to δ_C_ 25.2 and 36.3 further connected this furan ring to the -CH_2_-CH_3_ fragment (Figure 2). Two proton signals at δ_H_ 2.48 (1H, d, *J* = 13.8 Hz) and 1.67 (1H, d, *J* = 13.8 Hz) showed a characteristic geminal coupling constant of 13.8 Hz, indicating a -CH_2_- group flanked by two quaternary carbons. Both methylene protons correlated with δ_C_ 96.6 and 96.7 in the HMBC spectrum. This linkage connected fragments 2B and 2C via this methylene group. These findings elucidated the planar structure of compound **17**, revealing it as a rare marine ketal natural product (Figure 2).

Regarding the stereochemistry of **17**, compound **17** possesses four stereogenic centers (C-9, C-12, C-14, and C-16). Among them, C-9 and C-12 are connected through a bridged five-membered ring system, which imposes structural rigidity requiring a fixed syn relationship between these two centers. Unfortunately, the NOESY data obtained for compound **17** were insufficient to establish the relative configuration. It should be made clear that, due to this limitation, an NMR calculation-based analysis was necessarily employed to support the relative configuration assignment. Consequently, although 2^4^ = 16 absolute configurations are theoretically possible, this constraint reduces the number of feasible relative configurations to four (2^3^ = 8 absolute configurations corresponding to four pairs of enantiomers). There are thus four possible relative configurations among those chiral centers in total, namely 9S*,12S*,14S*,16S*-**17**, 9S*,12S*,14S*,16R*-**17**, 9S*,12S*,14R*,16S*-**17** and 9S*,12S*,14R*,16R*-**17**. 

It is important to note that NMR chemical shift calculations cannot distinguish between enantiomers. Consequently, only one representative enantiomeric series, namely the (9S*,12S*) series, was used in the NMR calculations to determine the relative configuration. A GIAO-based NMR calculation was then employed to predict the chemical shifts in the four potential diastereoisomers. A subsequent DP4+ probability analysis revealed a clear inclination (99.35%) for the first diastereoisomer (9S*,12S*,14S*,16S*-**17**) (Supplementary Materials Table S1). Thus, the relative configuration of compound **17** was established as 9S*,12S*,14S*,16S* as shown in Figure 2. Only one enantiomeric series was computed due to the indistinguishability of enantiomers by NMR. It should be emphasized that the DP4+ analysis was used solely to assign the relative configuration; no claim is made regarding the absolute configuration, which could not be determined experimentally or computationally due to material limitations and the absence of a strong ECD chromophore. 

To the best of our knowledge, compound **17** is the first member of this skeleton isolated from a marine-derived *Talaromyces* strain. Thus, compound **17** represents an unprecedented marine fungal natural product featuring a unique bis-spiroketal architecture, and has been designated Talarobispiral A.

By comparison of the NMR and MS data with those published in the literature, 19 known compounds were identified as emodin (**1**) [18], chrysophanic acid (**2**) [19], physcion (**3**) [20], secalonic acid D (**4**) [21], talaromycin A (**5**) [22], (3R, 4S, 6R,9R)-9-Ethyl-4-hydroxy-3-hydroxymethyl-1,7-dioxaspiro-[5.5] (**6**) [23], Sch 725680 (**7**) [24], 3-(1-buten-1-yl)-4-methyl-2,5-furandione (**8**) [25], 3-furanpropanoic acid, 4-(1-butenyl)-2,5-dihydro-2,5-dioxo-, methyl ester (**9**) [26], cordyanhydride A (**10**) [27], methyate cordyanhydride A (**11**) [28], ergosterol (**12**) [29], ergosterol peroxide (**13**) [30], paeciloxocins B (**14**) [31], 1,6,10-trihydroxy-8-methyl-2-(3-methyl-2-butenyl)-dibenz[b,e]oxepin-11(6H)-one (**15**) [32], bacillisporins C (**16**) [33], 5-methyl-1,3-benzenediol (**18**) [34], orsellinic acid (**19**) [35], and 1-(2,4-dihydroxy-3,5-dimethylphenyl)-ethanone (**20**) [36]. 

The anti-inflammatory activities of compounds **1**–**20** were evaluated using the Griess method to measure nitric oxide (NO) production in LPS-stimulated RAW264.7 macrophage cells [37,38]. Prior to anti-inflammatory screening, compounds 1–20 were evaluated for cytotoxicity against RAW264.7 macrophages using the CCK8 assay, revealing generally non-toxicity except Compound 4 (Figure S74), which supports the specificity of observed NO inhibition effects (Table 2). The results showed that several compounds exhibited significant inhibition of NO production (Table 2). Notably, compound **7** (Sch 725680) demonstrated potent anti-inflammatory activity with an NO inhibition rate of 85.25% ± 4.20% at 10 µM, while compound **16** (Bacillisporin C) also showed remarkable activity with an NO inhibition rate of 81.97% ± 1.76%, comparable to the positive control BAY 11-7085, which exhibited an NO inhibition rate of 85.63% ± 5.48%. However, it is important to note that while compound **4** (Secalonic acid D) exhibited strong NO inhibition (90.04% ± 3.47%), it also showed significant cytotoxicity, with a cell viability inhibition of 98.21% ± 3.35%, indicating potential cell toxicity at the tested concentration (10 μM). The potent cytotoxicity of SAD at 10 μM may stem from apoptotic pathways, warranting caution in therapeutic development at high concentration [39]. Other compounds, including the novel compound **17**, showed some level of inhibitory activity against NO production, with an inhibition rate of 36.10% ± 7.29% at 10 µM. However, this activity was relatively weak compared to the more potent compounds such as compound **7** and compound **16**. Notably, the NO inhibition rate of compound **17** did not exceed 50%, indicating that its anti-inflammatory potential may be limited at the tested concentration.

**Figure 3:**
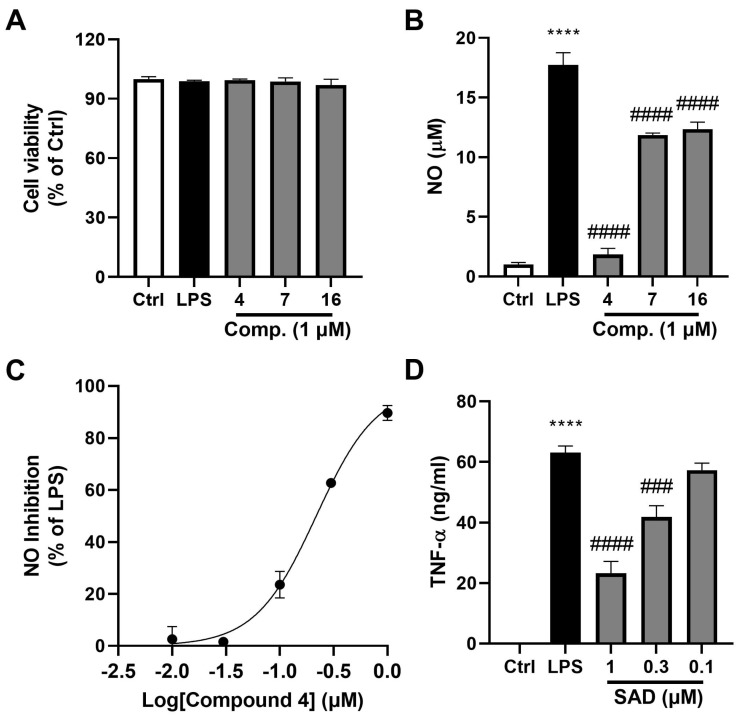
Inhibitory effects of compounds **4**, **7**, and **16** on NO production in LPS-stimulated RAW264.7 macrophage cells. (**A**) Cell viability was assessed using a CCK-8 assay, with the results presented as a percentage of the control group (non-treated cells). (**B**) NO levels (μM) after treatment with 1 μM of compounds **4**, **7**, and **16**. (**C**) Dose–response curve for compound **4** showing NO inhibition percentage relative to LPS-stimulated levels. (**D**) TNF-α levels were quantified in the culture supernatant after treatment with SAD at various concentrations. Data are expressed as the mean ± standard error medium (n = 4). Statistical significance is indicated as follows: **** *p* < 0.0001 versus the control group (Ctrl); #### *p* < 0.0001, ### *p* < 0.001 versus the LPS group.

To further assess the anti-inflammatory activity of compounds **4**, **7**, and **16,** we first confirmed their lack of cytotoxicity by evaluating cell viability in LPS-stimulated cells treated with each compound at 1 μM. As shown in Figure 3A, neither LPS nor the compounds significantly affected cell viability, which remained approximately 100% relative to the untreated control, indicating that the tested concentration was non-toxic and suitable for subsequent anti-inflammatory evaluations. We then examined their inhibitory effects on NO production at this non-toxic concentration of 1 μM. Compound **4** demonstrated significant NO inhibition at 1 μM, with an inhibition rate exceeding 50%, which was notably higher than that of compounds **7** and **16**, indicating its superior anti-inflammatory potential at this concentration (Figure 3B).

Given the promising inhibitory activity observed for compound **4** despite its comparatively modest effect at 1 μM, we proceeded to determine its half-maximal inhibitory concentration (IC_50_) for NO production. The dose–response experiment revealed a concentration-dependent inhibitory effect of compound 4 on NO generation, with increasing inhibition as concentrations rose on a logarithmic scale. The IC_50_ value for compound 4 was determined to be 0.22 μM (Figure 3C), indicating that it effectively suppresses the release of this inflammatory mediator at sub-micromolar concentrations and possesses potent anti-inflammatory potential. We also assessed the impact of compound **4** on TNF-α production in LPS-stimulated cells. Compound **4** treatment resulted in a dose-dependent reduction in TNF-α levels, with higher concentrations yielding greater suppression compared to the LPS group (Figure 3D).

Collectively, these results suggest that compounds **4**, **7**, and **16** may serve as promising lead compounds for the development of novel anti-inflammatory agents, with compounds 7 and 16 showing particularly robust effects on NO inhibition at low doses. Further studies are underway to elucidate the mechanisms of action underlying these effects, with a focus on compound **4**, and to evaluate their in vivo anti-inflammatory activity in relevant animal models. 

Compound **7** (Sch 725680), a novel hydrogenated azaphilone initially isolated from Aspergillus species, was previously reported for antimicrobial and DNA polymerase inhibitory activities but not anti-inflammatory [24,40].This compound exhibits antifungal activity against Saccharomyces cerevisiae (PM503) and Candida albicans (C43), with minimum inhibitory concentrations (MICs) of 8 μg/mL and 64 μg/mL, respectively [24]. Bacillisporin C is produced by the fungus *Talaromyces bacillisporus*, which has been studied for its ability to generate various oxaphenalenone dimers, including bacillisporins A, B, and C, as well as other analogs. It has demonstrated moderate cytotoxic activity against several human cancer cell lines, including MCF-7, NCI-H460, and SF-268. While bacillisporin A showed strong activity, bacillisporin C, along with bacillisporin B and duclauxin, exhibited low cytotoxic effects in these assays (IC_50_) between 26–48 μM [41]. Bacillisporin C for isolation without anti-inflammatory reports [11,41]. Significantly, the present study provides the first evidence that both Compound **7** (Sch 725680) and Compound **16** (Bacillisporin C) exhibit potent inhibitory effects on nitric oxide (NO) secretion in LPS-stimulated RAW264.7 macrophage cells. This marked suppression of NO production underscores their considerable anti-inflammatory potential.

**Figure 4:**
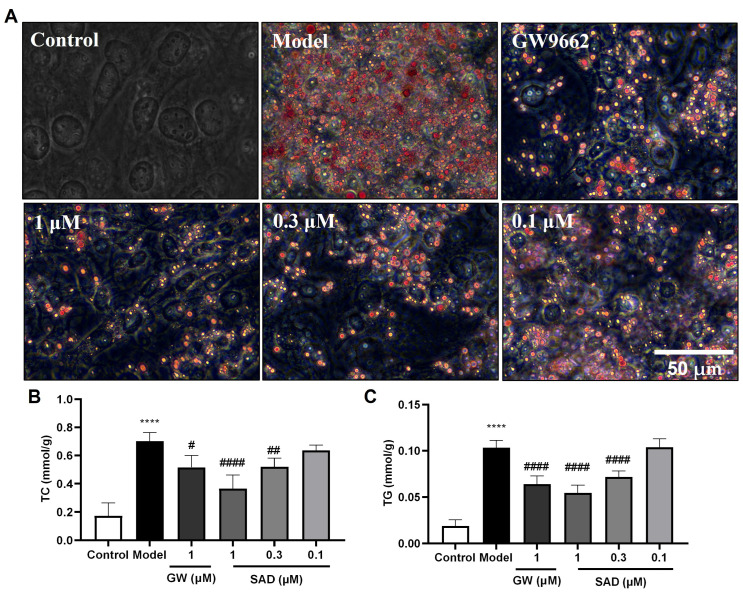
Effects of compound **4** (SAD) on lipid accumulation in 3T3-L1 adipocytes, with GW9662 as a positive control. (**A**) Oil Red O staining of lipid droplets in 3T3-L1 cells treated with various concentrations of compound **4** (0.1, 0.3, and 1 µM) after induction of adipogenesis. The images show the control group (no treatment), model group (no compound treatment), GW9662 (GW, 1 μM; reduced lipid accumulation as a PPARγ antagonist benchmark), and cells treated with different concentrations of the compound **4**. The scale bar represents 50 µm. (**B**) Quantification of total cholesterol (TC) levels in the treated cells. (**C**) Quantification of triglyceride (TG) levels in the treated cells. Data are expressed as the mean ± standard error medium (n = 6). Statistical significance is indicated as follows: **** *p* < 0.0001 versus the control group; # *p* < 0.05, ## *p* < 0.01, and #### *p* < 0.0001 versus the model group.

We further evaluated the anti-adipogenic activities of compounds **1**–**20** by quantification of total cholesterol (TC) and triglyceride (TG) levels as well as using Oil Red O staining in 3T3-L1 preadipocytes cells. Among them, Compound **4** (Secalonic acid D, SAD) exhibited the strongest inhibitory effect on lipid droplet accumulation (Figure 4). To benchmark SAD’s efficacy, we included GW9662 (1 μM), a selective PPARγ antagonist known to inhibit adipogenesis, as a positive control [42]. The results indicated that SAD significantly inhibited the differentiation of 3T3-L1 preadipocytes into adipocytes and reduced lipid droplet formation in a dose-dependent manner (0.1–1 μM) (Figure 4). Oil Red O staining revealed a marked reduction in lipid droplets in SAD-treated cells, comparable to or exceeding that of GW9662 (Figure 4A). Quantitative assays corroborated these findings, showing substantially decreased intracellular TC (Figure 4B) and TG levels (Figure 4C) with SAD treatment, with effects at 1 μM approaching those of the positive control. Notably, these effects were achieved at sub-micromolar concentrations (<1 μM) without observable cytotoxicity, positioning SAD as a promising candidate for anti-obesity therapeutics targeting metabolic dysregulation. The potent lipid-lowering activity positions SAD as a promising candidate for further development of anti-obesity therapeutics targeting metabolic dysregulation. Further studies are ongoing to elucidate the detailed mechanism of action of SAD and to explore its in vivo anti-obesity efficacy. These findings collectively demonstrate the versatility of *Talaromyces stipitatus*-derived compounds in addressing both inflammatory and metabolic disorders, highlighting their potential therapeutic applications.

The intricate and now well-established link between chronic low-grade inflammation and metabolic diseases, a concept often termed "meta-inflammation," has revolutionized our understanding of obesity [43,44]. In this paradigm, adipose tissue is not merely an energy storage depot but a dynamic endocrine and immunological organ. In obese states, hypertrophied adipocytes and infiltrating immune cells, particularly macrophages, create a pro-inflammatory microenvironment by secreting cytokines. This localized inflammation could spill over into systemic circulation, driving insulin resistance and perpetuating a vicious cycle of metabolic dysfunction. This research provides a strong rationale for a dual-pronged therapeutic strategy, which discovering agents that can either directly quell inflammation or inhibit adipogenesis, thereby disrupting the cycle from two different pathways. Our findings from the marine-derived fungus *Talaromyces stipitatus* HF05001 contributed novel insights into this very strategy.

SAD (**4**) has previously been reported as a mycotoxin with teratogenic and cytotoxic properties at relatively high doses [45,46,47,48]. However, our data demonstrate potent anti-inflammatory (IC_50_ = 0.22 μM) and anti-adipogenic activity at sub-micromolar concentrations (<1 μM) that are >10-fold lower than those causing cytotoxicity in vitro. Furthermore, to quantitatively assess the safety margin of compound 4, we calculated its Therapeutic Index (TI) as the ratio of the IC_50_ for cytotoxicity to the IC_50_ for bioactivity. For anti-inflammatory activity in LPS-stimulated RAW264.7 macrophages, the TI was approximately 19 (cytotoxicity IC_50_ = 4.2 μM, anti-inflammatory IC_50_ for NO inhibition = 0.22 μM, Figure 3C). For anti-adipogenic activity in 3T3-L1 preadipocytes, the TI was approximately 14 (cytotoxicity IC_50_ = 13.4 μM; based on effective concentrations < 1 μM for lipid reduction, Figure 4B,C). These TI values indicate a reasonable therapeutic window, where bioactivity is achieved at concentrations 14–19-fold below those causing significant cytotoxicity, comparable to some natural product-derived agents like curcumin (TI ≈15–25 in similar models) [49]. Nonetheless, this window is relatively narrow for direct clinical application, underscoring the need for structural modifications to enhance selectivity and reduce off-target toxicity. These metrics reinforce compound 4 as a promising scaffold for anti-inflammatory and anti-obesity drug development, with ongoing studies focused on in vivo pharmacokinetics and analog synthesis to widen the TI. This pronounced therapeutic window suggests that appropriate structural modification or targeted delivery strategies could mitigate toxicity while preserving bioactivity, a common approach in natural-product-based drug development (e.g., paclitaxel, artemisinin). 

Building on these findings, while the precise molecular mechanisms underlying the anti-inflammatory and anti-adipogenic activities of compound 4 remain to be fully elucidated, preliminary inferences can be drawn from the data and related literature. The suppression of NO and TNF-α (Figure 3B–D) suggests interference with LPS-induced NF-κB signaling, potentially reducing downstream mediators like PGE2 and IL-6, as observed in similar fungal metabolites [50]. For anti-adipogenesis, SAD’s dose-dependent reduction in lipid accumulation (Figure 4), comparable to the PPARγ antagonist GW9662, implies possible modulation of key transcription factors such as PPARγ and C/EBPα during early (days 0–2) and late (days 4–8) stages of 3T3-L1 differentiation. These mechanistic insights, supported by the inclusion of GW9662 as a benchmark, provide a foundation for understanding SAD’s dual activities in meta-inflammation.

The unprecedented bis-spiroketal scaffold of Talarobispiral A (**17**) suggests a novel biosynthetic pathway within *Talaromyces fungi*. While no direct precursors were identified in this study, future genomic and enzymatic investigations are warranted to elucidate its intriguing biogenesis.

In summary, this work transcends a simple report of new compounds. By screening for dual bioactivities within the unifying framework of meta-inflammation, we have unearthed distinct molecular tools from *Talaromyces stipitatus* HF05001. We have identified both direct anti-inflammatory leads (**4**, **7** and **16**) and a highly promising, selective anti-adipogenic scaffold (**4**). Additionally, the novel bis-spiroketal architecture of compound **17** represents a significant contribution to the structural diversity of fungal natural products. Although compound **17** exhibits moderate anti-inflammatory activity compared to the potent known compounds **4**, **7**, and **16**, its unique bis-spiroketal architecture represents a valuable addition to the structural diversity of fungal natural products and may inspire future synthetic modifications for enhanced potency. This study not only expands the chemical diversity of marine natural products but also provides a clear, rational basis for developing novel therapeutics targeting the nexus of inflammation and obesity.

## 3. Materials and Methods

### 3.1. General Experimental Procedures

NMR spectra were recorded on a Bruker 600 MHz spectrometer (Bruker Company, Fällanden, Switzerland) with tetramethylsilane (TMS) as an internal standard. HRESIMS data were acquired using a Xevo G2 Q-TOF mass spectrometer (Waters, Milford, MA, USA). Semi-preparative HPLC was performed using an Alltech LS class pump with a model 201 variable wavelength UV/Vis detector, and a YMC packed ODS-A (250 × 10 mm, 5 µm) column (YMC Co., Ltd., Kyoto, Japan) was used for purification. Column chromatography (CC) was carried out using a Sephadex LH-20 (Amersham Biosciences, San Francisco, CA, USA), ODS-A-HG (YMC Co., Ltd., Kyoto, Japan), and silica gel (Qingdao Marine Chemistry Co., Ltd., Qingdao, China). TLC analyses were performed with precoated silica gel plates by heating after spraying with vanillin sulfuric acid chromogenic reagent (Xilong Scientific Co., Ltd., Shantou, China).

### 3.2. Fungal Material and Identification

The fungus *Talaromyces stipitatus* HF05001 was isolated from a sponge sample collected near Zhanjiang, Guangdong, China. The fungus was identified based on morphological characteristics and ITS region sequencing. The ITS gene sequence was deposited in GenBank and assigned the accession number KU057945.1. The fungus was preserved at the laboratory of Technical Innovation Center for Utilization of Marine Biological Resources, Third Institute of Oceanography, Ministry of Natural Resources.

### 3.3. Fermentation, Extraction, and Isolation

The fungus was cultivated in a potato dextrose agar (PDA) plate under 25 °C for four days. The fresh mycelia and spores were inoculated into 500 mL Erlenmeyer flasks, each containing 100 mL potato dextrose broth (PDB) medium and followed by cultivation in a rotary shaker under 25 °C at 200 rpm for four days. The seed cultures were subsequently inoculated to 30 Erlenmeyer flasks (1 L) after autoclaving at 121 °C for 22 min. The agar-malt paste medium was prepared by mixing 20 g/L mannitol, 20 g/L maltose, 10 g/L glucose, 10 g/L monosodium glutamate, 1 g/L corn steep liquor, 3 g/L yeast extract, 0.5 g/L KH_2_PO_4_, and 0.3 g/L MgSO_4_·7H_2_O in seawater, and the pH was adjusted to 7.5. The fermentation was performed under static conditions at 25 °C for 35 days. The fermented material was fragmented using a stick and extracted successively with EtOAc three times, yielding an EtOAc extract. The extract was subjected to vacuum liquid chromatography column on silica gel eluting with a gradient of CH_2_Cl_2_ and MeOH (1:0 to 0:1) to furnish fractions. The fractions were subsequently separated by CC on ODS with MeOH/H_2_O elution (30–100%) to obtain subfractions. Further purification was carried out using semi-preparative HPLC with varying ratios of MeOH/H_2_O to yield the compounds.

Talarobispiral A (**17**): pale-yellow oil; ^1^H and ^13^C NMR data, see Table 1; HRESIMS *m/z* 349.2015 [M+H]⁺ (calcd for C_20_H_29_O_5_, 349.2010), 371.1832 [M+Na]⁺ (calcd for C_20_H_29_O_5_Na, 371.1829).

### 3.4. RAW264.7 Cell Culture and Treatment

The RAW264.7 macrophage cells were cultured in DMEM medium containing 10% fetal bovine serum and antibiotics (100 units/mL of penicillin and 100 g/mL of streptomycin) and maintained in a humidified 5% CO_2_ incubator at 37 °C. For the experiment, cells were seeded into 24-well plates (2 × 10^4^ cells/well) overnight. Next day, cells were incubated with fresh culture medium containing indicated concentration of the tested compounds for half an hour and following LPS treatment (1 µg/mL). Cells were treated with vehicle (DMSO, 0.1%) as a control.

### 3.5. Nitrite Quantification

The concentration of nitrite in culture medium was determined using a Griess Reagent Kit (Thermo Fisher, Shanghai, China) [51,52]. Briefly, 75 µL of cell culture supernatants were reacted with an equal volume of Griess Reagent Kit for 30 min at room temperature, and absorbance of diazonium was obtained at a wavelength of 560 nm. Nitrite production by vehicle stimulation was designated as 100% inhibition compared to LPS stimulation for the experiment.

### 3.6. 3T3-L1 Anti-Adipogenic Assay

The anti-adipogenic activity was evaluated using the 3T3-L1 preadipocyte cell line. 3T3-L1 cells were maintained in DMEM containing 10% calf serum and 1% penicillin-streptomycin at 37 °C in a 5% CO_2_ atmosphere. For differentiation, cells were induced with a mixture of 0.5 mM 3-isobutyl-1-methylxanthine (IBMX), 1 μM dexamethasone, and 10 μg/mL insulin. After 48 h, the medium was replaced with a maintenance medium containing 10 μg/mL insulin. During this differentiation process, compounds were added at concentrations of 1 µM. After 8 days, differentiated adipocytes were washed with PBS, fixed in 4% paraformaldehyde (15 min), and stained with filtered Oil Red O working solution (Solution A: B = 3:2; Nanjing Jiancheng Bioengineering, Cat# D027) for 20 min at RT. After destaining with 60% isopropanol, lipid droplets were visualized by bright-field microscopy (Zeiss, Imager.A2, Jena, Germany). 

### 3.7. Triglyceride (TG) and Total Cholesterol (TC) Quantification

Cell lysates were prepared by washing differentiated adipocytes with cold PBS, followed by scraping, centrifugation (1000× *g*, 10 min, 4 °C), and resuspension in 200 μL PBS. Samples were sonicated on ice (3 × 10-s pulses at 40% amplitude). TG and TC levels were determined enzymatically using commercial kits (Nanjing Jiancheng Bioengineering; TG: Cat# A110-1-1, TC: Cat# A111-1-1) according to manufacturer protocols. Briefly, for TG analysis, 2.5 μL lysate was combined with 250 μL lipase/glycerol kinase-based reagent, incubated at 37 °C for 10 min, and absorbance measured at 500 nm with quantification against a glycerol standard. For TC measurement, 2.5 μL lysate was reacted with 250 μL cholesterol oxidase-phenol-4-aminoantipyrine (COD-PAP) reagent at 37 °C for 10 min, followed by absorbance reading at 500 nm and quantification using cholesterol standards. All values were normalized to total protein content.

### 3.8. NMR Calculation

The conformational analysis of the isomers was first conducted via random searching in the Stochastic using the MMFF94 force field with an energy cut-off of 5.0 kcal/mol and an RMSD threshold of 0.2 A. All conformers were consecutively optimized at the PM6 and HF/6-31G(d)levels. Dominative conformers were further optimized at the B3LYP/6-31G(d) level in the gas phase. The optimized conformers possess no imaginary frequencies and are true local minima. NMR calculations were then carried out by the Gauge-Including Atomic Orbitals (GIAO) method at MPW1PW91/6–311+G(2d,p) level in chloroform simulated by the IEFPCM model [53]. The TMS-corrected NMR chemical shift values were averaged according to Boltzmann distribution, finally the calculated ^13^C NMR data were obtained by the linear regression analysis method.

### 3.9. DP4+ Analysis

The ^1^H and ^13^C chemical shifts were computed from NMR calculations, then the counted and experimental chemical shifts were applied in the excel to use DP4+ calculations according to the Boltzmann statistics using the computed sum of electronic and zero-point energies as the input. On the DP4+ analysis excel, functional mPW1PW91, basis set 6- 311+G(d,p) and solvent PCM were chosen to calculate.

## 4. Conclusions

In conclusion, this study has unveiled the chemical diversity and bioactivity of compounds derived from the marine fungus *Talaromyces stipitatus* HF05001. The isolated compounds demonstrated significant anti-inflammatory and anti-adipogenic activities, with compound **4** (Secalonic acid D), compound **7** (Sch 725680) and compound **16** (Bacillisporin C) demonstrated potent anti-inflammatory activity, while compound **4** (Secalonic acid D) showing remarkable potential in inhibiting lipid accumulation in 3T3-L1 cells. These findings underscore the value of marine-derived fungi as a resource for novel bioactive compounds and pave the way for further exploration of their therapeutic applications in inflammatory and metabolic disorders.

## Figures and Tables

**Table 1:** ^1^H (600 MHz) and ^13^C (150 MHz) NMR spectroscopic data of compound **17** in CDCl_3_ (*δ* in ppm, *J* in Hz within parentheses).

No.	*δ*_C_	*δ*_H_
1	110.1 CH	6.34 (d, 2.4)
2	154.3 C	
3	101.8 CH	6.44 (d, 2.4)
4	151.8 C	
5	111.3 C	
6	138.2 C	
7	19.1 CH_3_	2.17 s
8	20.4 CH_2_	2.92 (dd, 16.8, 7.8) 2.19 (dd, 16.8, 2.4)
9	36.2 CH	2.29 m
10	62.0 CH_2_	3.68 (dd, 11.7, 11.8) 3.45 (dd, 11.7, 4.7)
11	36.0 CH_2_	1.73 (d, 8.5)
12	96.6 C	
13	42.7 CH_2_	2.48 (d, 13.8) 1.67 (d, 13.8)
14	96.7 C	
15	25.1 CH_2_	1.67 (13.2, 13.0) 1.46 (13.2, 4.6)
16	36.3 CH	1.53 m
17	65.5 CH_2_	3.56 (dd, 11.2, 4.5) 3.24 (dd, 11.2, 11.0)
18	25.2 CH_2_	1.16 m
19	11.1 CH_3_	0.88 (t, 7.5)
20	48.2 CH_3_	3.28 s

**Table 2:** Anti-inflammatory activities and cell viability inhibition of compounds **1**–**20** at 10 μM in LPS-stimulated RAW264.7 cells.

Compounds	NO Inhibition (%)	Cell Viability Inhibition (%)
BAY 11-7085	85.63 ± 5.48	1.51 ± 2.95
**1**	21.45 ± 11.96	5.31 ± 1.10
**2**	47.15 ± 4.59	−3.18 ± 0.89
**3**	20.57 ± 10.43	0.54 ± 0.44
**4**	90.04 ± 3.47 ^1^	98.21 ± 3.35 ^1^
**5**	23.58 ± 5.08	2.09 ± 3.36
**6**	36.10 ± 6.60	0.44 ± 1.53
**7**	85.25 ± 4.20 ^1^	−2.67 ± 1.55
**8**	1.55 ± 1.23	1.29 ± 1.84
**9**	7.29 ± 1.34	−4.51 ± 1.47
**10**	13.52 ± 0.91	−11.77 ± 3.46
**11**	13.75 ± 12.83	−8.98 ± 1.73
**12**	18.86 ± 11.56	−8.05±2.19
**13**	27.07 ± 7.55	−3.08 ± 1.14
**14**	24.59 ± 10.43	−10.47 ± 3.12
**15**	22.38 ± 6.45	−9.54 ± 3.9
**16**	81.97 ± 1.76 ^1^	−4.76 ± 2.06
**17**	36.10 ± 7.29	7.57 ± 3.69
**18**	2.56 ± 8.03	3.73 ± 2.22
**19**	1.58 ± 2.78	6.98 ± 1.87
**20**	3.21 ± 2.45	3.19 ± 1.81

^1^ Inhibition rate more than 50%.

## Data Availability

The data presented in this study are available on request from the corresponding author.

## Supplementary Materials

The following supporting information can be downloaded at https://www.mdpi.com/article/10.3390/md24010047/s1: Figures S1–S73: One-dimensional and two-dimensional NMR spectra along with HRESIMS spectra of compounds **17** and other compounds.
