# Supplementary material for: A Novel Bis-Spiroketal Scaffold and Other Secondary Metabolites from the Marine-Derived Fungus Talaromyces stipitatus HF05001: Structural Diversity and Bioactivities
Source: Mar Drugs. 2026 Jan 19;24(1):47. doi: 10.3390/md24010047 (PMC12843244; doi:10.3390/md24010047)
Supplement: Supplementary file 1 [file marinedrugs-24-00047-s001.zip › marinedrugs-3979632-supplementary.pdf]

Supplementary Materials

A Novel Bis-Spiroketal Scaffold and Other Secondary Metabolites from the Marine-Derived Fungus *Talaromyces stipitatus* HF05001: Structural Diversity and Bioactivities

Longhe Yang [1], Yan Qiu [2,3], Ying Liu [2], Xiaoyu Wei [1], Xiwen He [1], Yiling Wang [1], Yajun Yan [1], Kaikai Bai [1], Zhaokai Wang [1,*] and Jie Ren [2,3,*]

[1] Technical Innovation Center for Utilization of Marine Biological Resources, Third Institute of Oceanography, Ministry of Natural Resources, Xiamen, 361000, China

[2] School of Medicine, Xiamen University, Xiamen, 361102, China

[3] Xiamen Key Laboratory of Chiral Drugs, Xiamen, 361102, China

* Correspondence: wang@tio.org.cn (Z.W.); renjie_7912@xmu.edu.cn (J.R.)

Table of Contents

Figure S1. ^{1}H-NMR spectrum of Compound **17** 5

Figure S2. ^{13}C-NMR spectrum of Compound **17** 5

Figure S3. DEPT spectrum of Compound **17** 6

Figure S4. ^{1}H-^{1}H COSY spectrum of Compound **17** 6

Figure S5. HSQC spectrum of Compound **17** 9

Figure S6. HMBC spectrum of Compound **17** 13

Figure S7. HR-MS spectrum of Compound **17** 14

Figure S8. ^{1}H-NMR spectrum of Compound **1** 15

Figure S9. ^{13}C-NMR spectrum of Compound **1** 15

Figure S10. ^{1}H-NMR spectrum of Compound **2** 16

Figure S11. ^{1}H-NMR spectrum of Compound **3** 16

Figure S12. ^{1}H-NMR spectrum of Compound **4** 17

Figure S13. ^{13}C-NMR spectrum of Compound **4** 17

Figure S14. DEPT spectrum of Compound **4** 18

Figure S15. ^{1}H-^{1}H COSY spectrum of Compound **4** 18

Figure S16. HSQC spectrum of Compound **4** 19

Figure S17. HMBC spectrum of Compound **4** 19

Figure S18. ^{1}H-NMR spectrum of Compound **5** 20

Figure S19. ^{13}C-NMR spectrum of Compound **5** 20

Figure S20. DEPT spectrum of Compound **5** 21

Figure S21. HSQC spectrum of Compound **5** 21

Figure S22. HMBC spectrum of Compound **5** 22

Figure S23. ^{1}H^{-1}H COSY spectrum of Compound **5** 22

Figure S24. ^{1}H-NMR spectrum of Compound **6** 23

Figure S25. ^{13}C-NMR spectrum of Compound **6** 23

Figure S26. 1H-NMR spectrum of Compound **7** 24

Figure S27. ^{13}C-NMR spectrum of Compound **7** 24

Figure S28. ^{1}H-^{1}H COSY spectrum of Compound **7** 25

Figure S29. HSQC spectrum of Compound **7** 25

Figure S30. ^{1}H-NMR spectrum of Compound **8** 26
Figure S31. ^{1}H-NMR spectrum of Compound **9** 26
Figure S32. ^{13}C-NMR spectrum of Compound **9** 27
Figure S33. DEPT spectrum of Compound **9** 27
Figure S34. ^{1}H-NMR spectrum of Compound **10** 28
Figure S35. ^{1}H-NMR spectrum of Compound **11** 28
Figure S36. ^{1}H-NMR spectrum of Compound **11** 29
Figure S37. ^{1}H-NMR spectrum of Compound **11** 29
Figure S38. ^{1}H-NMR spectrum of Compound **12** 30
Figure S39. ^{13}C-NMR spectrum of Compound **12** 30
Figure S40. DEPT spectrum of Compound **12** 31
Figure S41. ^{1}H-NMR spectrum of Compound **13** 31
Figure S42. ^{13}C-NMR spectrum of Compound **13** 32
Figure S43. DEPT spectrum of Compound **13** 32
Figure S44. ^{1}H-NMR spectrum of Compound **14** 33
Figure S45. ^{13}C-NMR spectrum of Compound **14** 33
Figure S46. ^{1}H-NMR spectrum of Compound **15** 34
Figure S47. ^{13}C-NMR spectrum of Compound **15** 34
Figure S48. DEPT spectrum of Compound **15** 35
Figure S49. ^{1}H-^{1}H COSY spectrum of Compound **15** 35
Figure S50. HSQC spectrum of Compound **15** 36
Figure S51. HMBC spectrum of Compound **15** 36
Figure S52. ^{1}H-NMR spectrum of Compound **16** 37
Figure S53. 1H-NMR spectrum of Compound **16** 37
Figure S54. ^{13}C-NMR spectrum of Compound **16** 38
Figure S55. ^{13}C-NMR spectrum of Compound **16** 38
Figure S56. DEPT spectrum of Compound **16** 39
Figure S57. ^{1}H-^{1}H COSY spectrum of Compound **16** 39
Figure S58. HSQC spectrum of Compound **16** 40
Figure S59. HMBC spectrum of Compound **16** 40
Figure S60. ^{1}H-NMR spectrum of Compound **18** 41

Figure S61. ^{13}C-NMR spectrum of Compound **18** 41
Figure S62. ^{1}H-NMR spectrum of Compound **19** 42
Figure S63. ^{13}C-NMR spectrum of Compound **19** 42
Figure S64. DEPT spectrum of Compound **19** 43
Figure S65. ^{1}H-^{1}H COSY spectrum of Compound **19** 43
Figure S66. HSQC spectrum of Compound **19** 44
Figure S67. HMBC spectrum of Compound **19** 44
Figure S68. ^{1}H-NMR spectrum of Compound **20** 45
Figure S69. ^{13}C-NMR spectrum of Compound **20** 45
Figure S70. DEPT spectrum of Compound **20** 46
Figure S71. ^{1}H-^{1}H COSY spectrum of Compound **20** 46
Figure S72. HSQC spectrum of Compound **20** 47
Figure S73. HMBC spectrum of Compound **20** 47
Figure S74: Cytotoxicity effects of compounds 1–20 in RAW264.7 cells. Cell viability was assessed using a CCK-8 assay, with the results presented as a percentage of the vehicle group. **** $p < 0.0001$ versus the vehicle group. 48
Supplemental Table 1 Detailed DP4+ probability for compound 17. Isomer is 9*S**,12*S**,14*S**,16*S**. 49

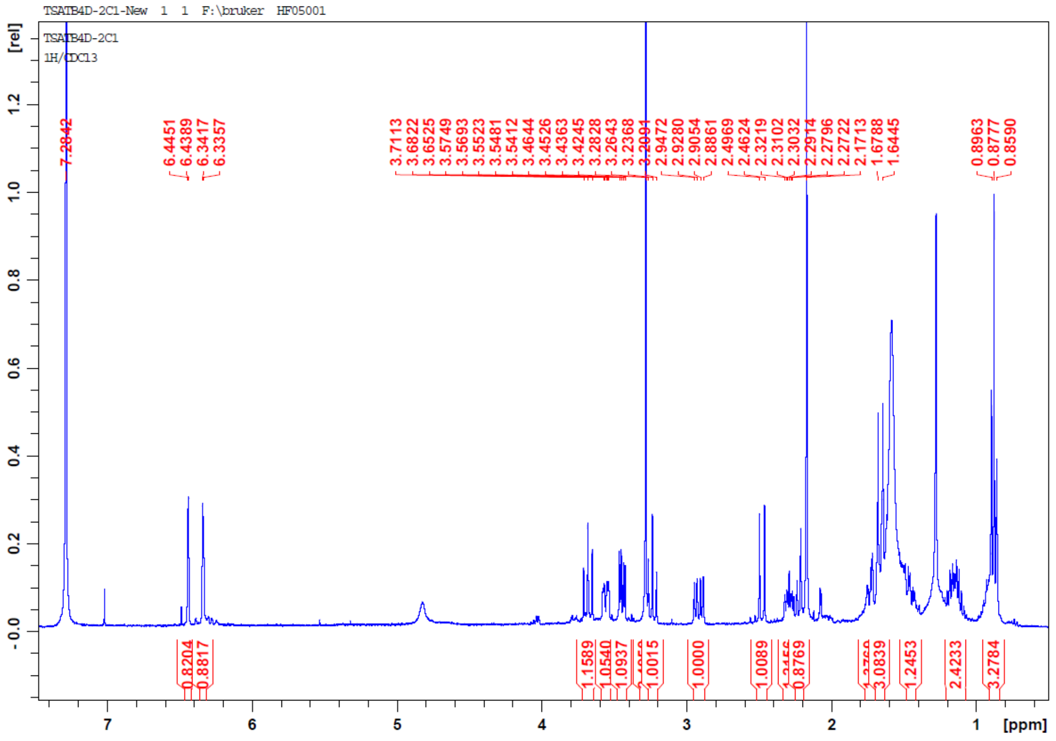

Figure S1. ^{1}H-NMR spectrum of Compound **17**

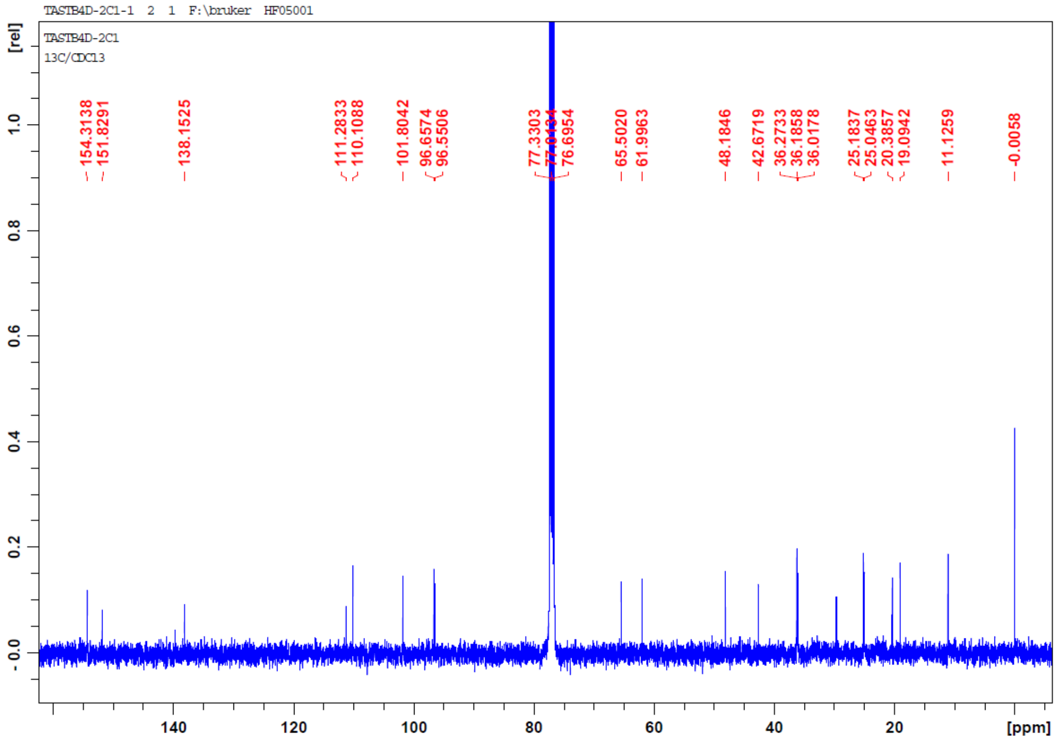

Figure S2. ^{13}C-NMR spectrum of Compound **17**

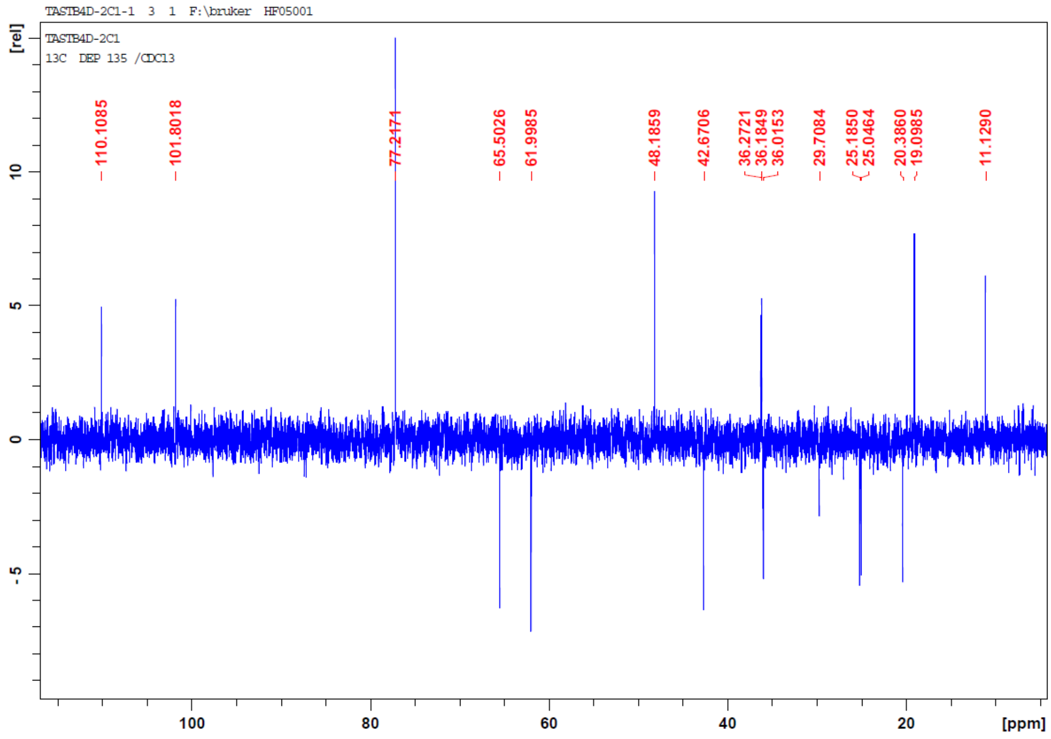

Figure S3. DEPT spectrum of Compound **17**

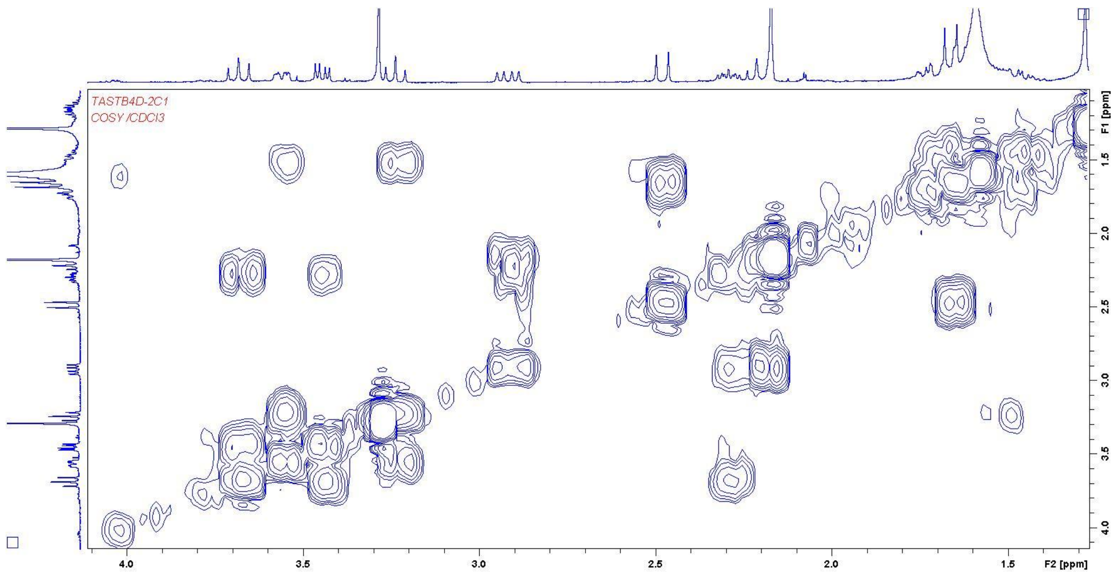

Figure S4. ^{1}H-^{1}H COSY spectrum of Compound **17**

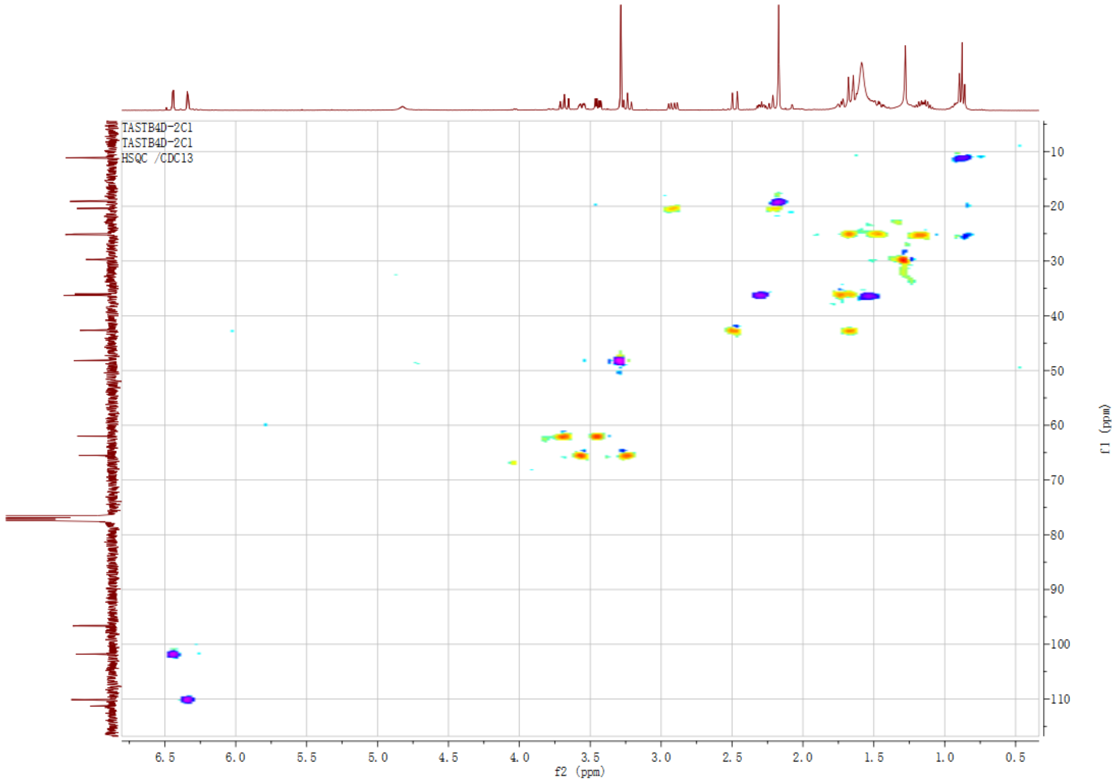
TASTB4D-2C1
TASTB4D-2C1
HSQC /CDCl3
f2 (ppm)
f1 (ppm)

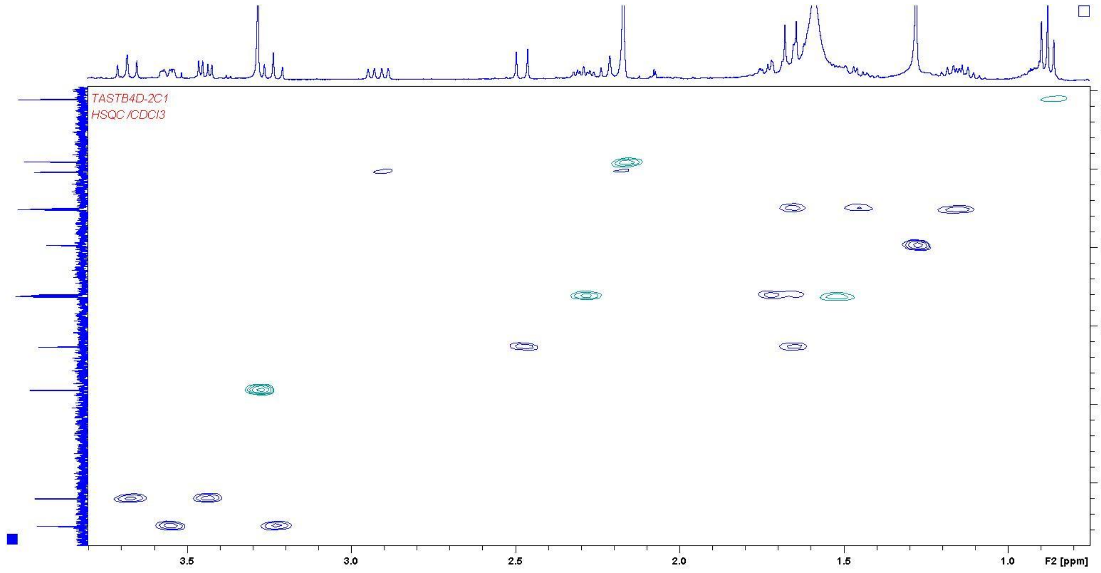
TASTB4D-2C1
HSQC /CDCl3
F2 [ppm]
F1 [ppm]

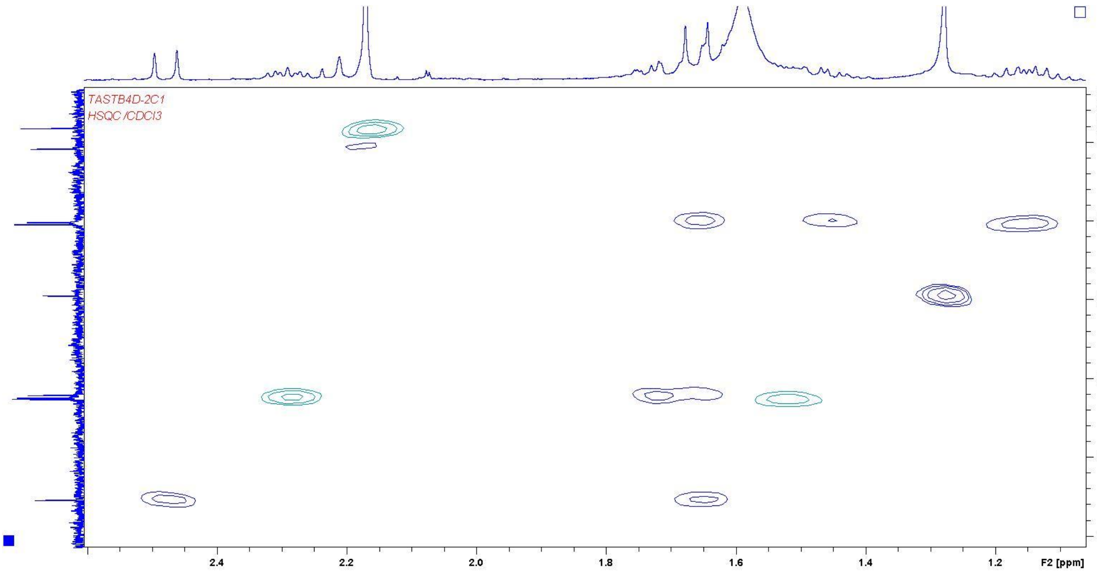
TASTB4D-2C1
HSQC /CDCl3
F1 [ppm]
F2 [ppm]

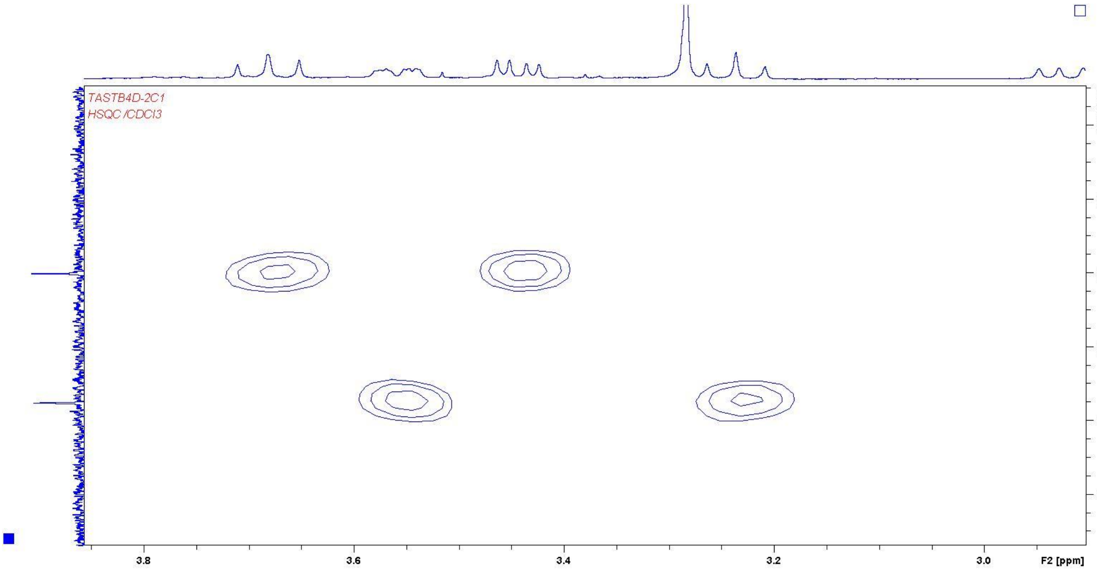
TASTB4D-2C1
HSQC /CDCl3
F1 [ppm]
F2 [ppm]

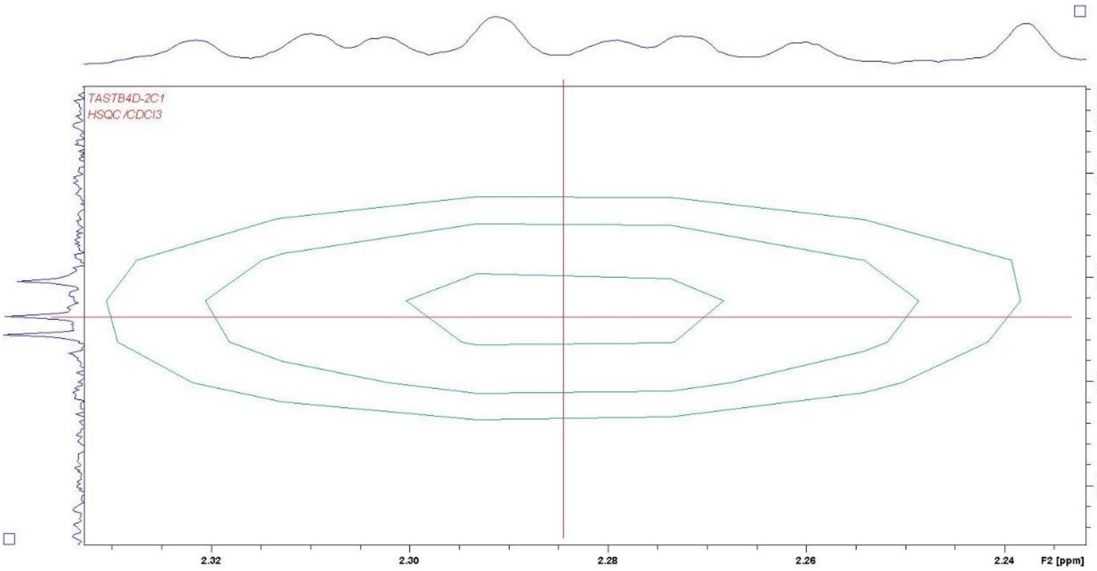
TASTB4D-2C1
HSQC /CDCl3
F1 [ppm]
F2 [ppm]

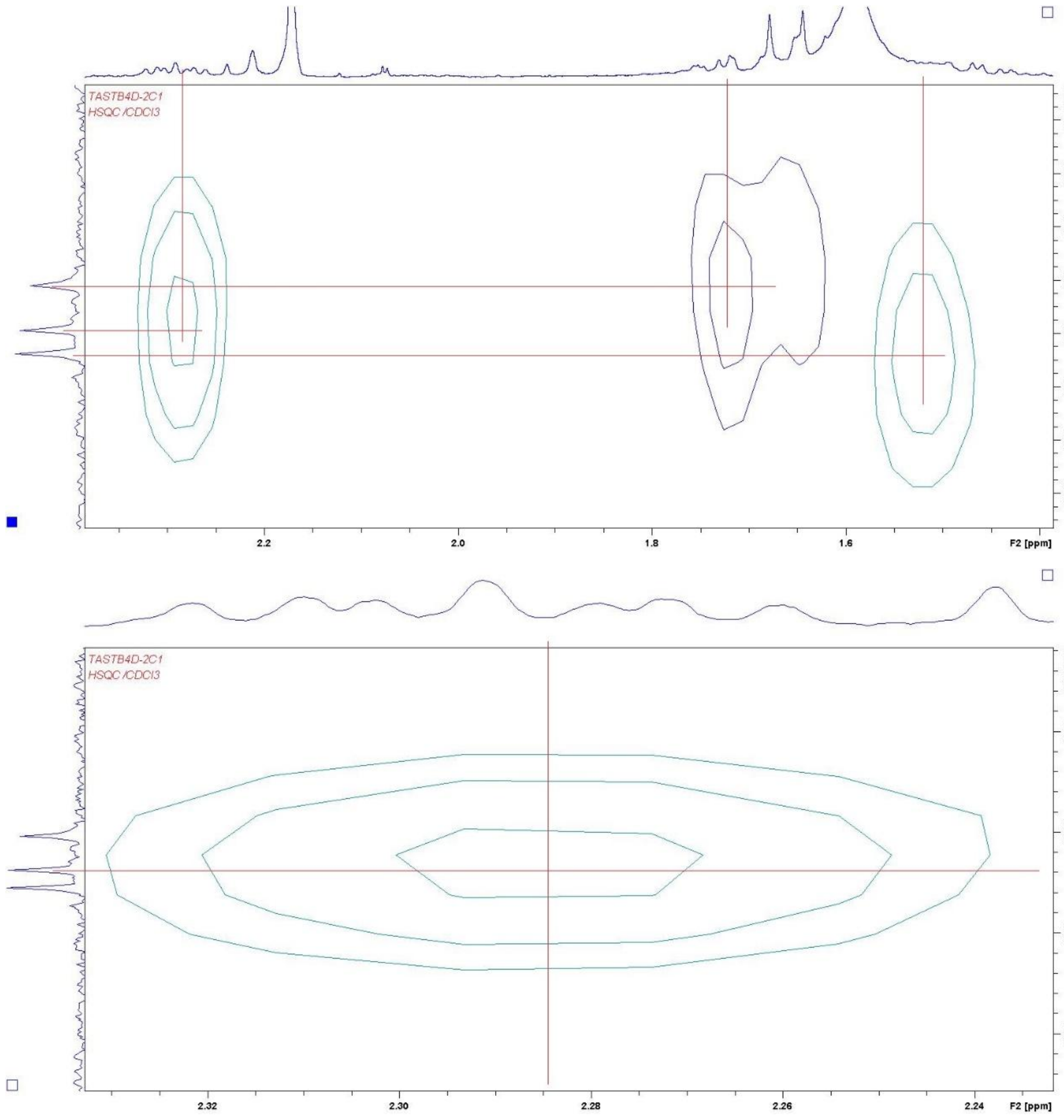

Figure S5. HSQC spectrum of Compound **17**

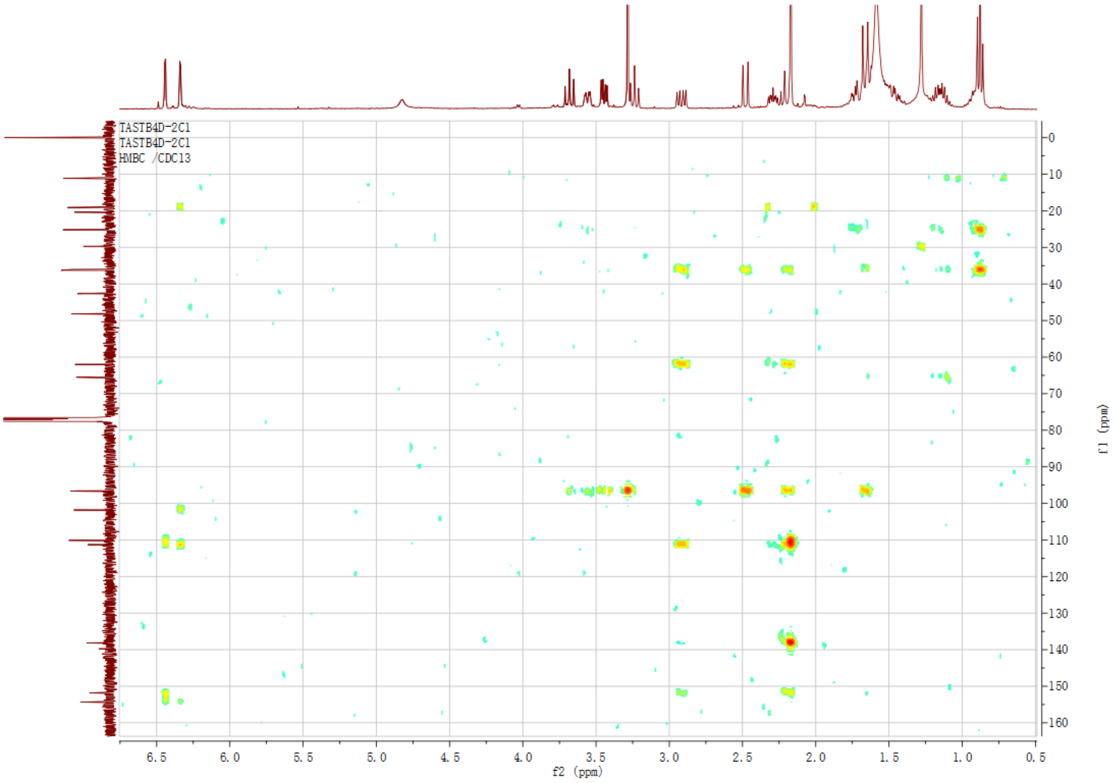
TASTB4D-2C1
TASTB4D-2C1
HMBC /CDCl3
f2 (ppm)
f1 (ppm)

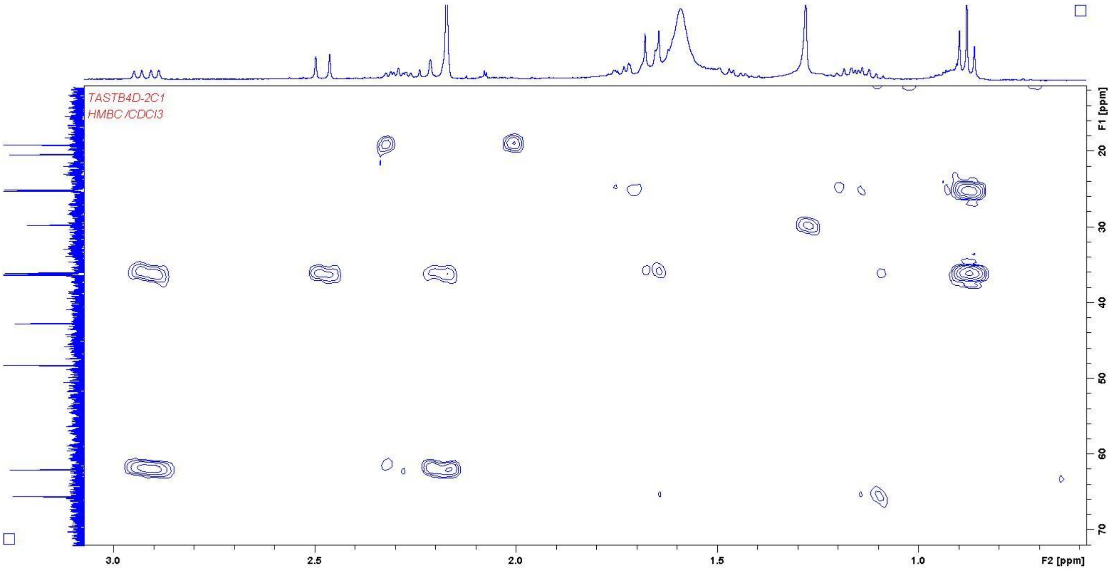
TASTB4D-2C1
HMBC /CDCl3
F1 [ppm]
F2 [ppm]

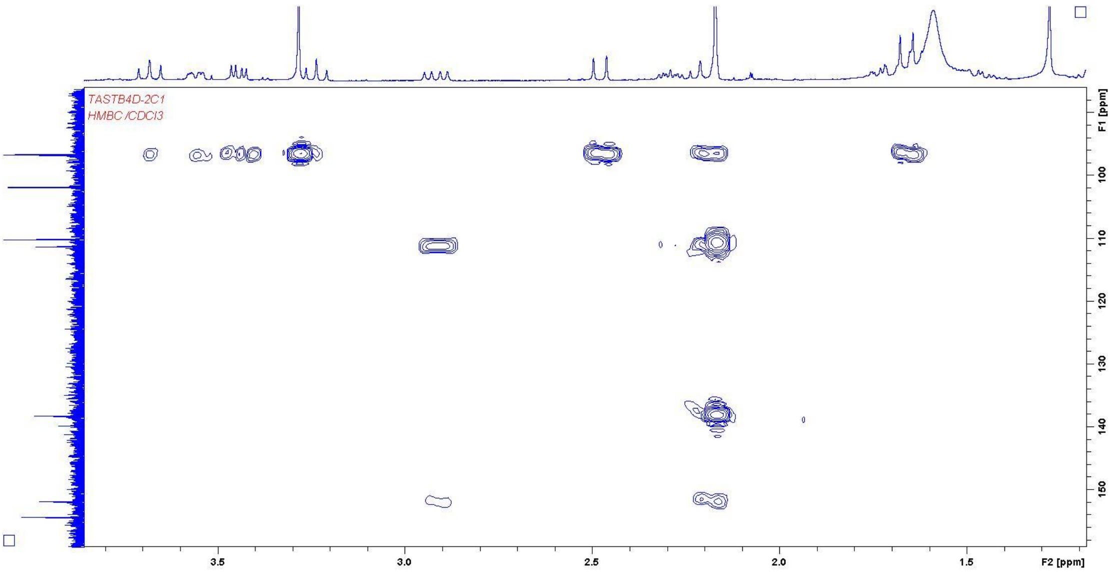
TASTB4D-2C1
HMBC /CDCl3
F1 [ppm]
F2 [ppm]
3.5
3.0
2.5
2.0
1.5
100
110
120
130
140
150

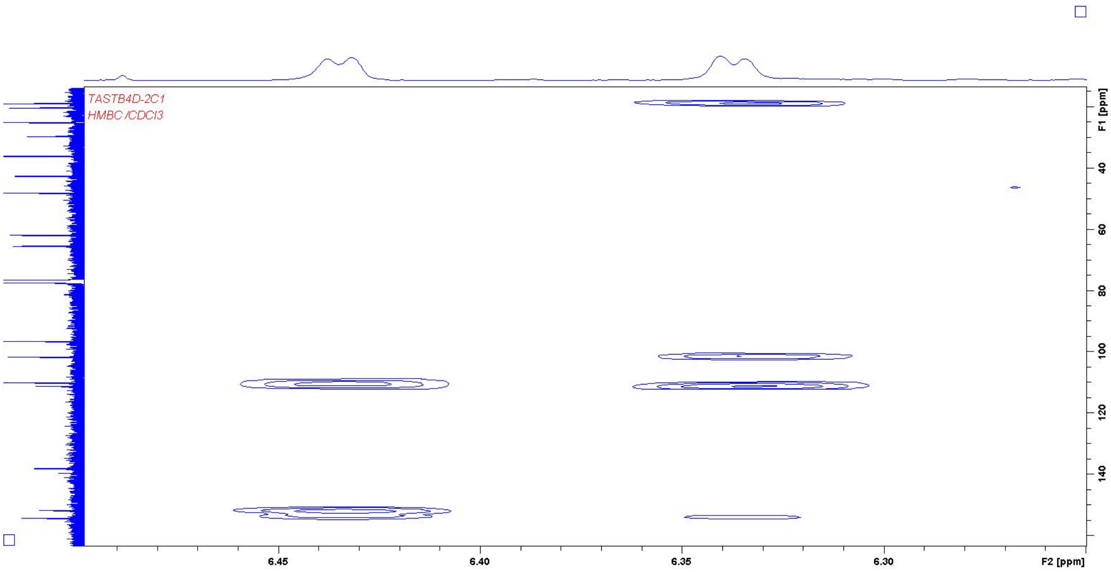
TASTB4D-2C1
HMBC /CDCl3
F1 [ppm]
F2 [ppm]
6.45
6.40
6.35
6.30
40
60
80
100
120
140

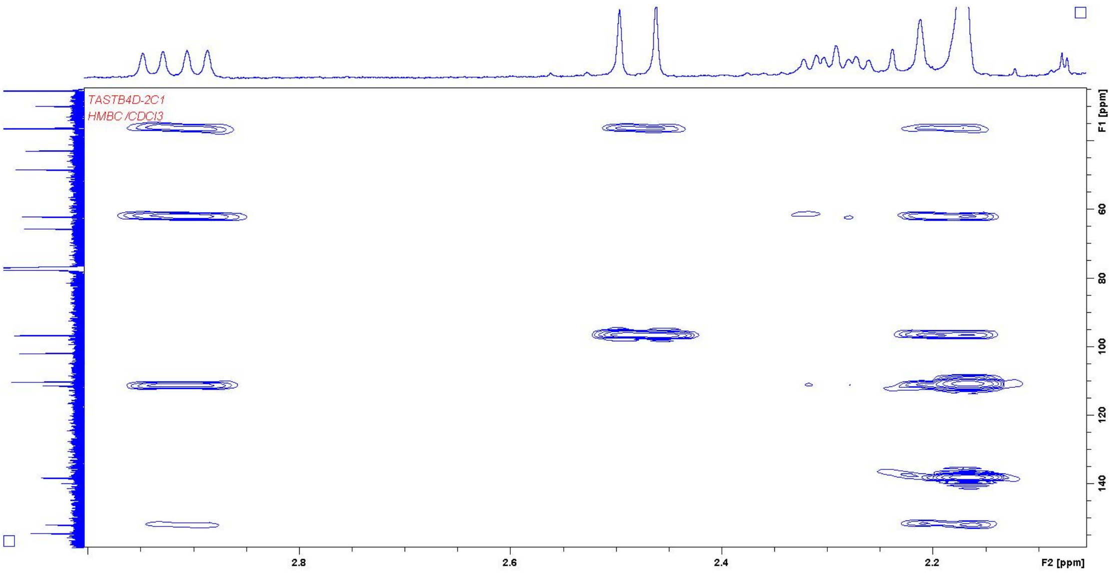
TASTB4D-2C1
HMBC /CDCl3
F1 [ppm]
F2 [ppm]
2.8
2.6
2.4
2.2
60
80
100
120
140

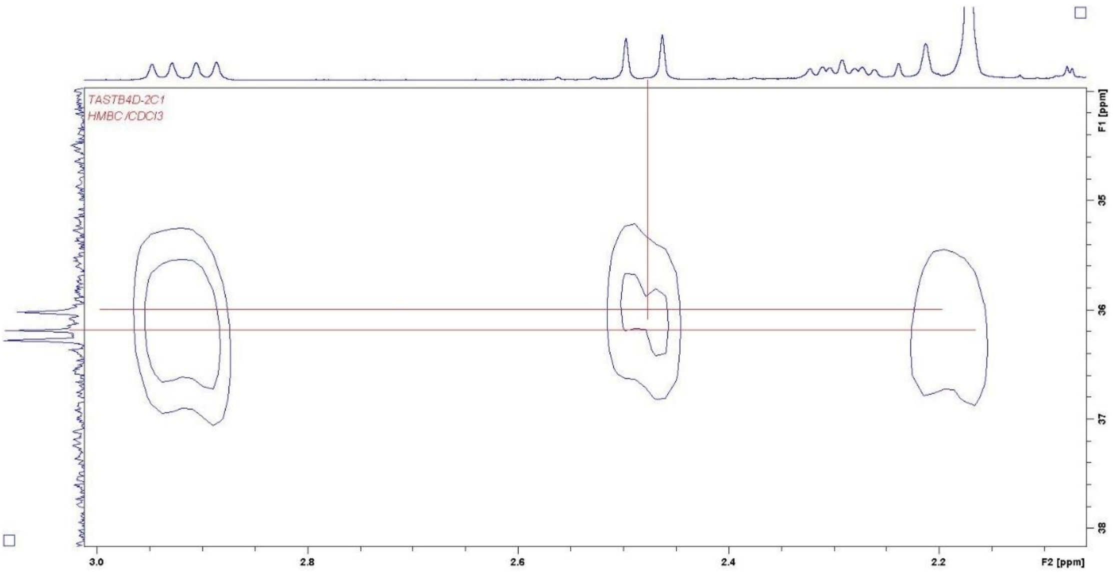
TASTB4D-2C1
HMBC /CDCl3
F1 [ppm]
F2 [ppm]

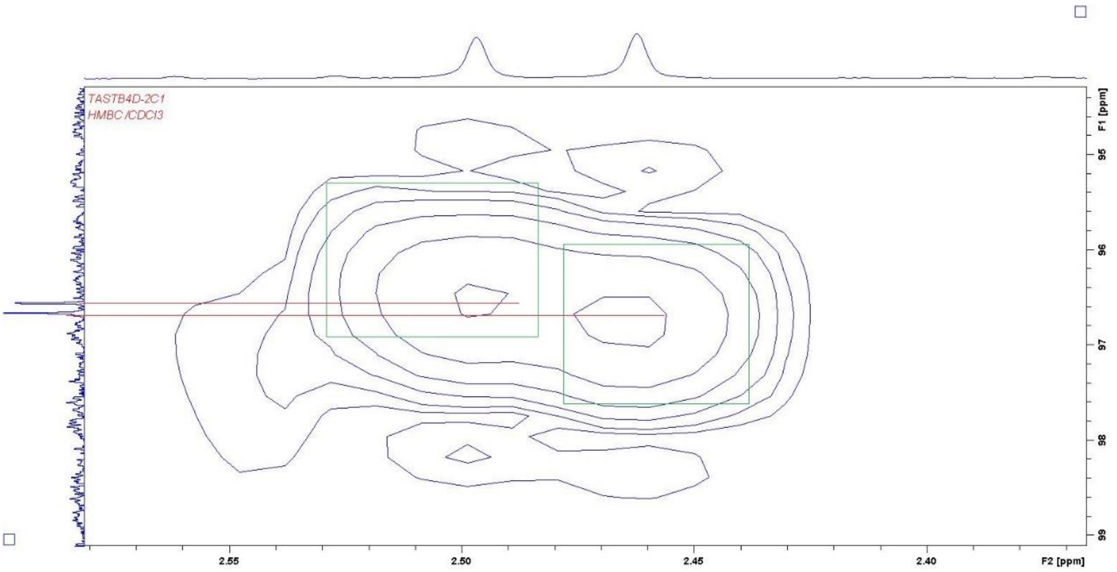
TASTB4D-2C1
HMBC /CDCl3
F1 [ppm]
F2 [ppm]

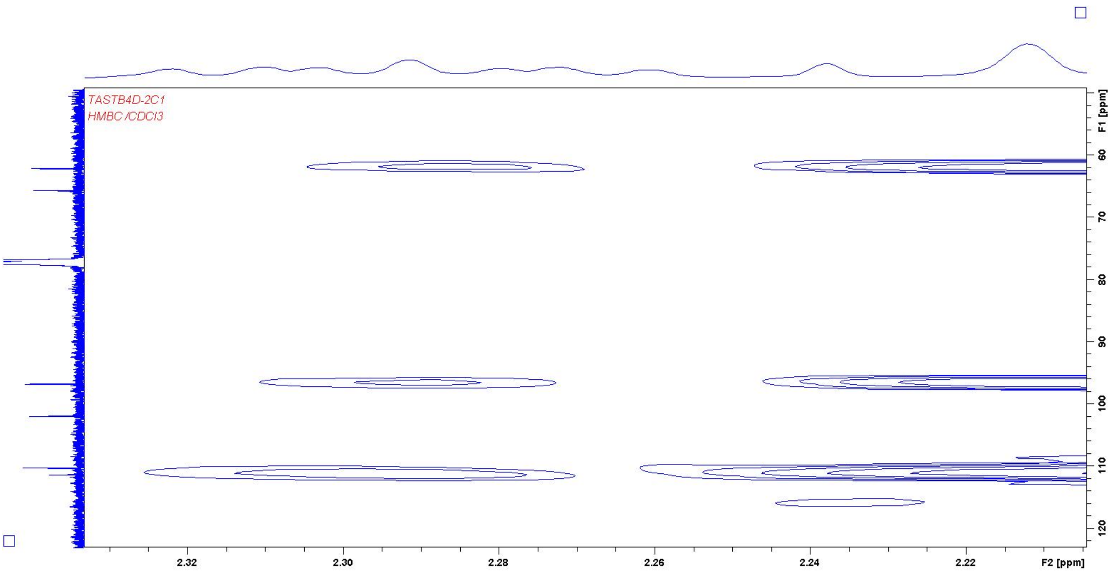
TASTB4D-2C1
HMBC /CDCl3
F1 [ppm]
F2 [ppm]

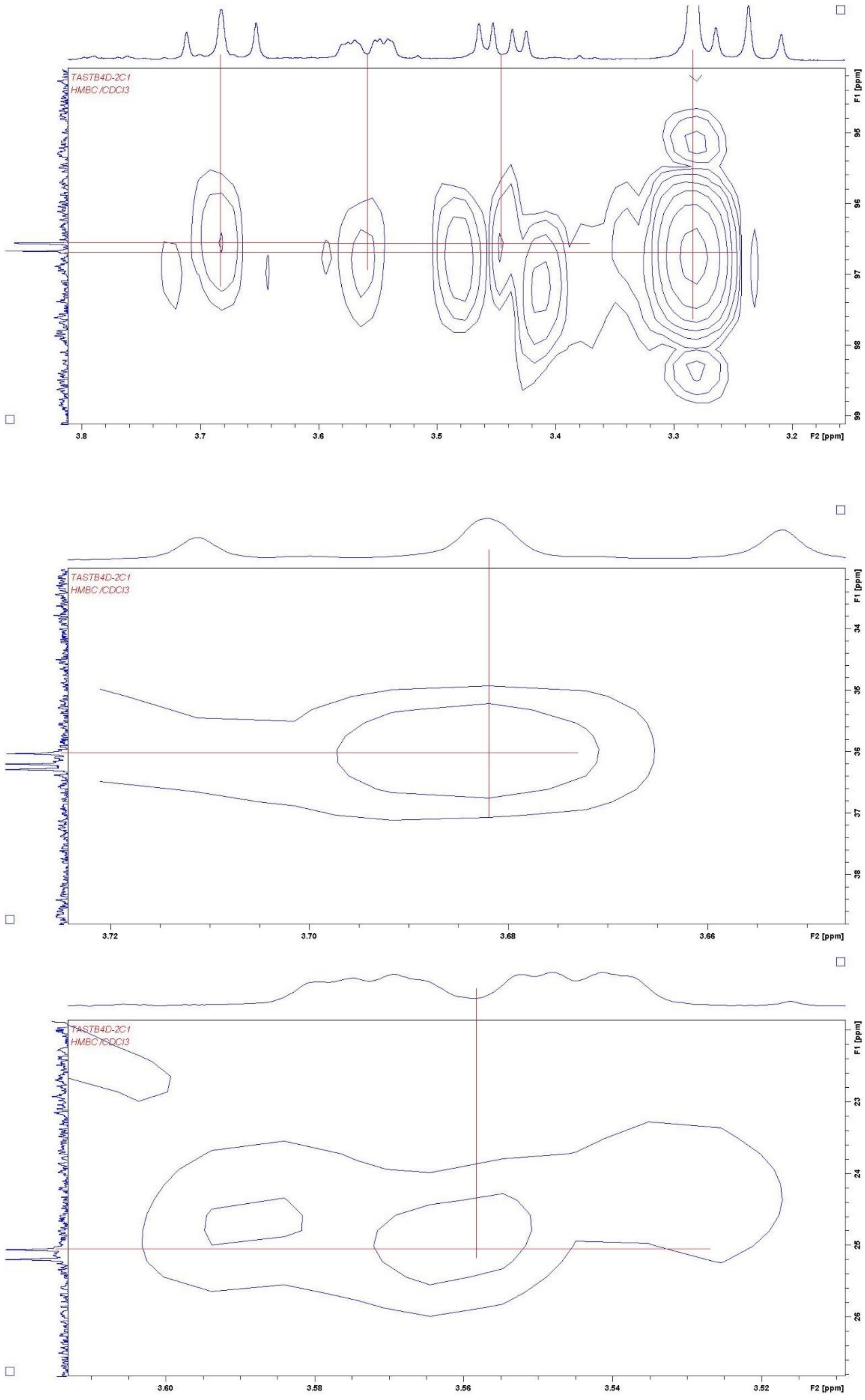

Figure S6. HMBC spectrum of Compound **17**

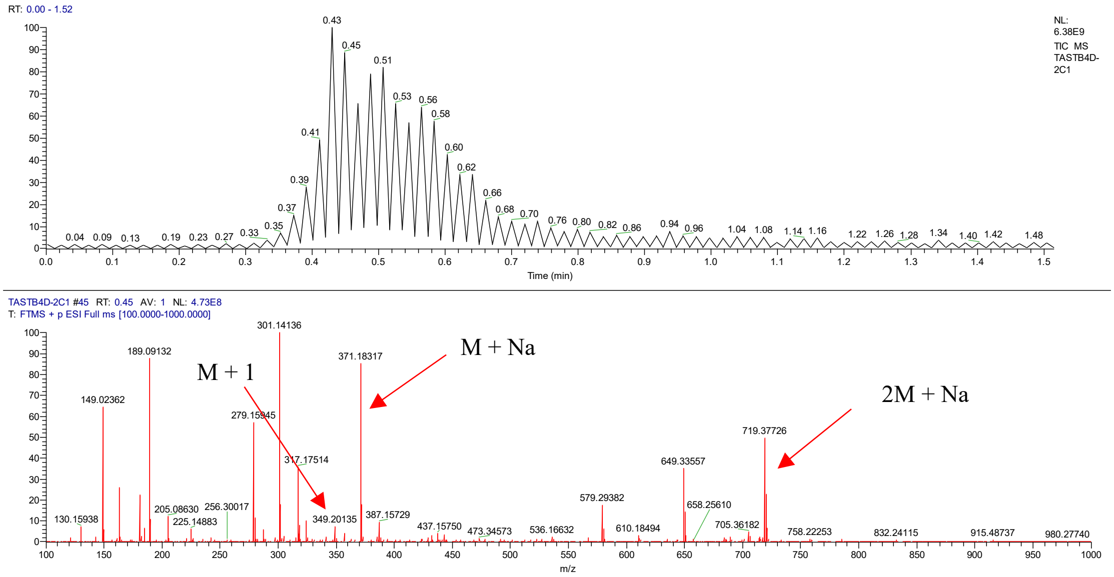

349.20135

1	C20 H29 O5	6.5	0.0004
2	C18 H30 O5 Na	3.5	0.0028
3	C16 H31 O5 Na2	0.5	0.00521
4	C27 H25	15.5	0.00627
5	C18 H32 O2 Na3	1.5	-0.00764
6	C25 H26 Na	12.5	0.00868
7	C20 H31 O2 Na2	4.5	-0.01005
8	C23 H27 Na2	9.5	0.01108
9	C22 H30 O2 Na	7.5	-0.01245
10	C21 H28 Na3	6.5	0.01349
11	C24 H29 O2	10.5	-0.01486

371.18317

1	C21 H27 O2 N Na2	8	0
2	C20 H28 O5 Na	6.5	0.00027
3	C19 H22 N7 Na	12	0.00028
4	C18 H23 O3 N6	10.5	0.00055
5	C20 H25 O4 N3	10	-0.00079
6	C21 H24 O N4 Na	11.5	-0.00106
7	C19 H25 O N4 Na2	8.5	0.00134
8	C7 H26 O7 N9 Na	-1	-0.00157
9	C18 H26 O4 N3 Na	7	0.00162
10	C8 H25 O4 N10 Na2	0.5	-0.00185

Figure S7. HR-MS spectrum of Compound **17**

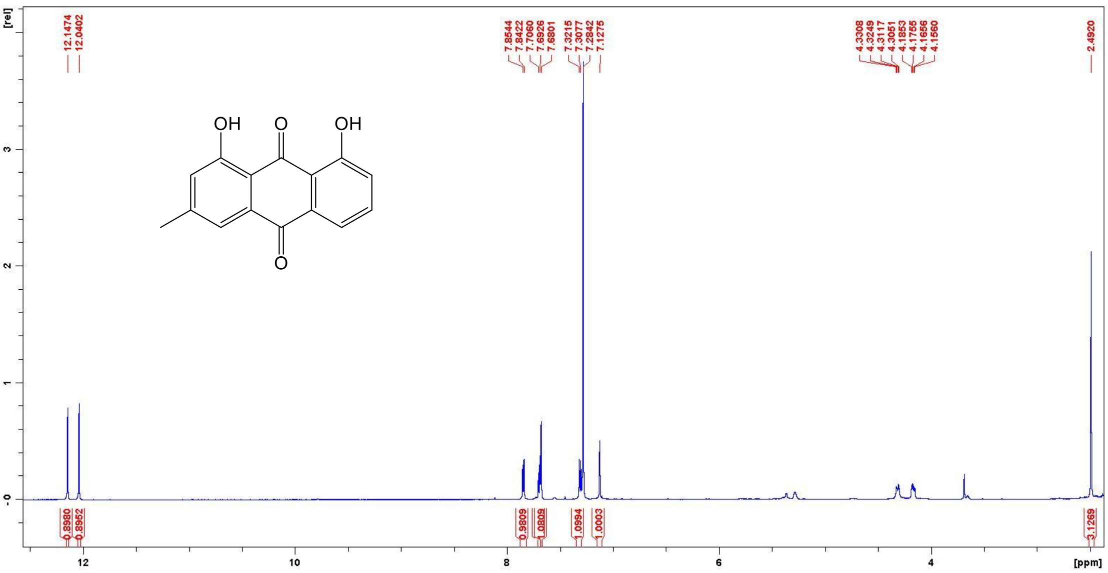

Figure S8. ^{1}H-NMR spectrum of Compound **1**

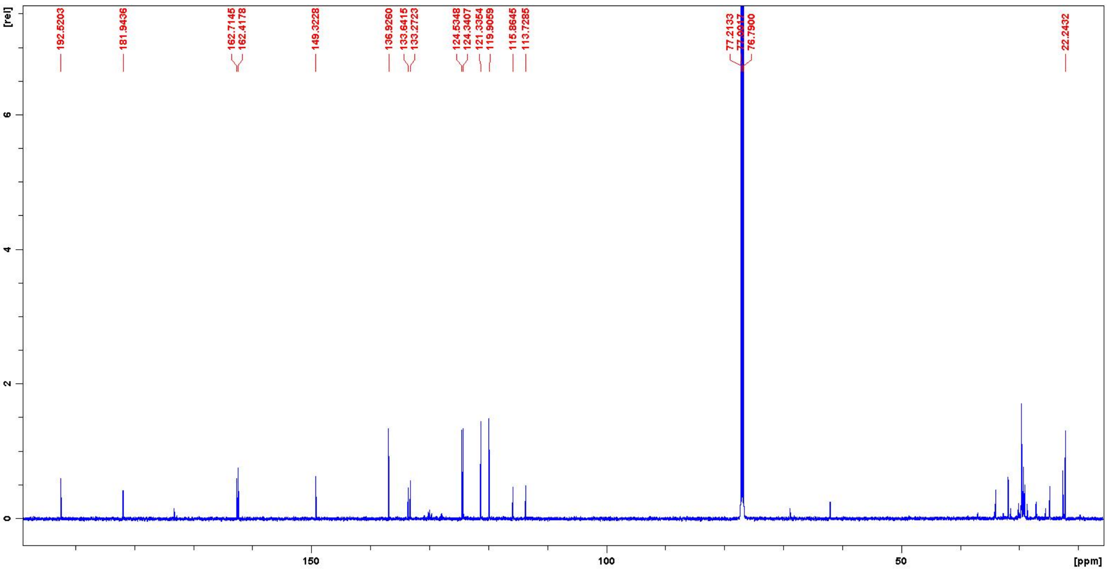

Figure S9. ^{13}C-NMR spectrum of Compound **1**

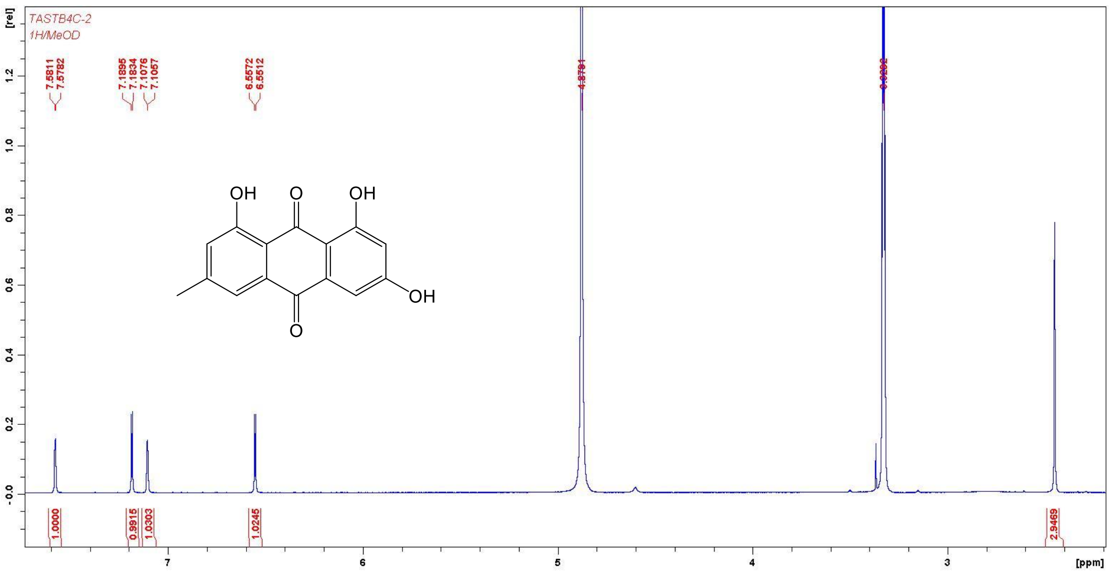

Figure S10. ^{1}H-NMR spectrum of Compound **2**

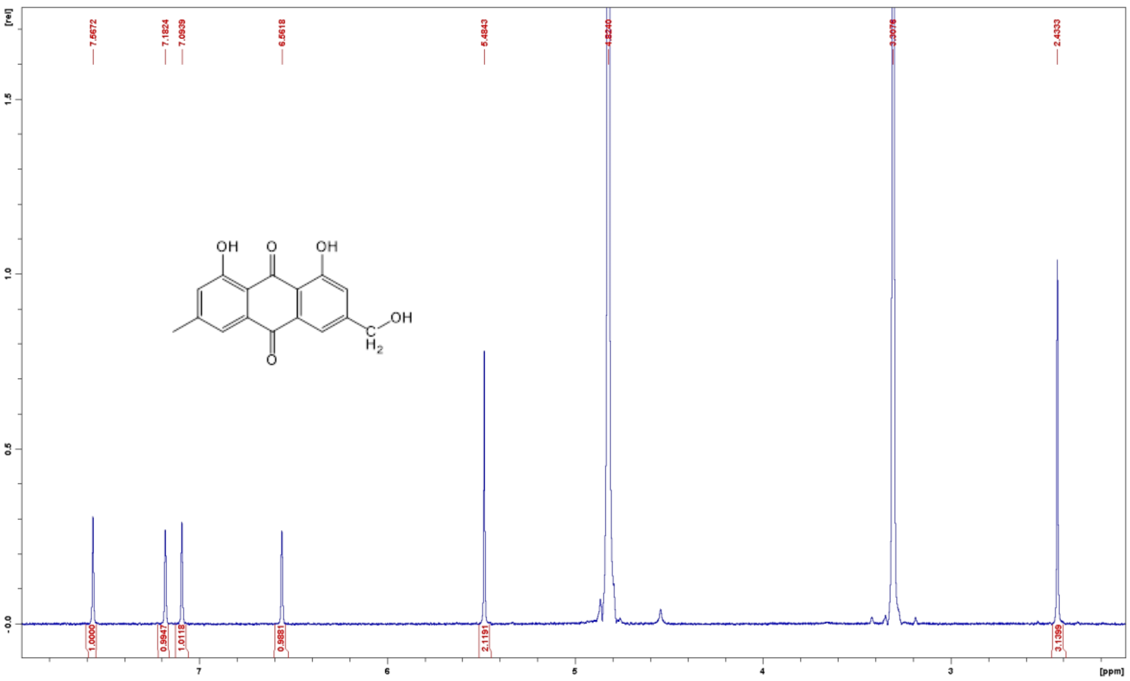

Figure S11. ^{1}H-NMR spectrum of Compound **3**

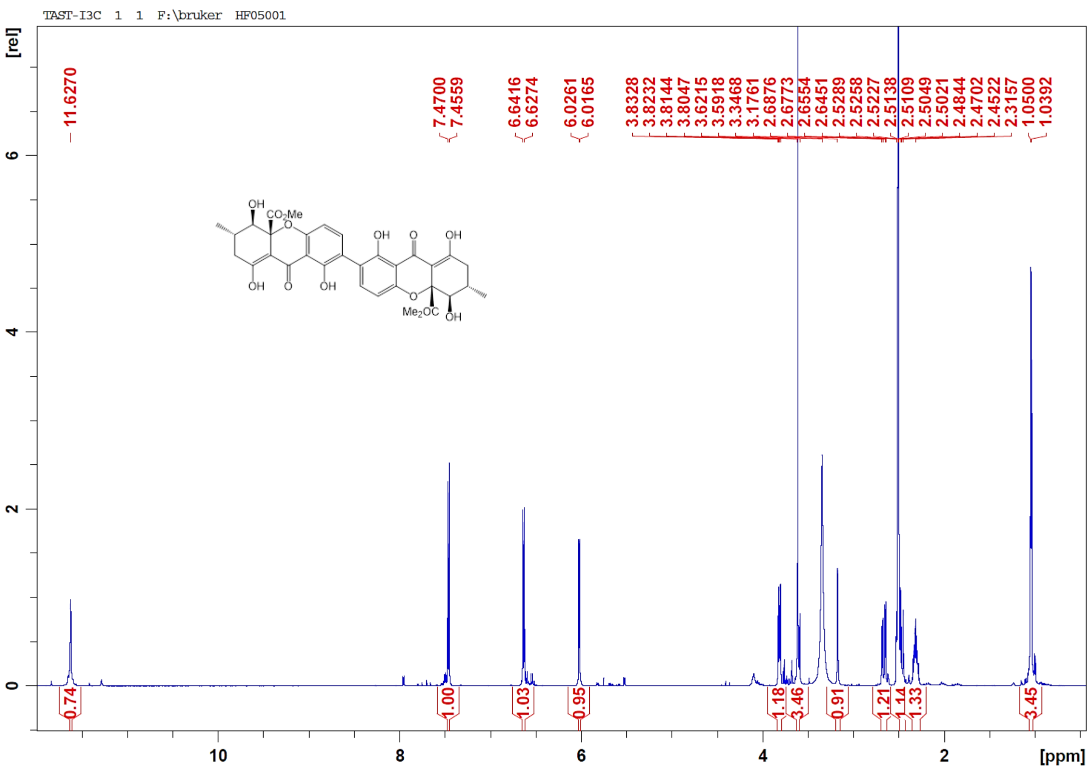

Figure S12. ^{1}H-NMR spectrum of Compound **4**

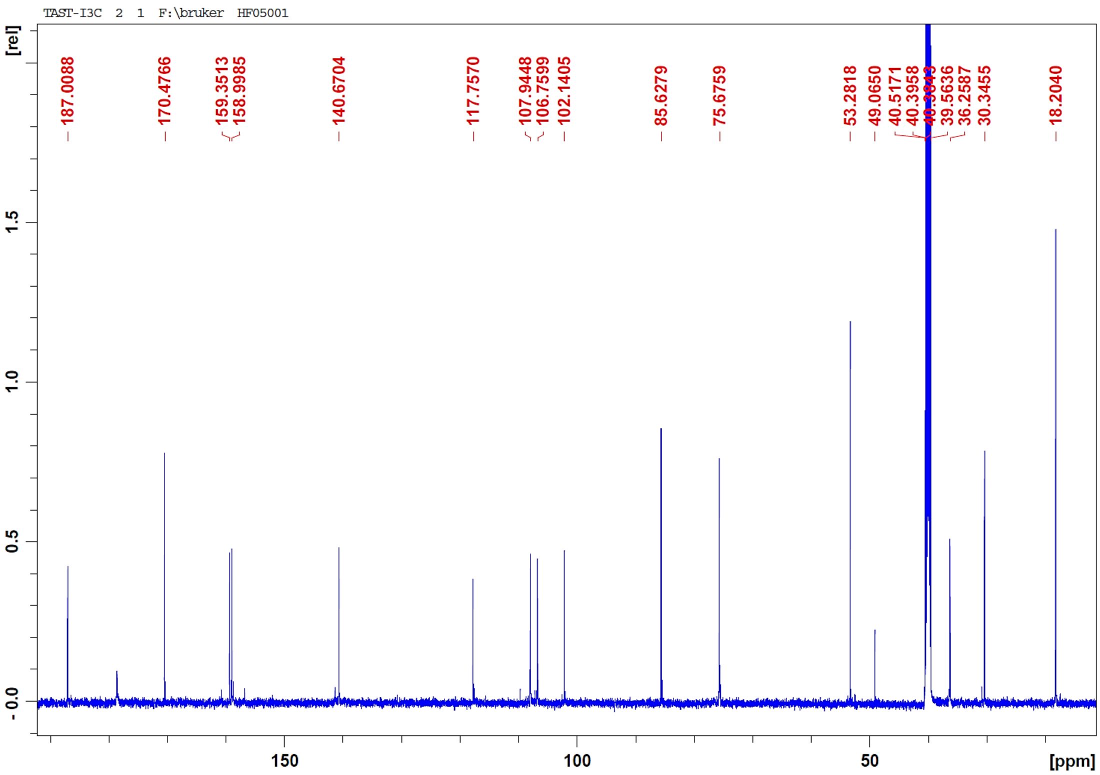

Figure S13. ^{13}C-NMR spectrum of Compound **4**

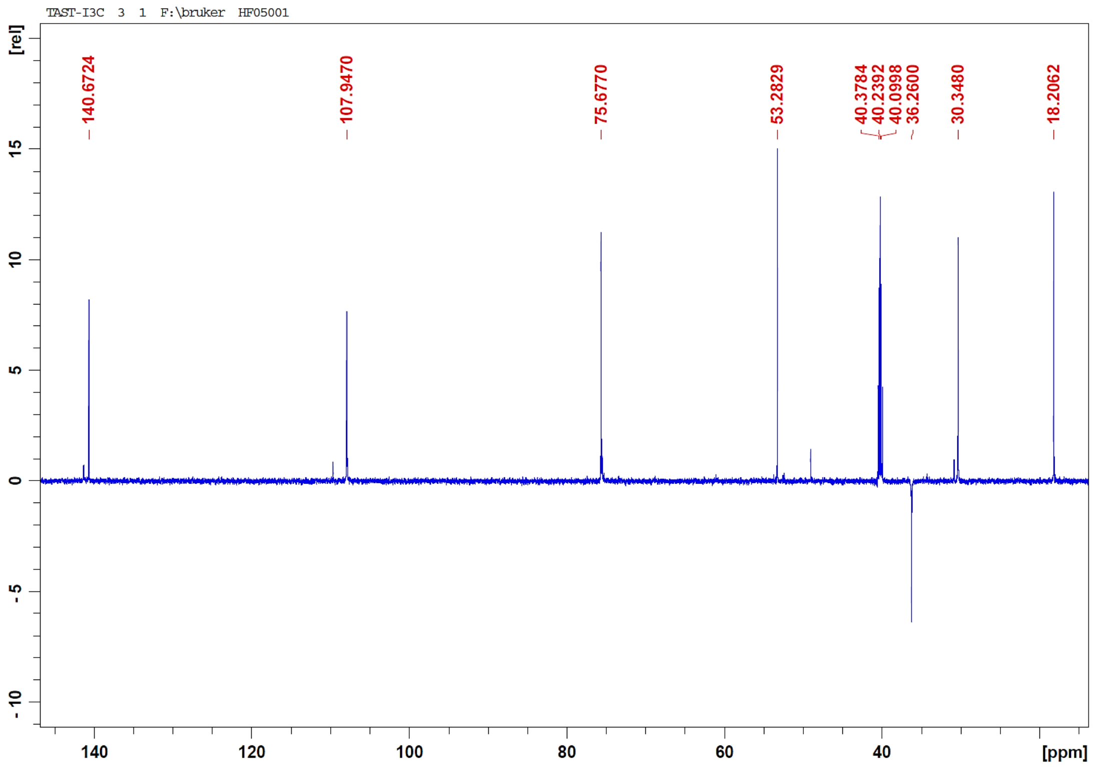

Figure S14. DEPT spectrum of Compound **4**

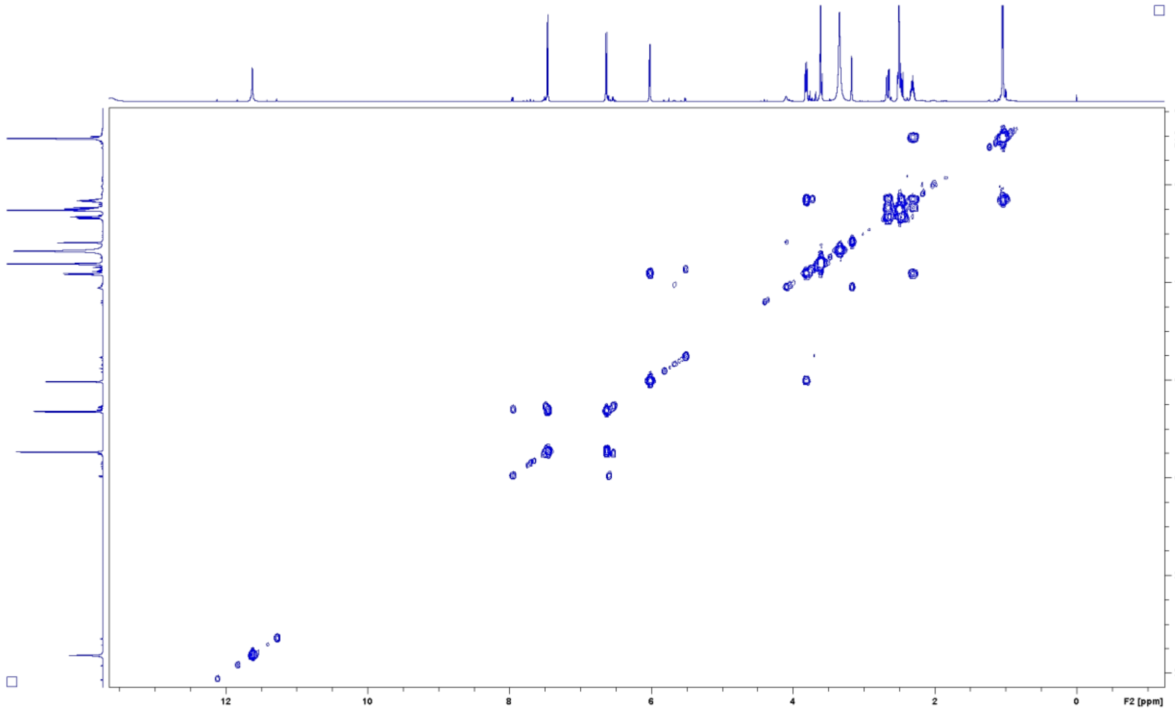

Figure S15. ^{1}H-^{1}H COSY spectrum of Compound **4**

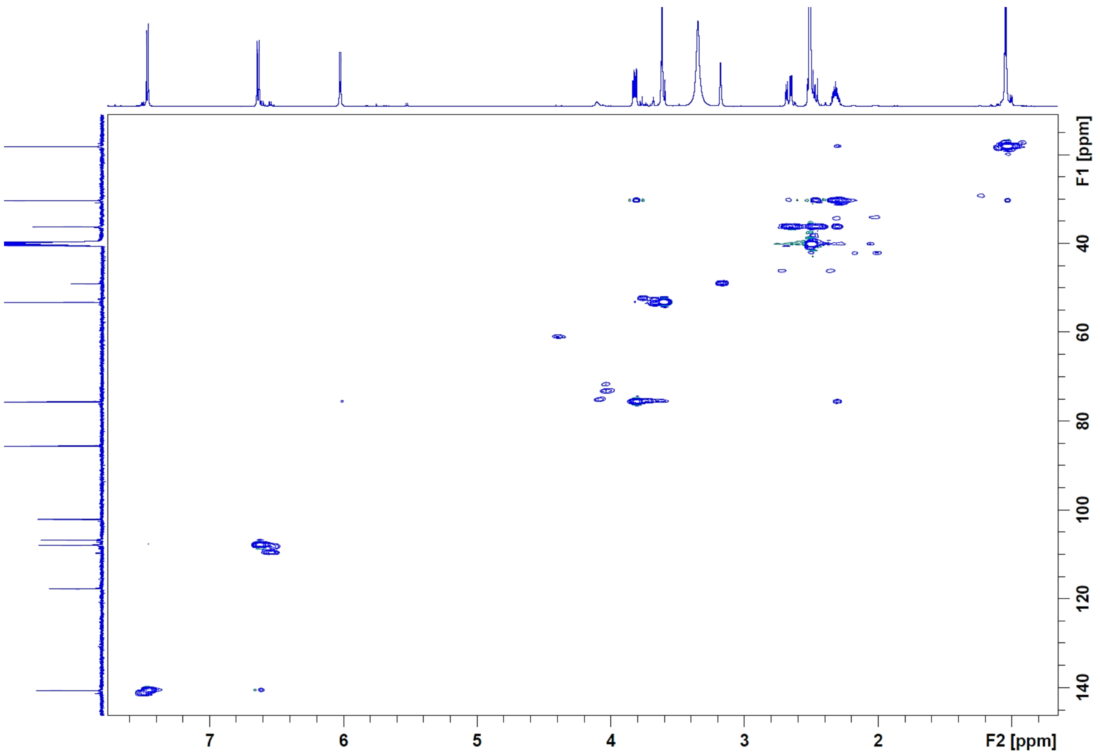

Figure S16. HSQC spectrum of Compound **4**

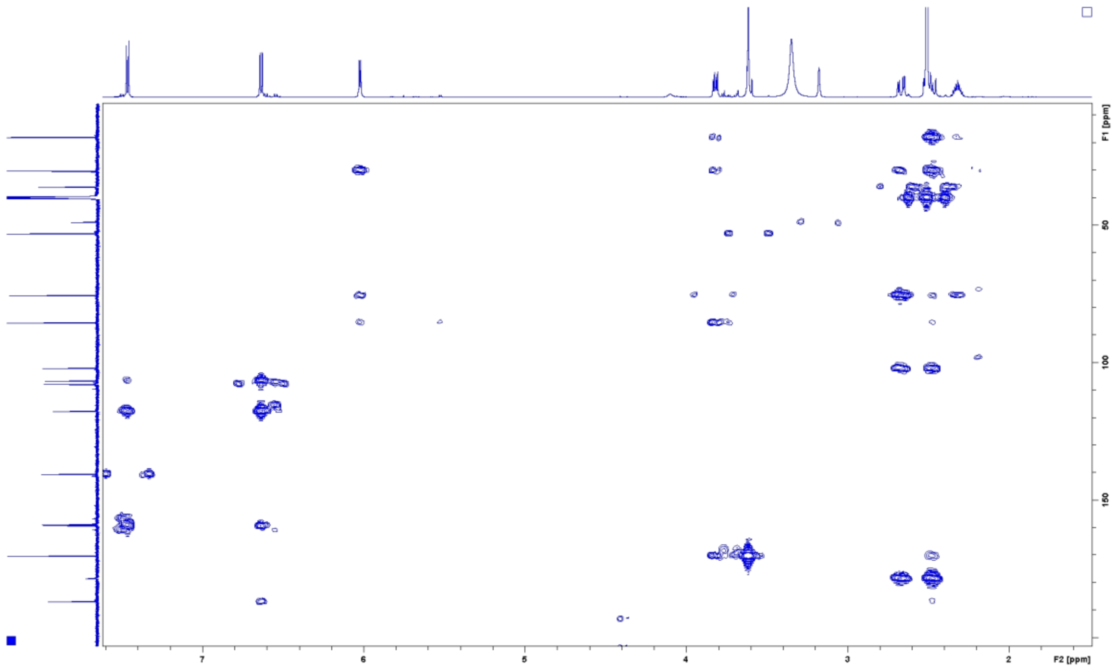

Figure S17. HMBC spectrum of Compound **4**

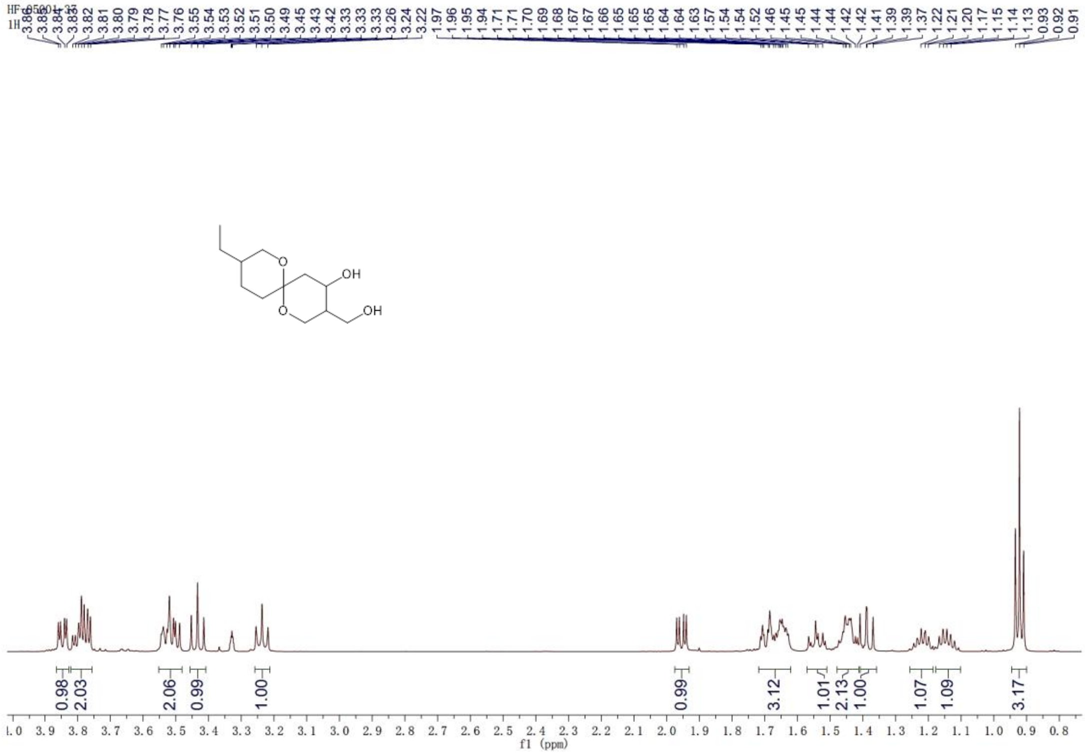

Figure S18. ^{1}H-NMR spectrum of Compound **5**

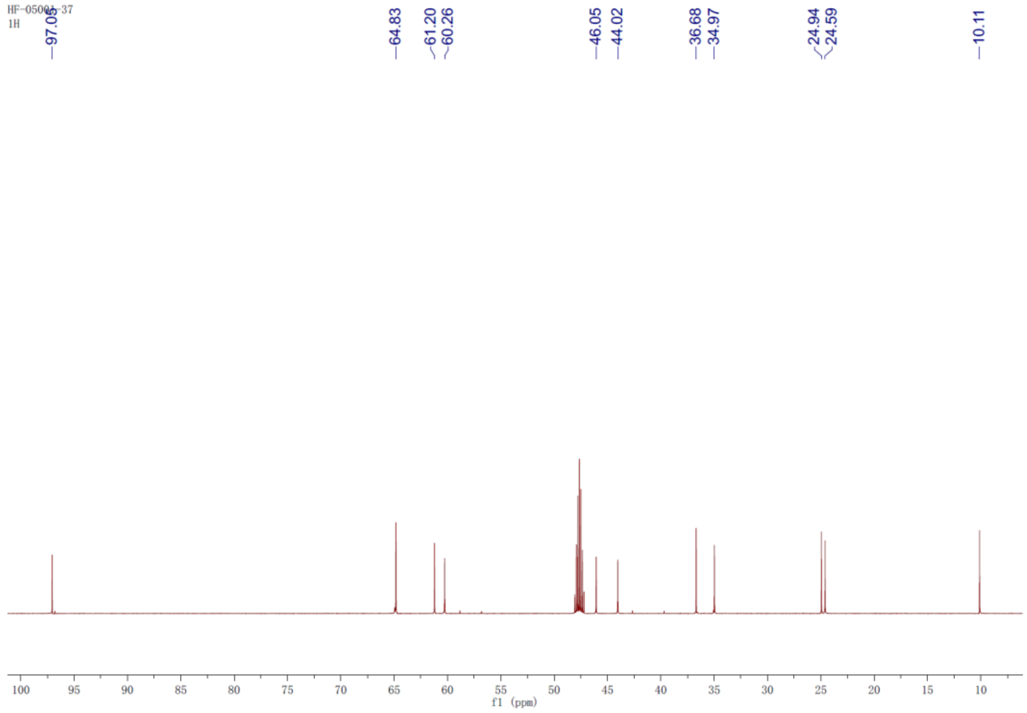

Figure S19. ^{13}C-NMR spectrum of Compound **5**

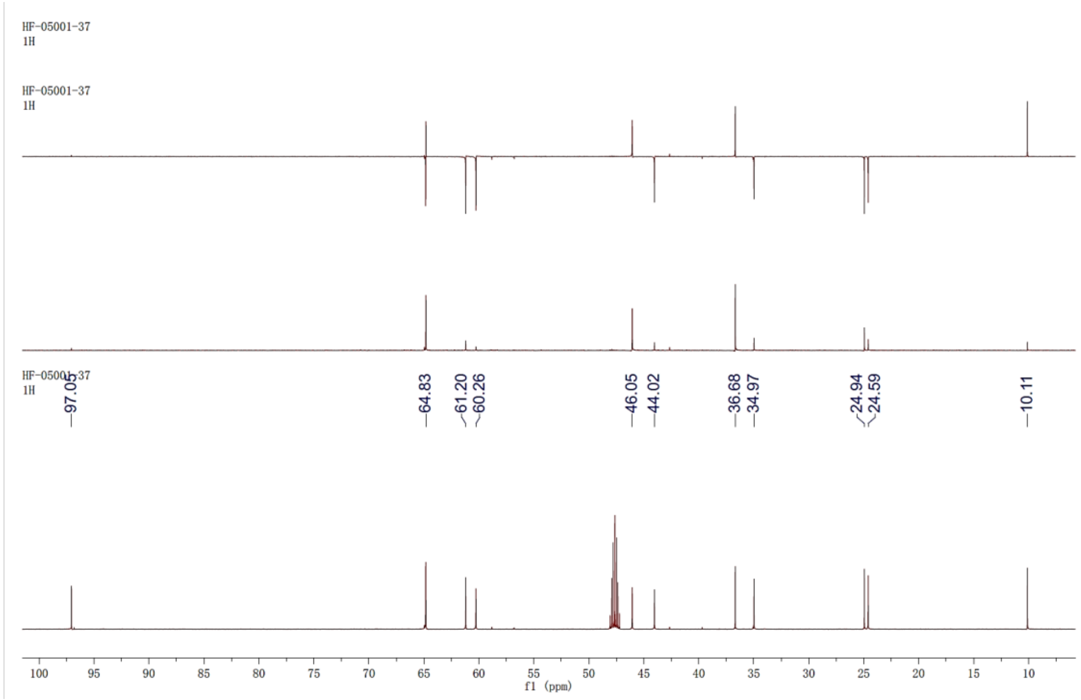

Figure S20. DEPT spectrum of Compound **5**

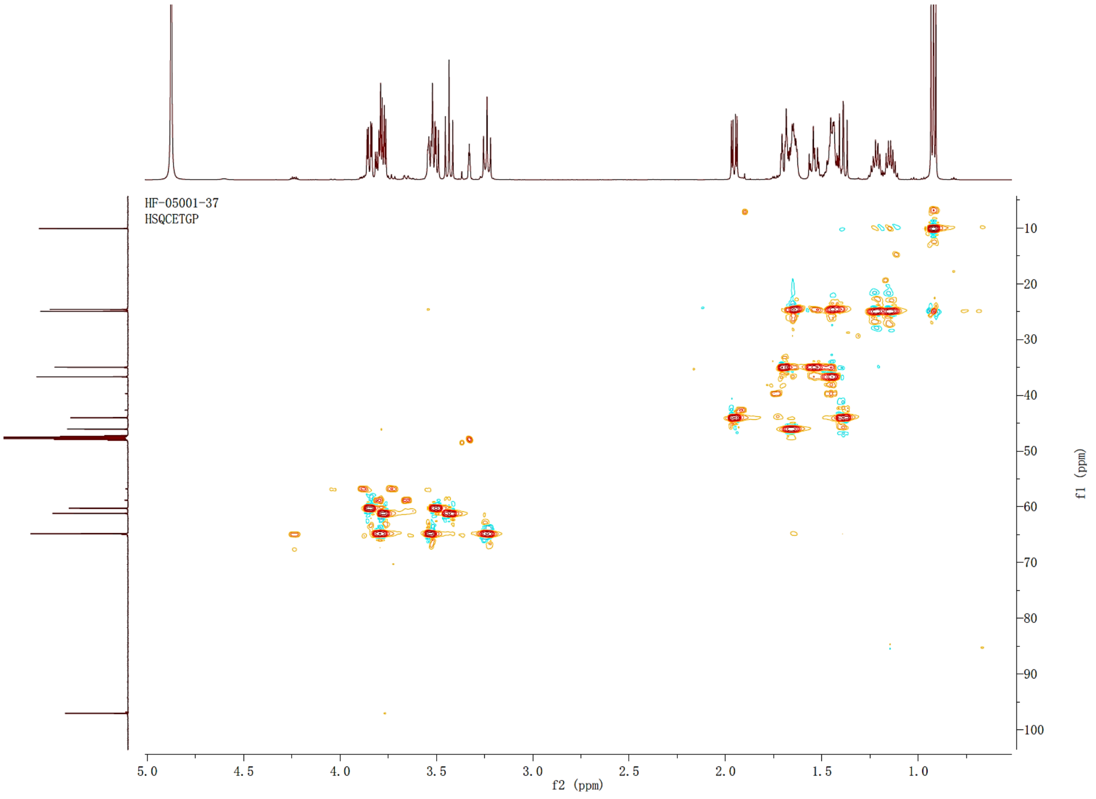

Figure S21. HSQC spectrum of Compound **5**

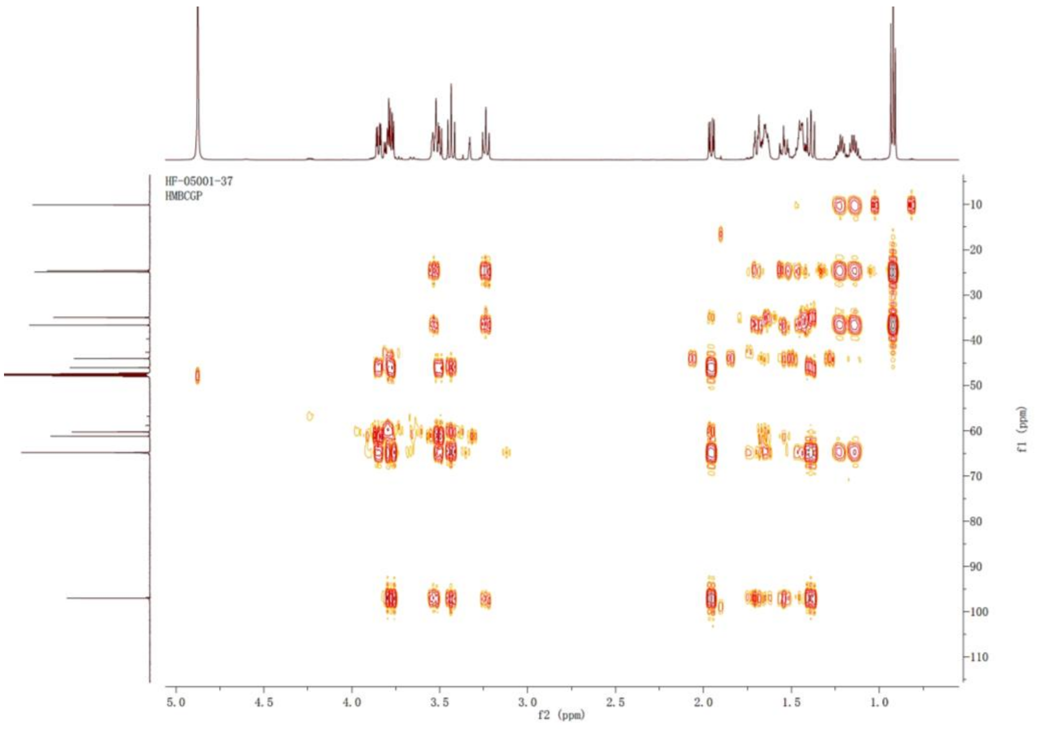

Figure S22. HMBC spectrum of Compound **5**

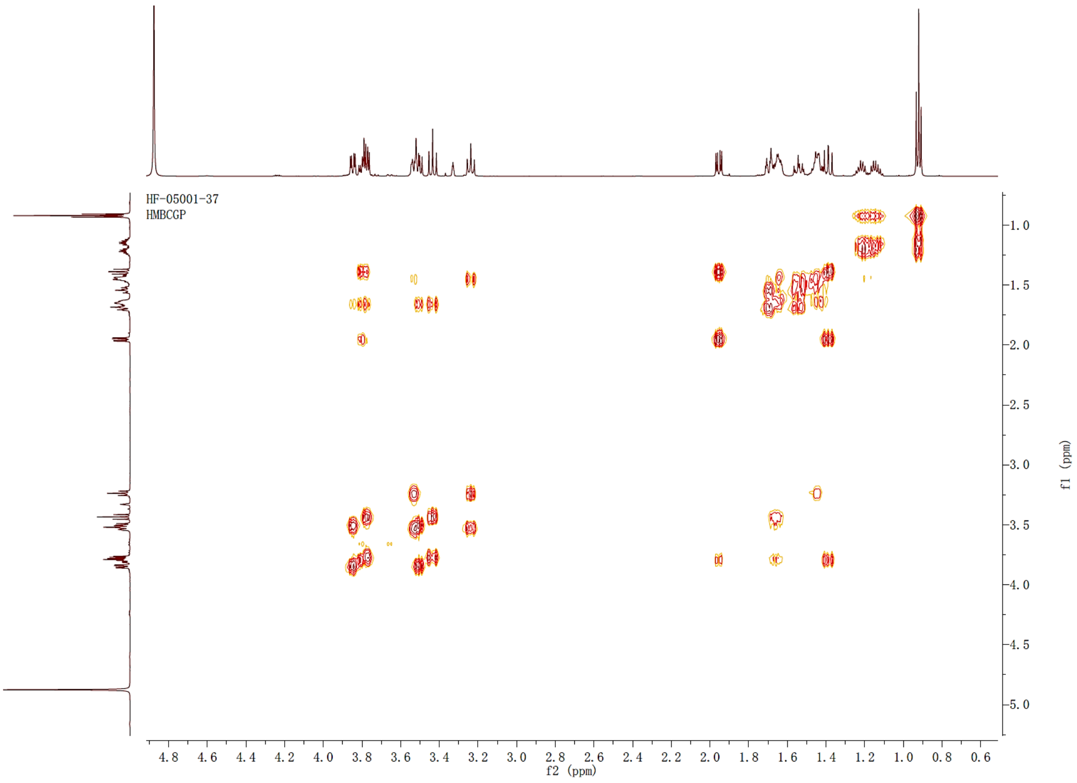

Figure S23. ^{1}H-^{1}H COSY spectrum of Compound **5**

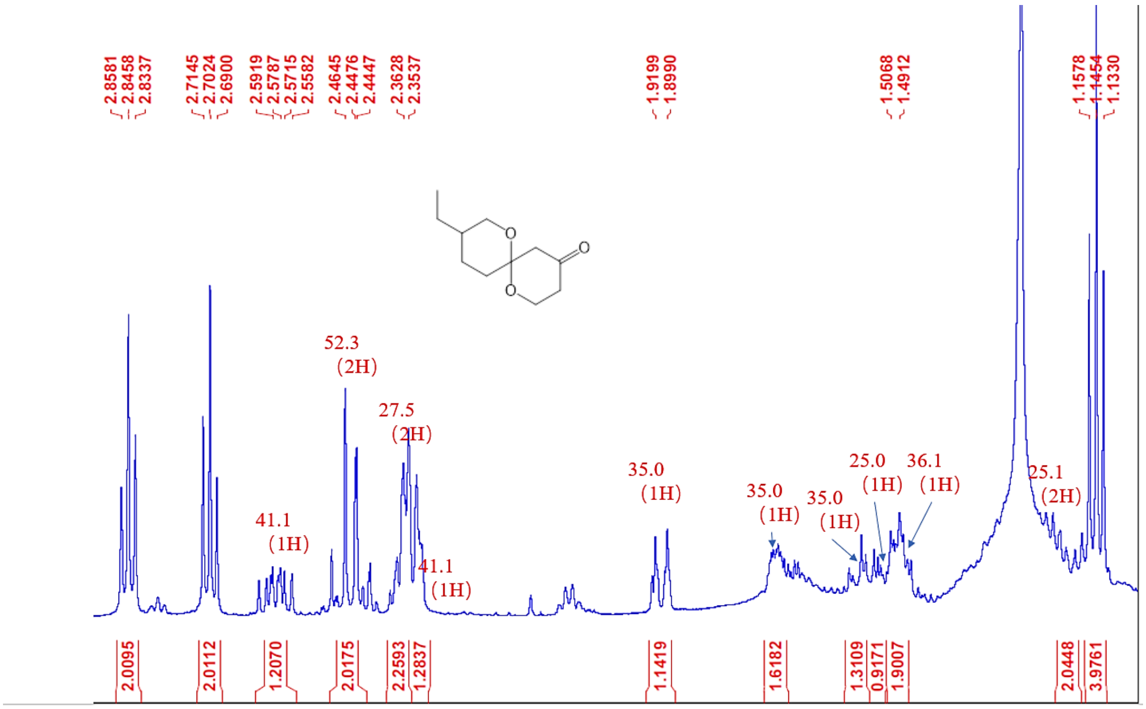

Figure S24. ¹H-NMR spectrum of Compound **6**

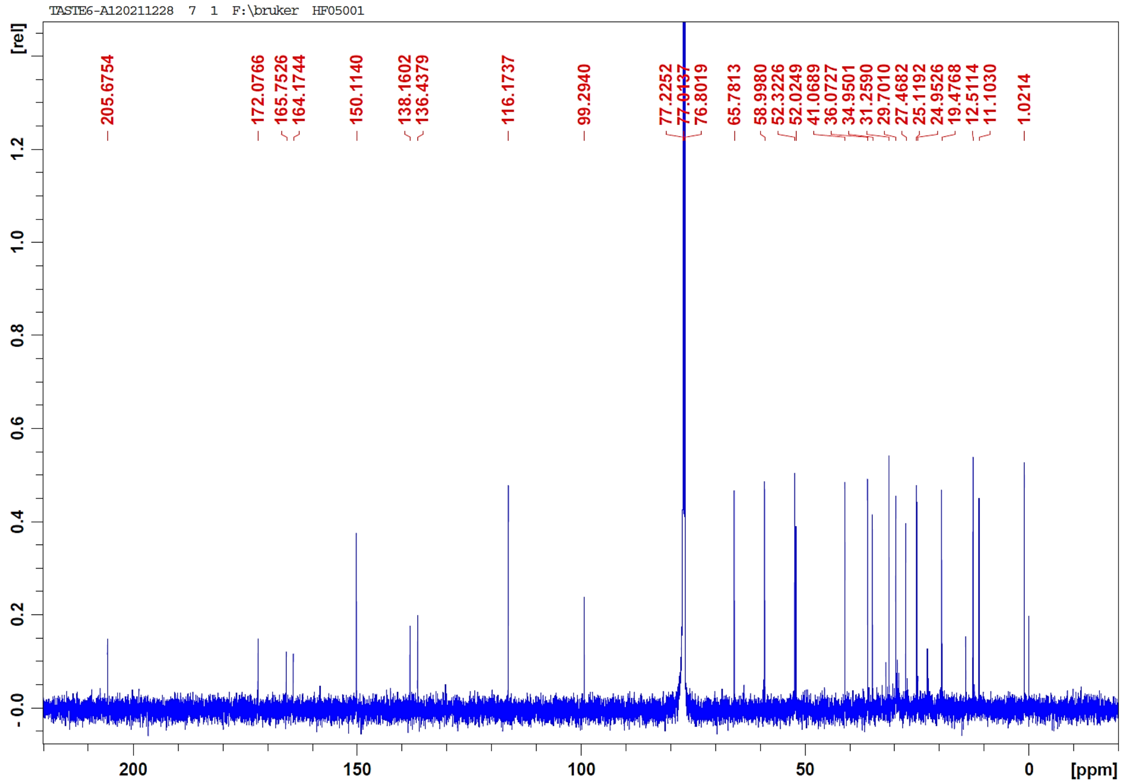

Figure S25. ¹³C-NMR spectrum of Compound **6**

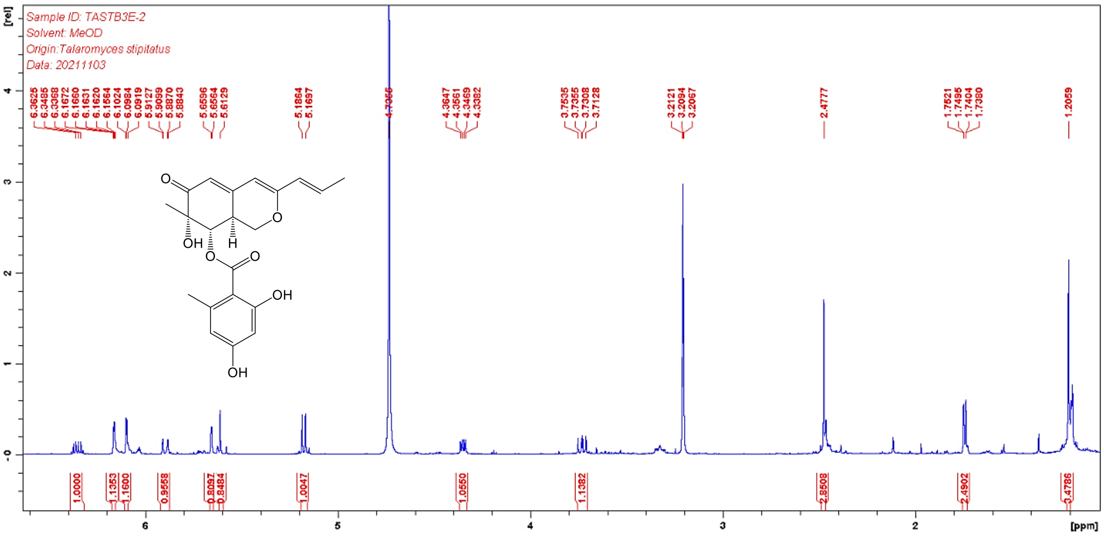

Figure S26. 1H-NMR spectrum of Compound **7**

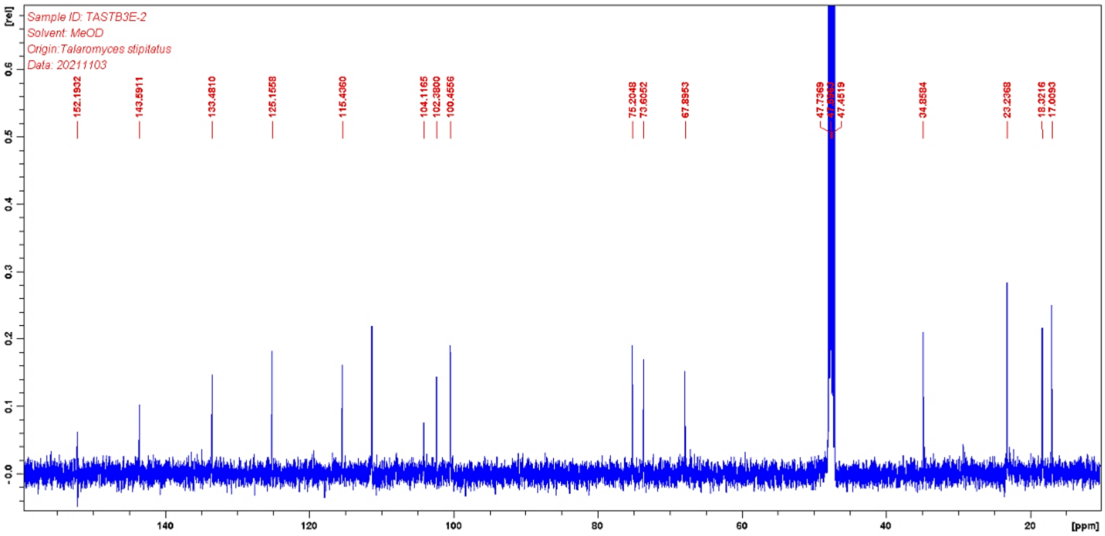

Figure S27. ^{13}C-NMR spectrum of Compound **7**

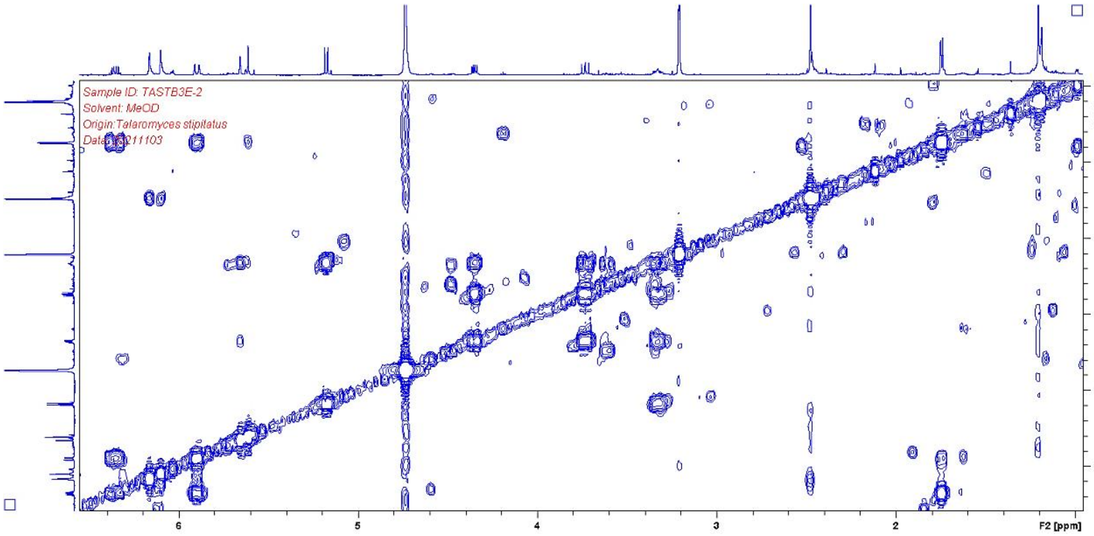

Figure S28. ^{1}H-^{1}H COSY spectrum of Compound **7**

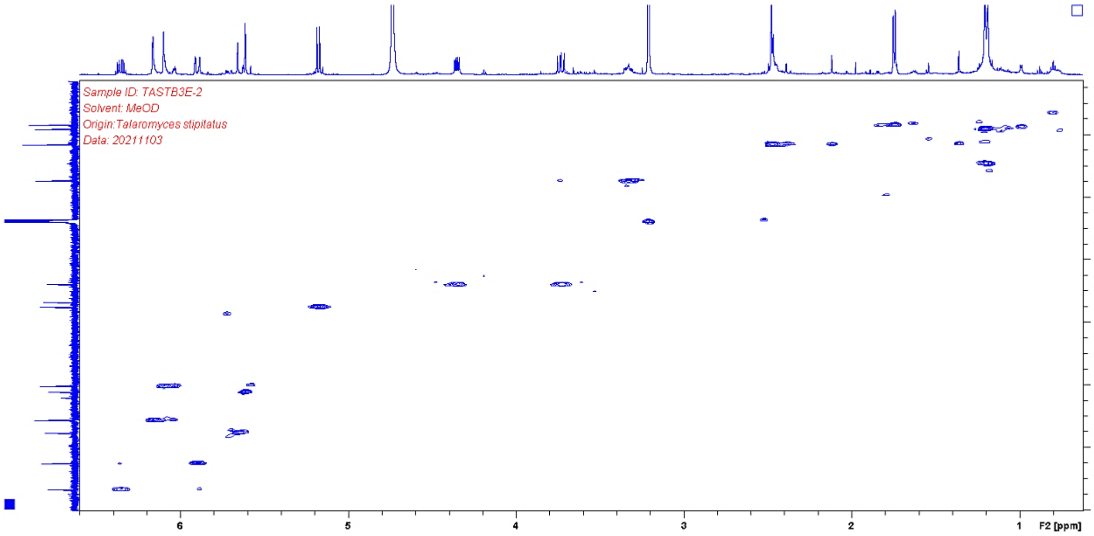

Figure S29. HSQC spectrum of Compound **7**

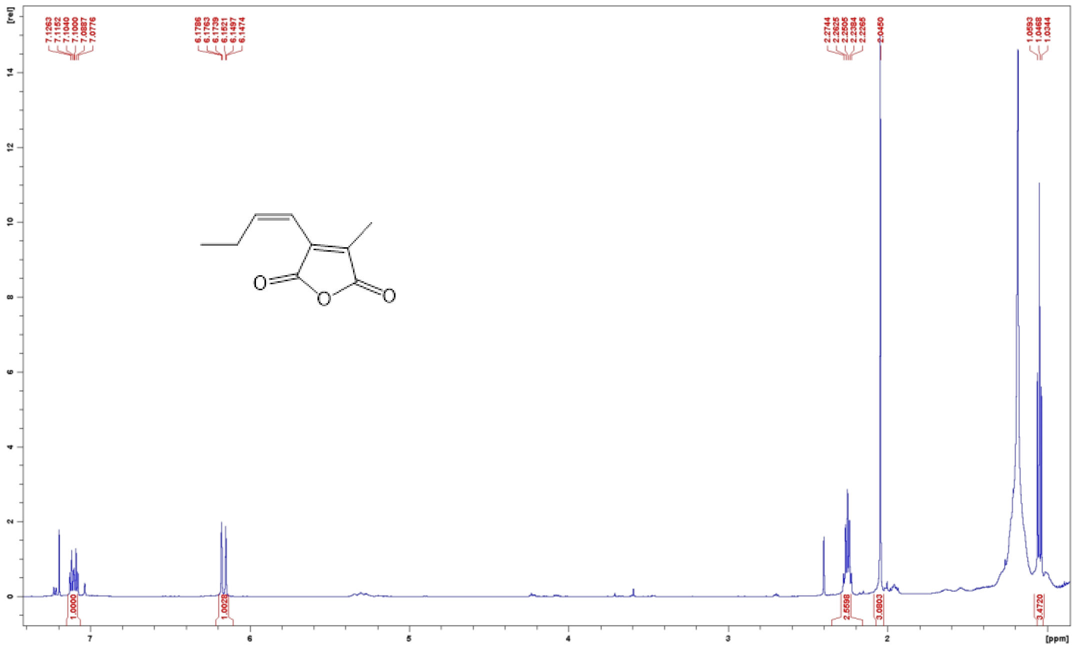

Figure S30. ^{1}H-NMR spectrum of Compound **8**

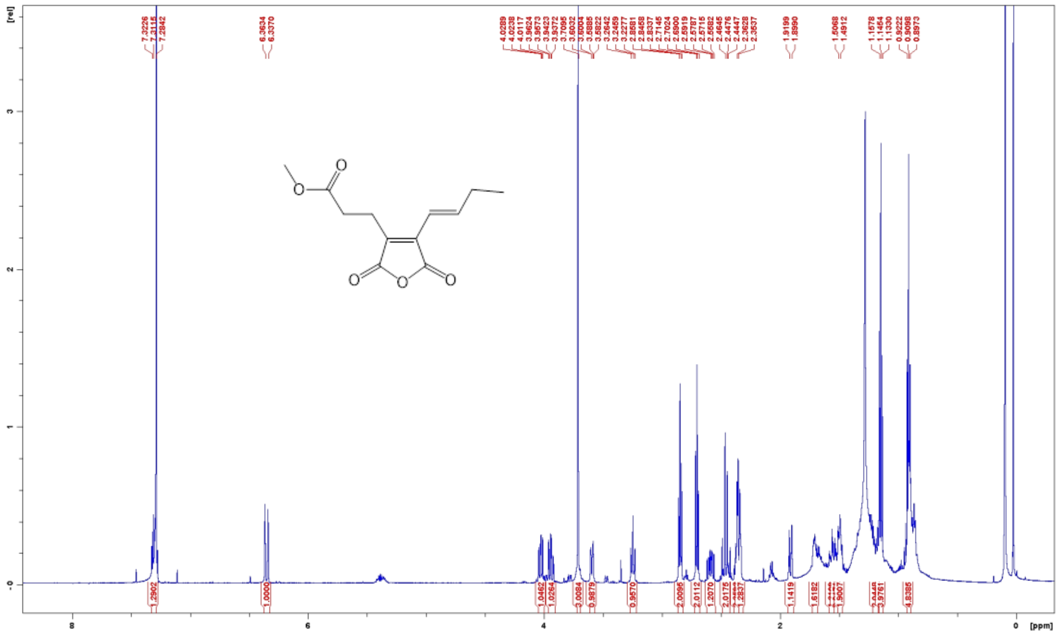

Figure S31. ^{1}H-NMR spectrum of Compound **9**

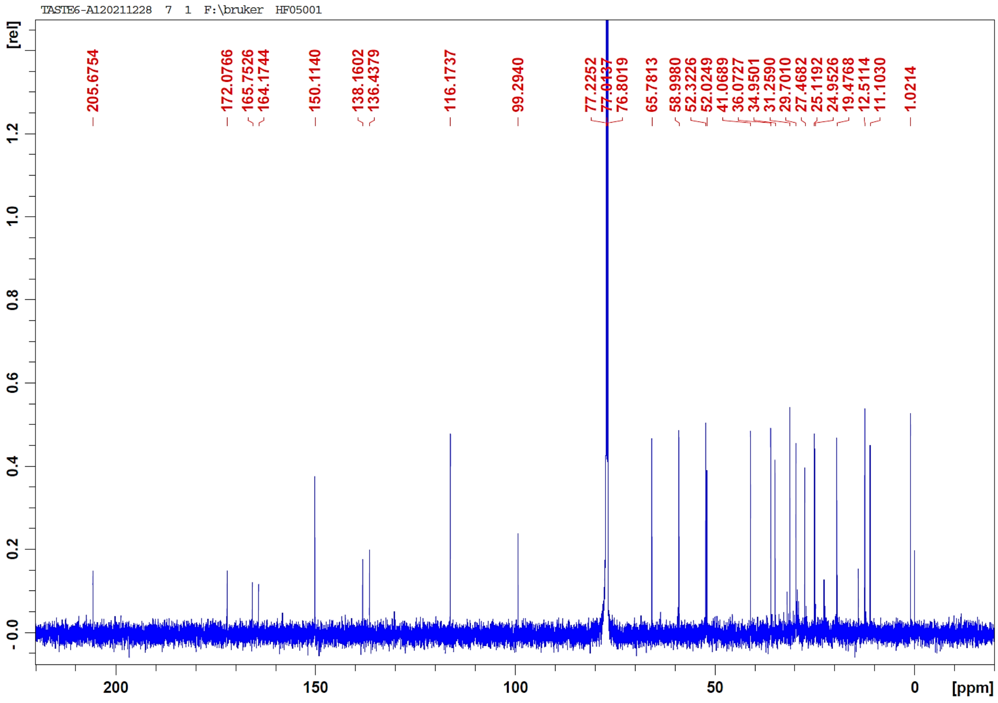

Figure S32. ^{13}C-NMR spectrum of Compound **9**

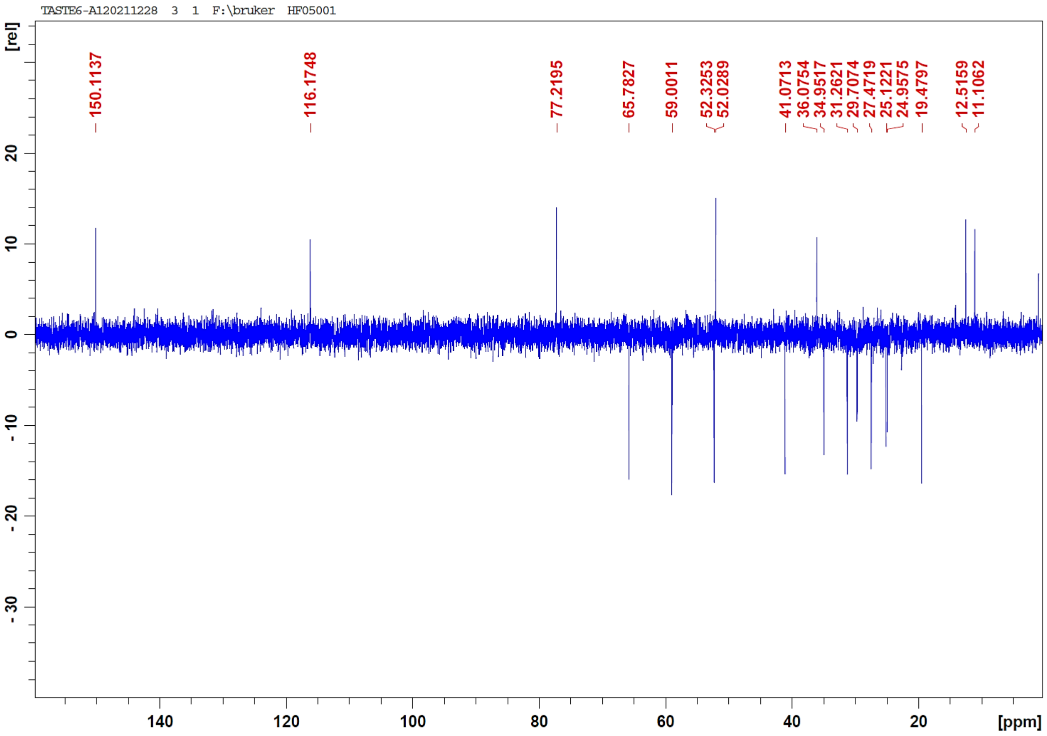

Figure S33. DEPT spectrum of Compound **9**

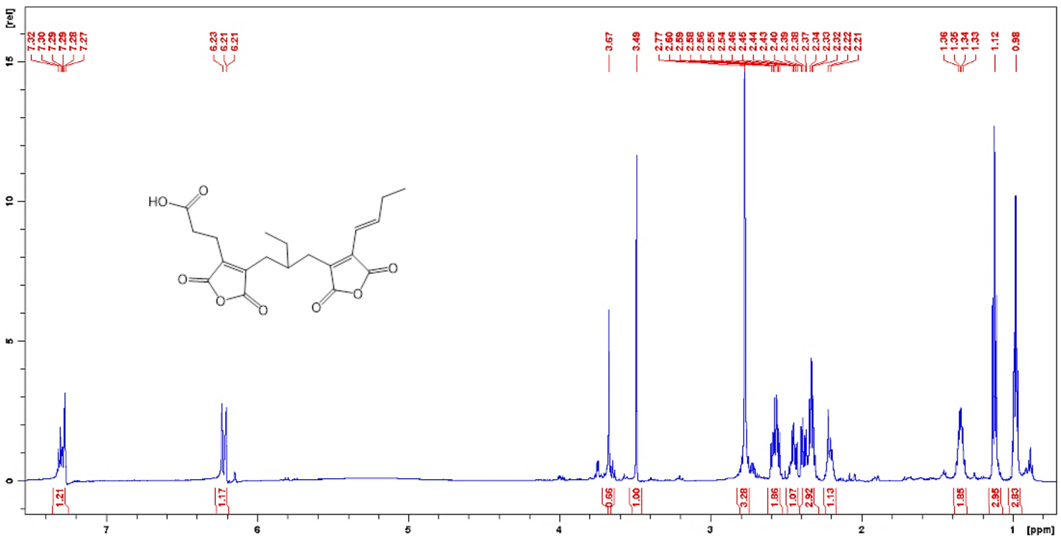

Figure S34. ^{1}H-NMR spectrum of Compound **10**

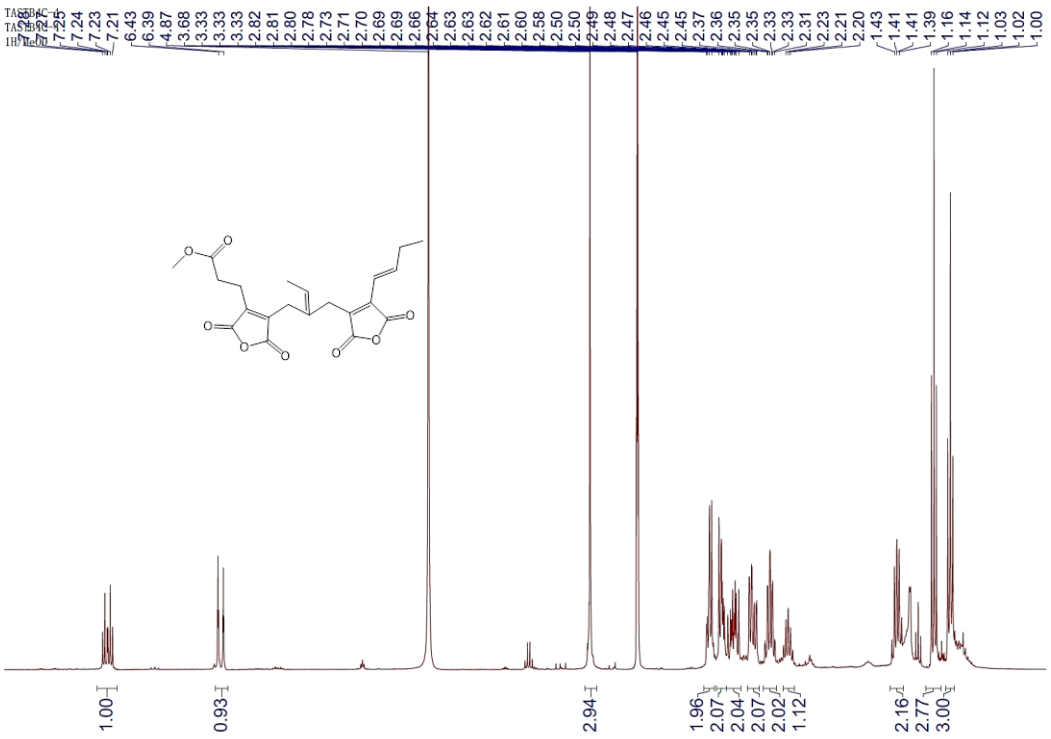

Figure S35. ^{1}H-NMR spectrum of Compound **11**

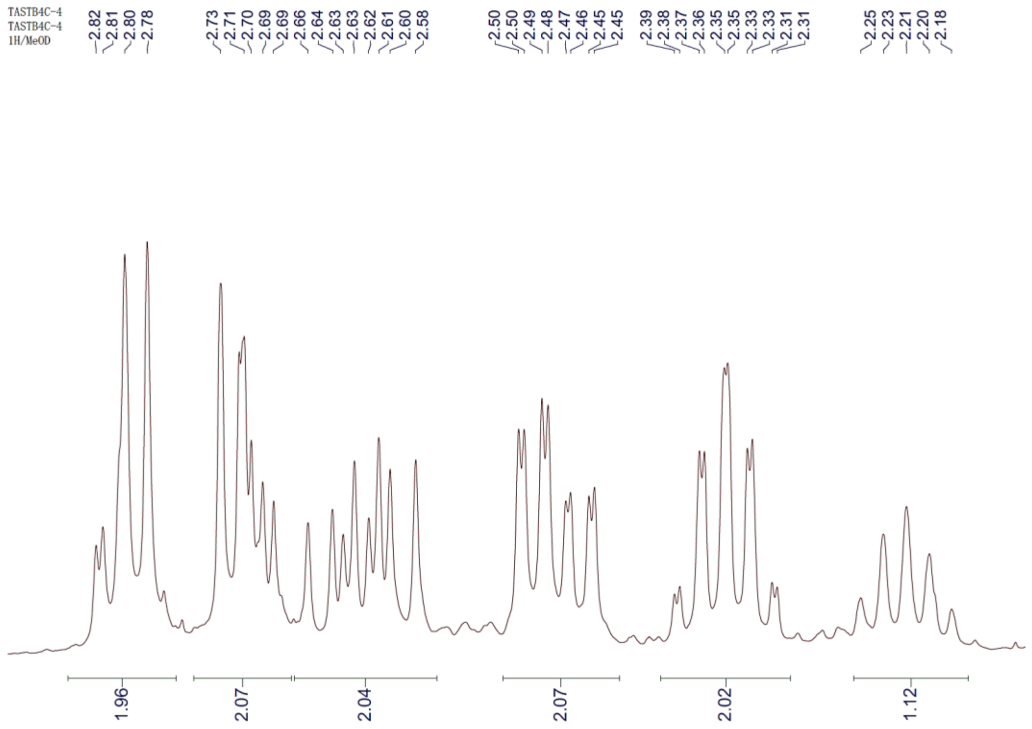

Figure S36. ^{1}H-NMR spectrum of Compound **11**

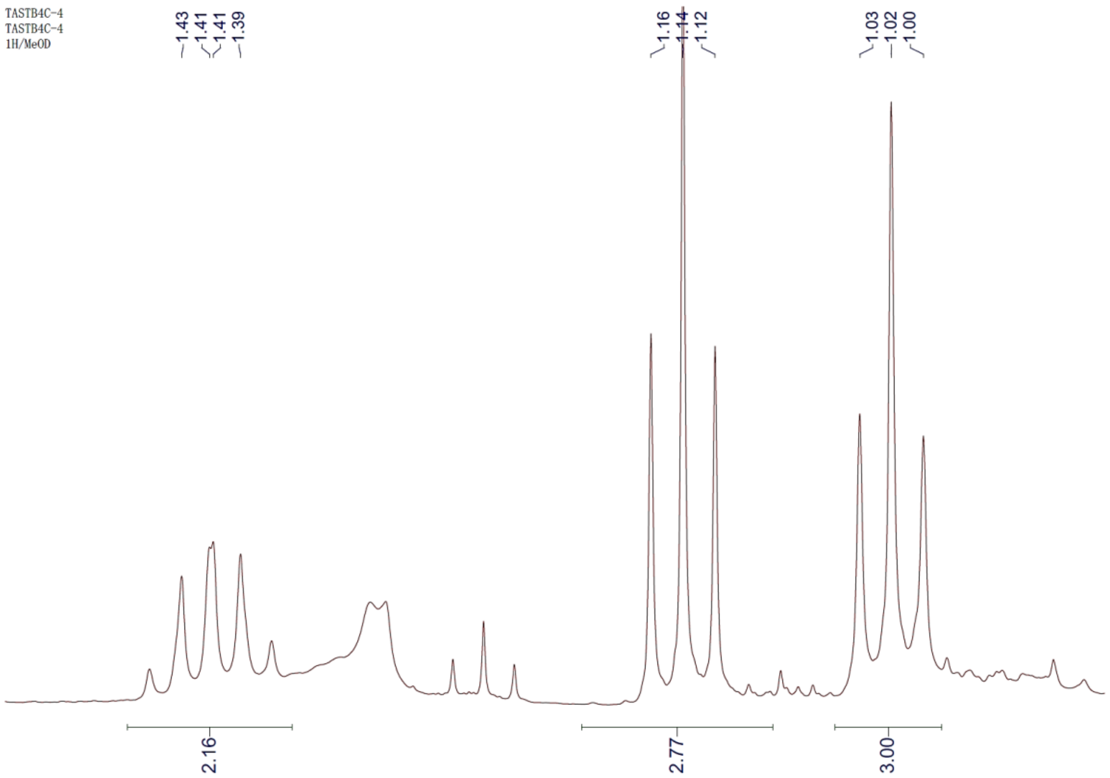

Figure S37. ^{1}H-NMR spectrum of Compound **11**

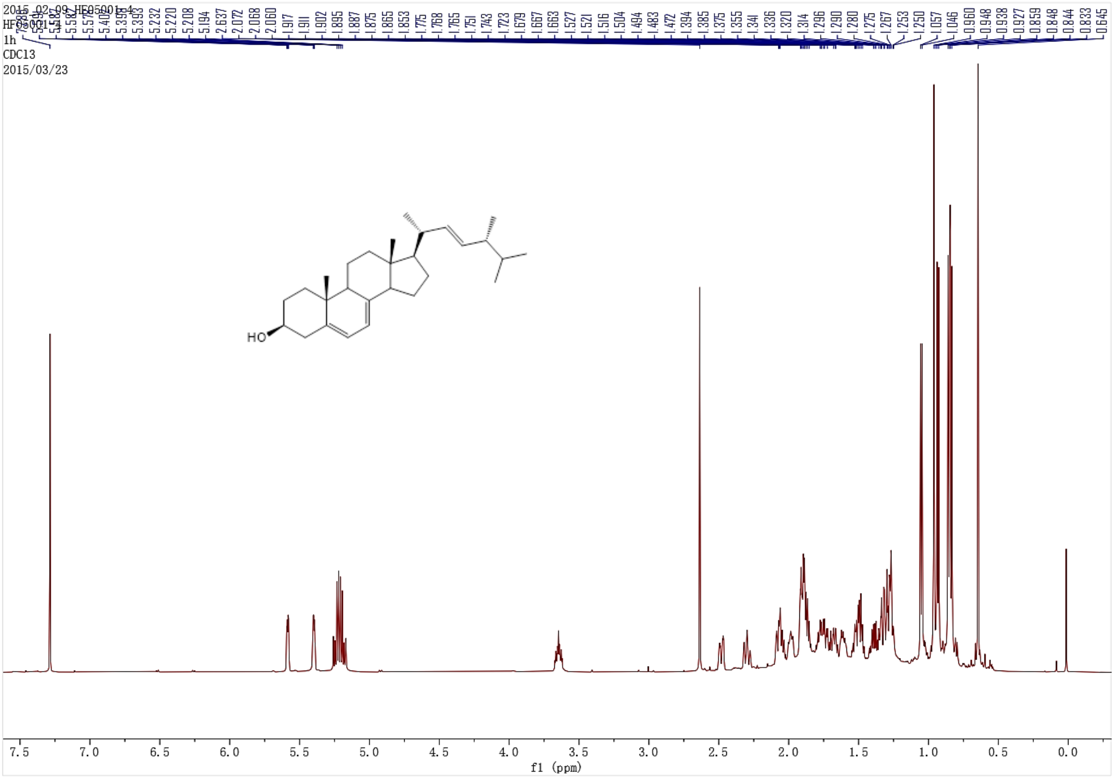

Figure S38. ^{1}H-NMR spectrum of Compound **12**

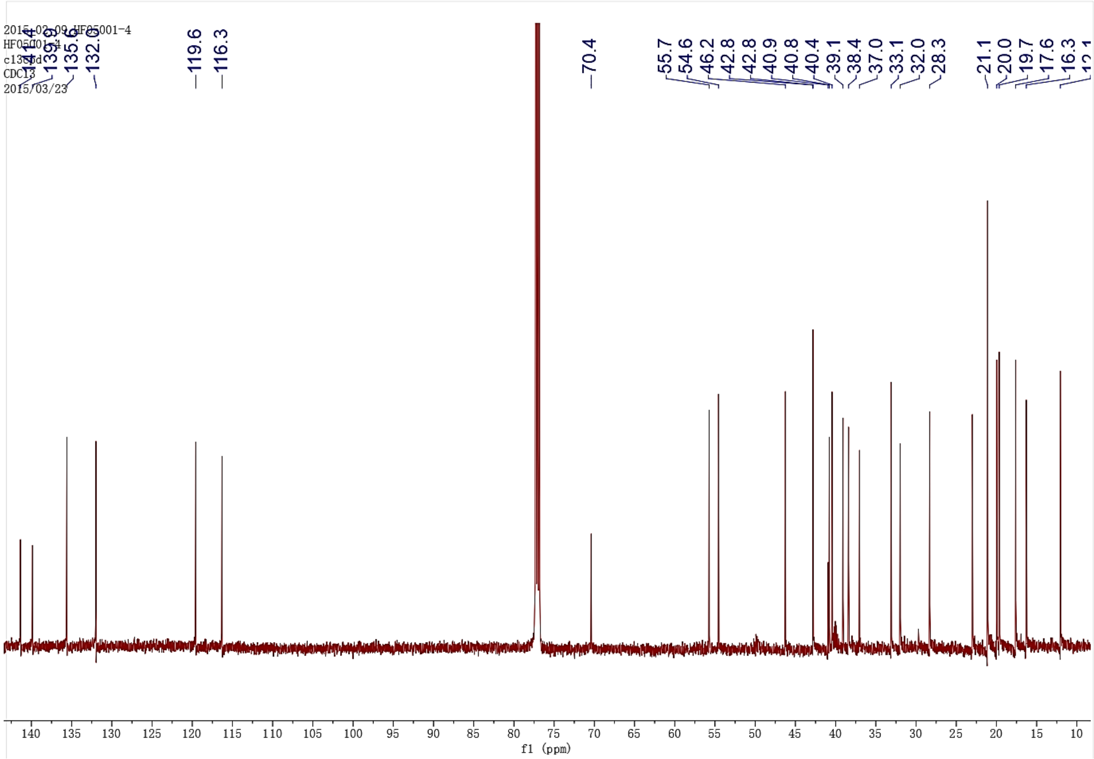

Figure S39. ^{13}C-NMR spectrum of Compound **12**

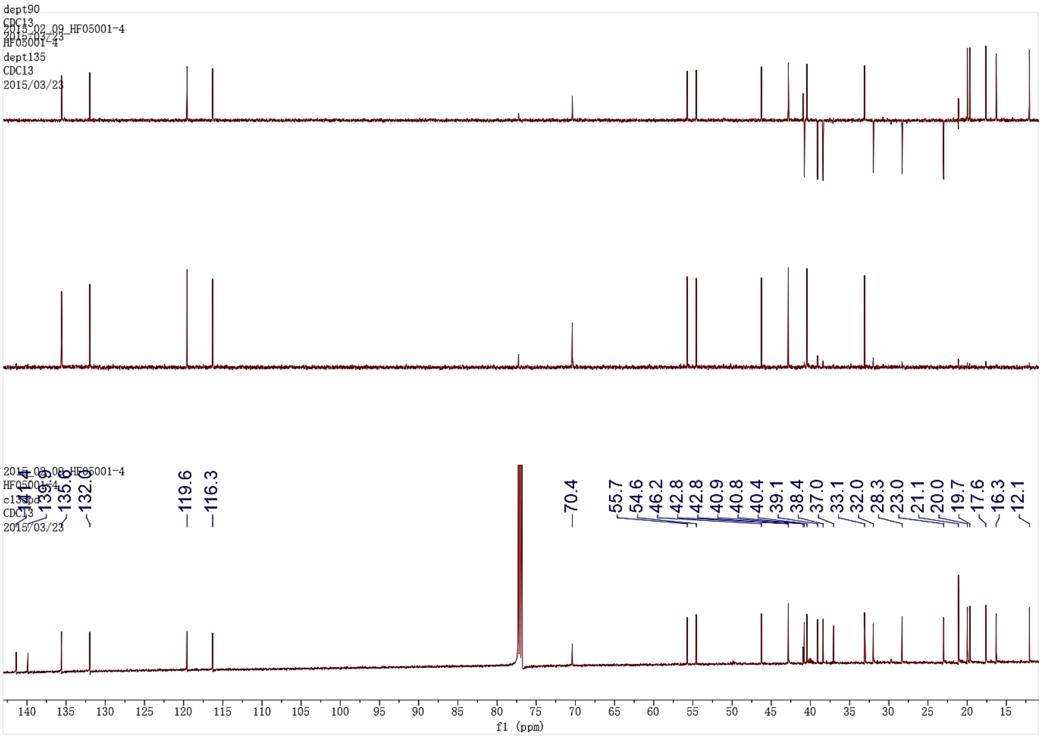

Figure S40. DEPT spectrum of Compound **12**

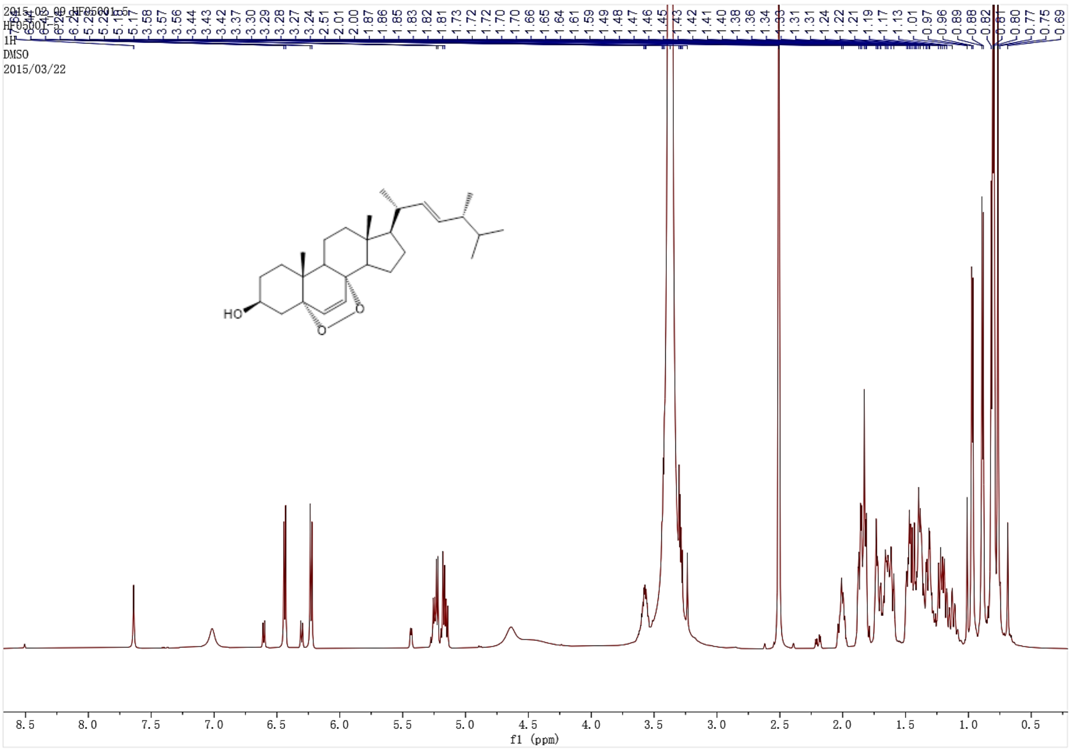

Figure S41. ^{1}H-NMR spectrum of Compound **13**

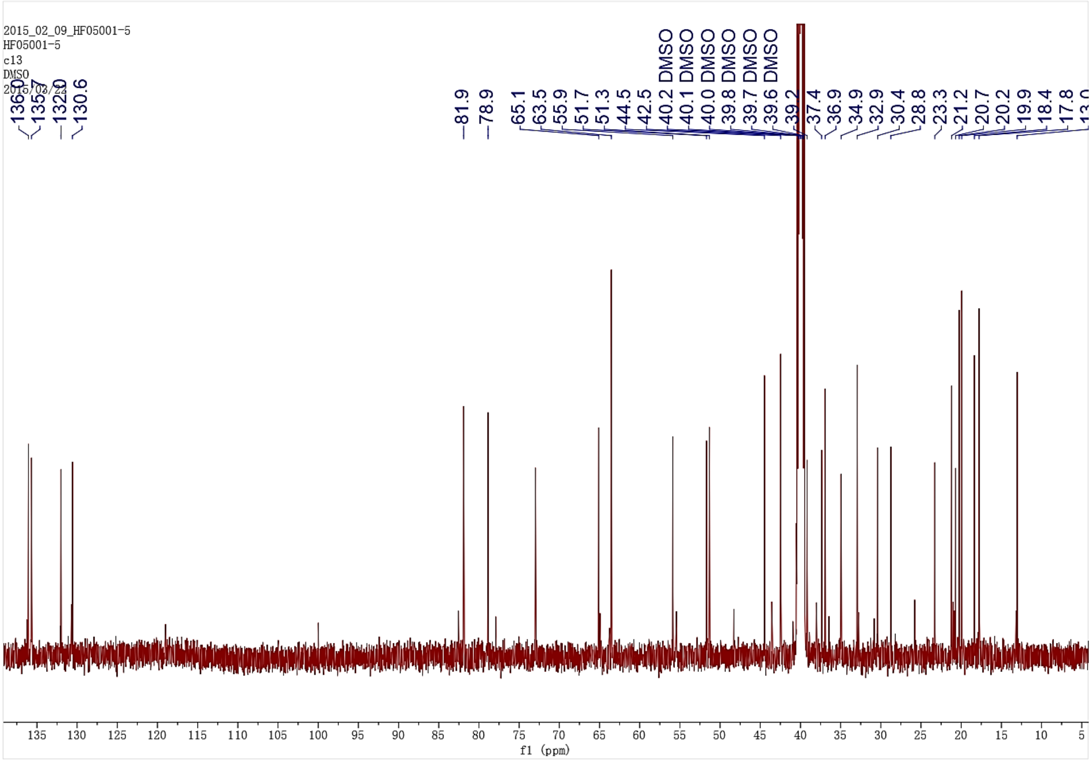

Figure S42. ^{13}C-NMR spectrum of Compound **13**

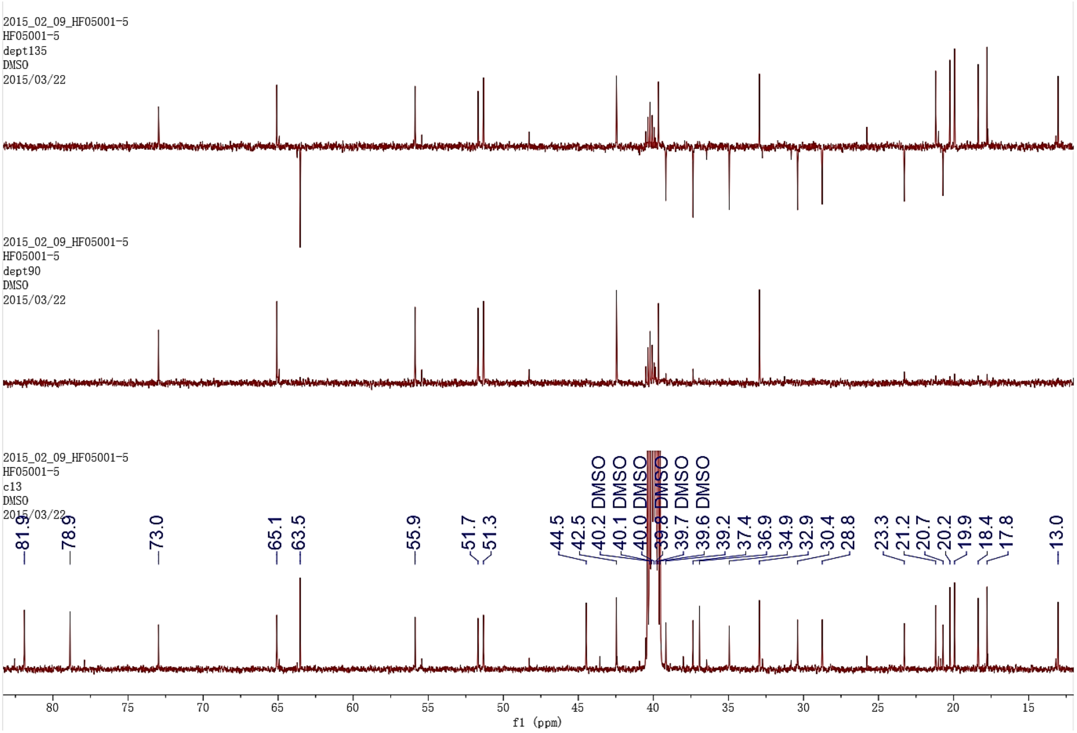

Figure S43. DEPT spectrum of Compound **13**

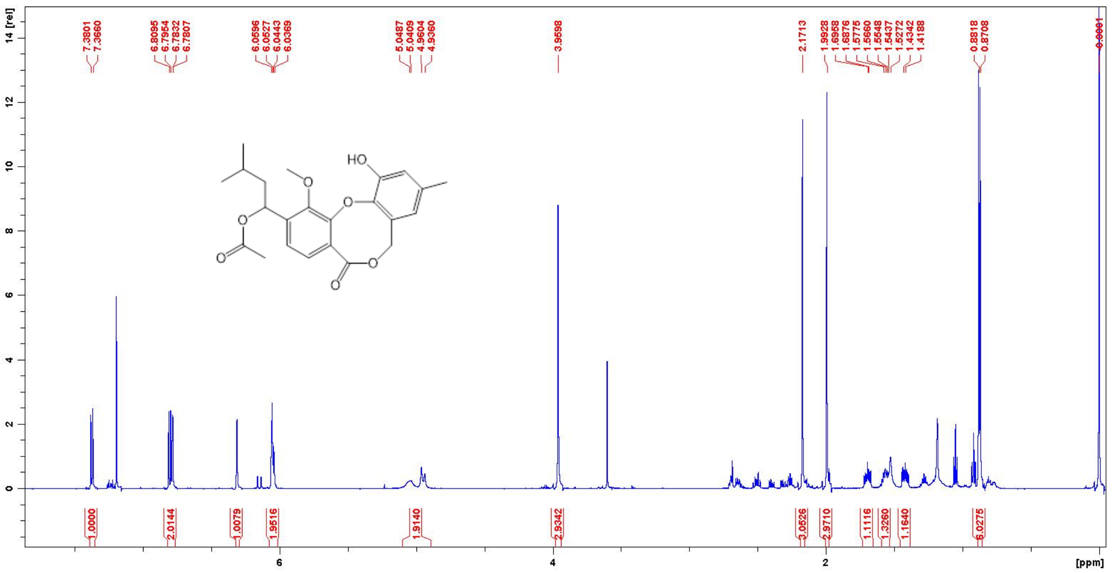

Figure S44. ^{1}H-NMR spectrum of Compound **14**

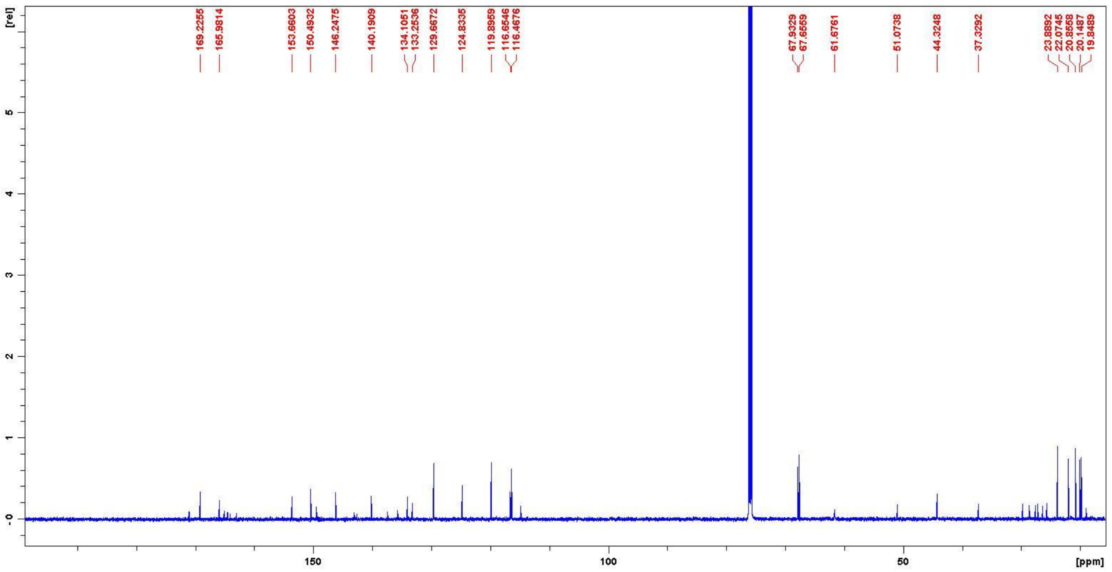

Figure S45. ^{13}C-NMR spectrum of Compound **14**

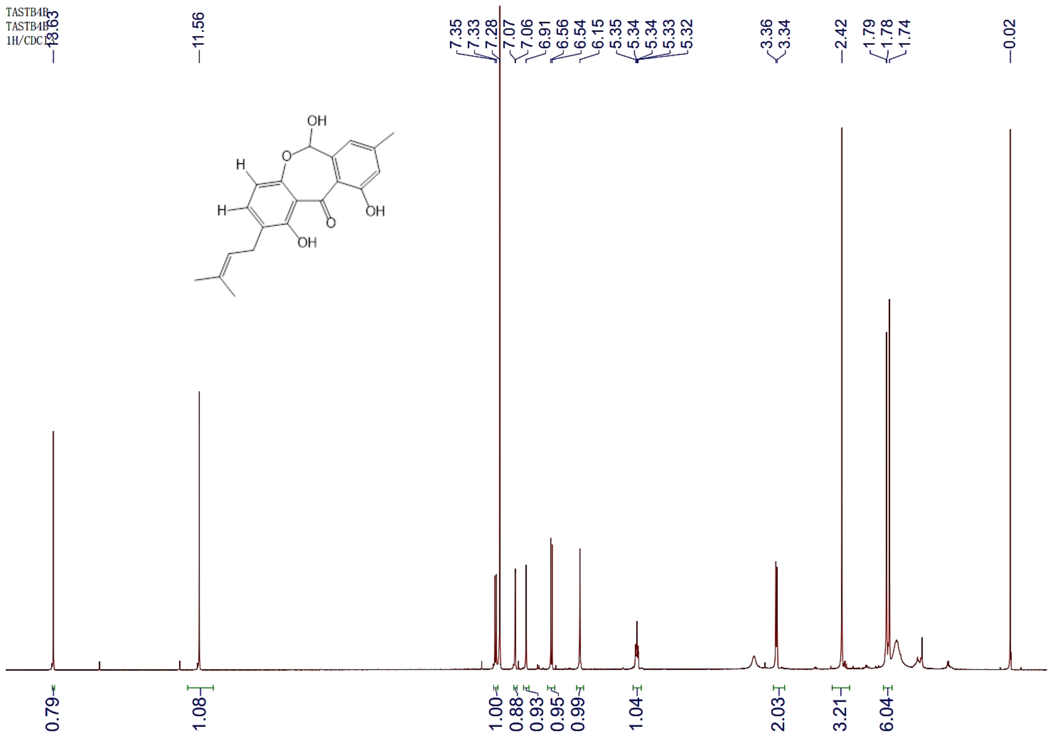

Figure S46. ^{1}H-NMR spectrum of Compound **15**

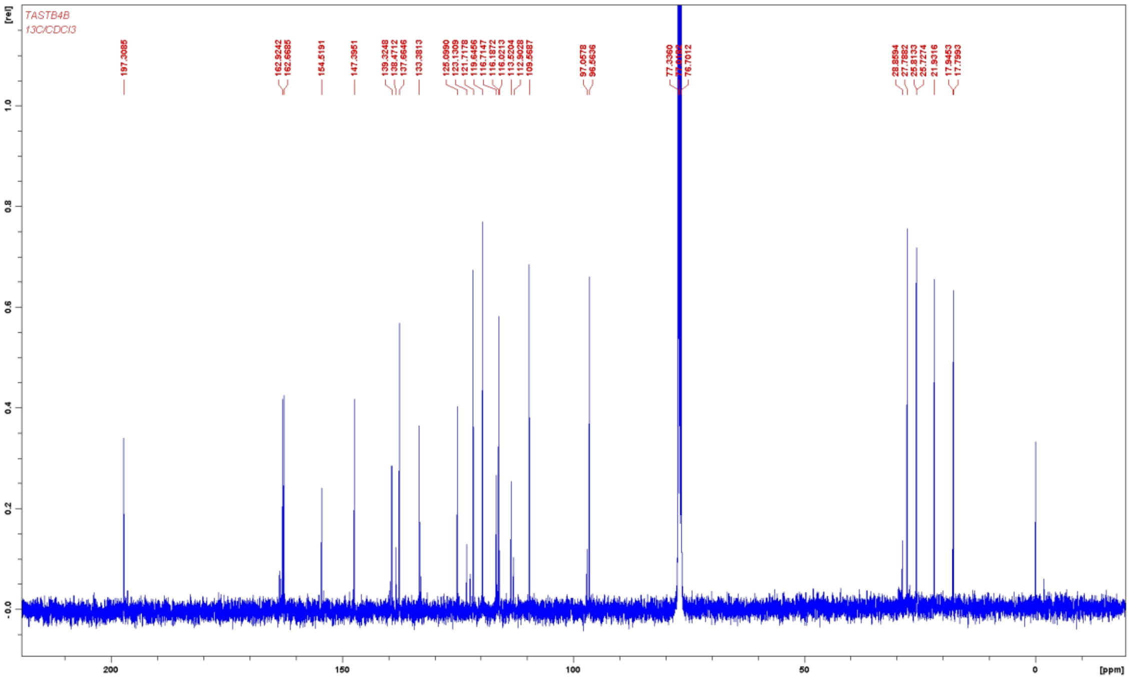

Figure S47. ^{13}C-NMR spectrum of Compound **15**

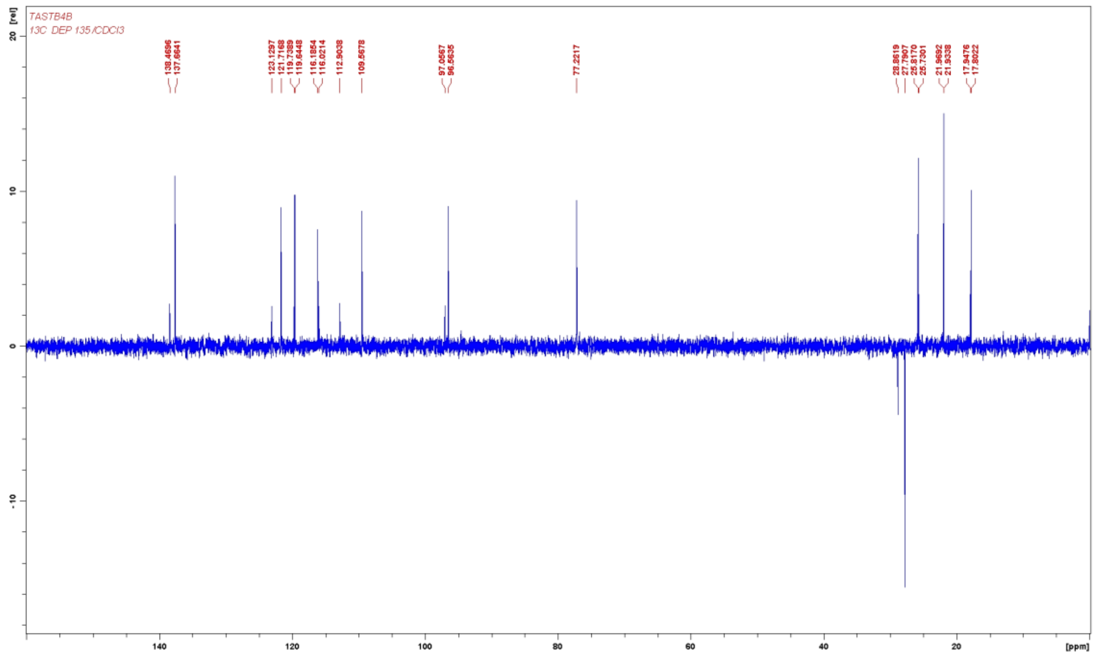

Figure S48. DEPT spectrum of Compound **15**

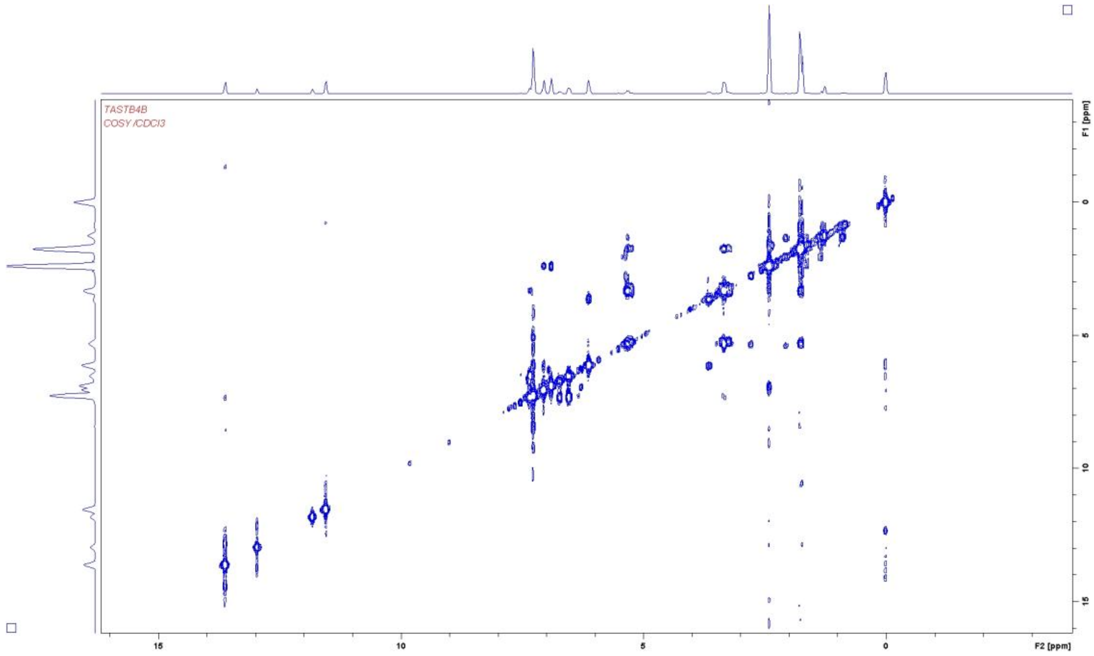

Figure S49. ^{1}H-^{1}H COSY spectrum of Compound **15**

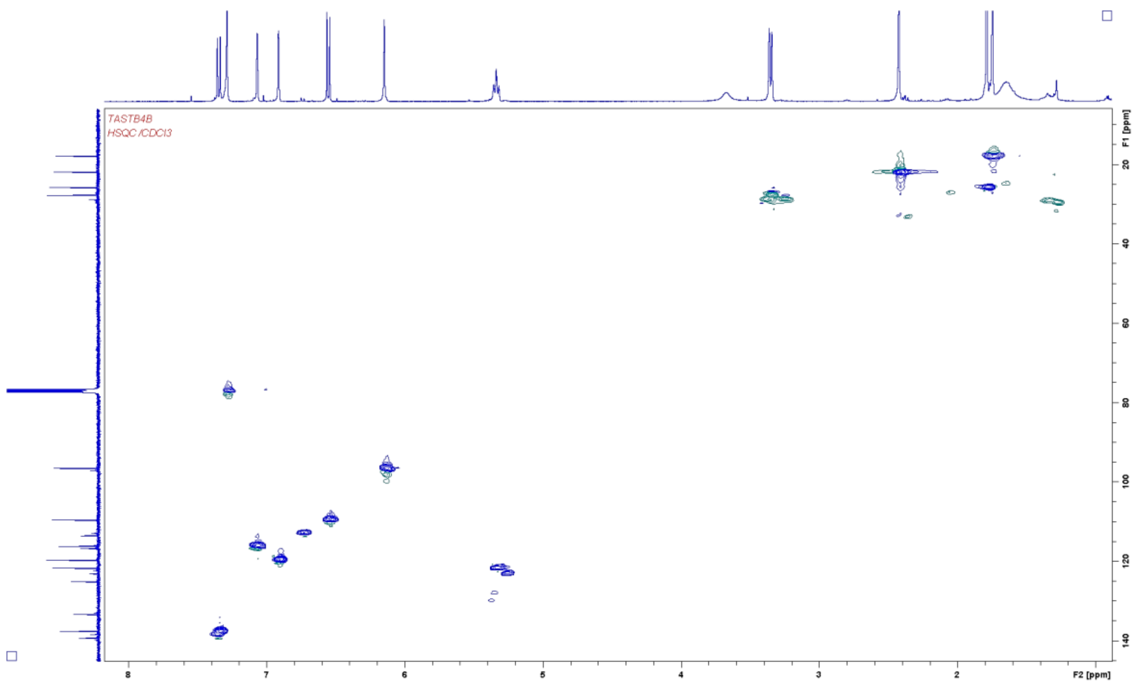

Figure S50. HSQC spectrum of Compound **15**

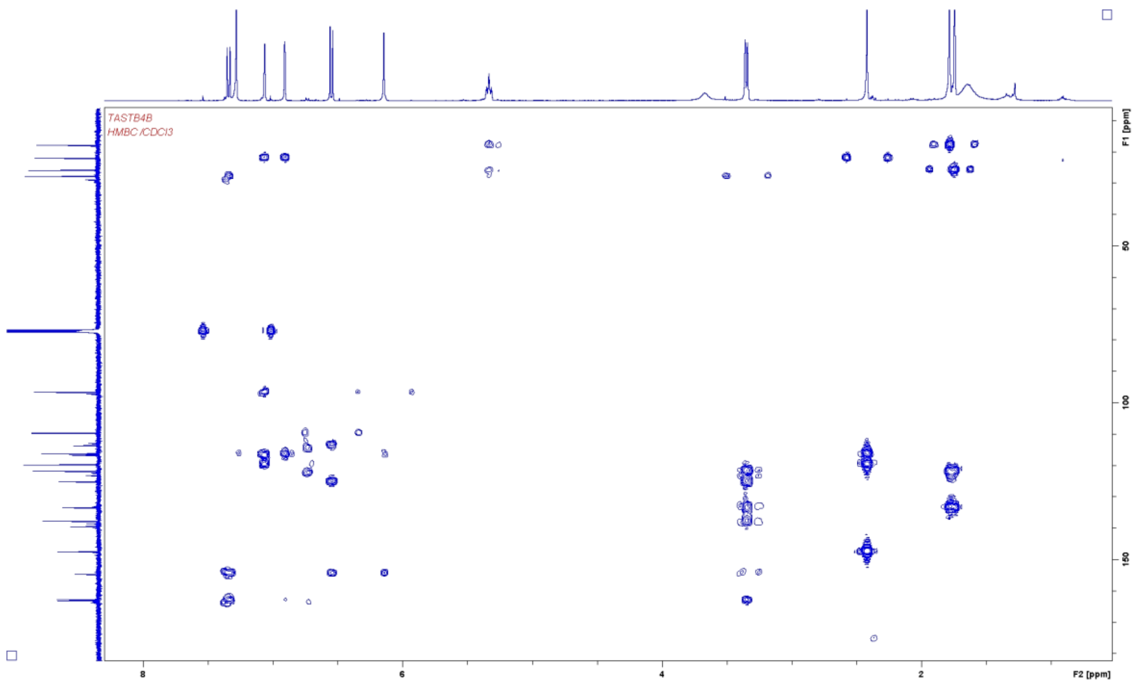

Figure S51. HMBC spectrum of Compound **15**

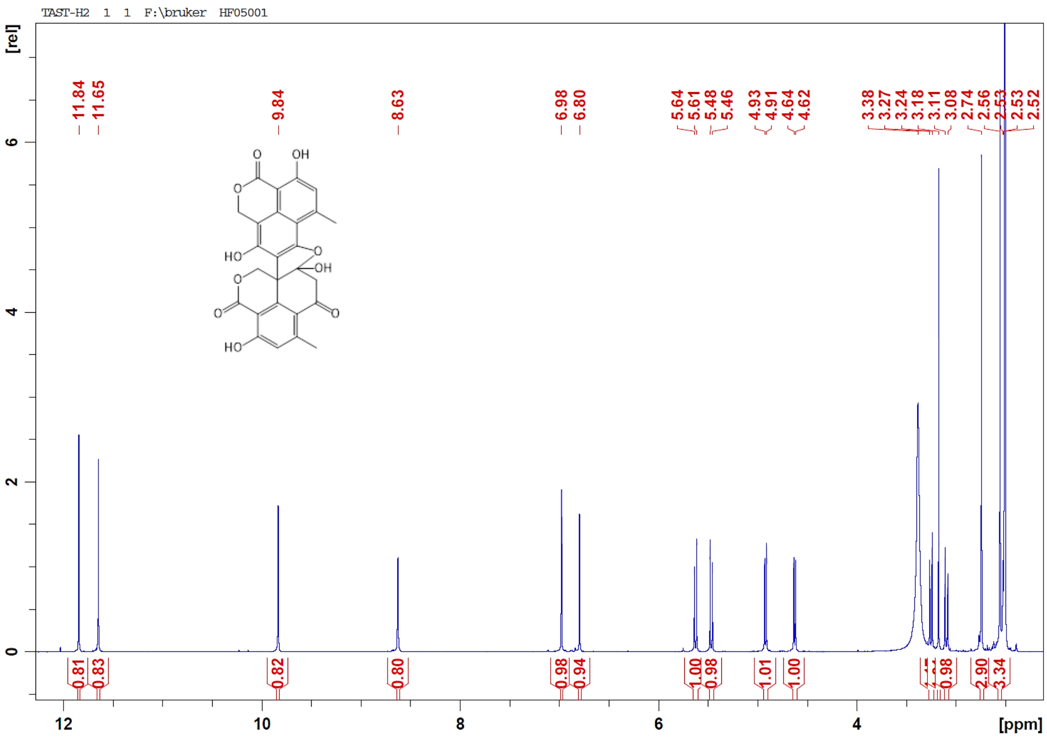

Figure S52. ¹H-NMR spectrum of Compound **16**

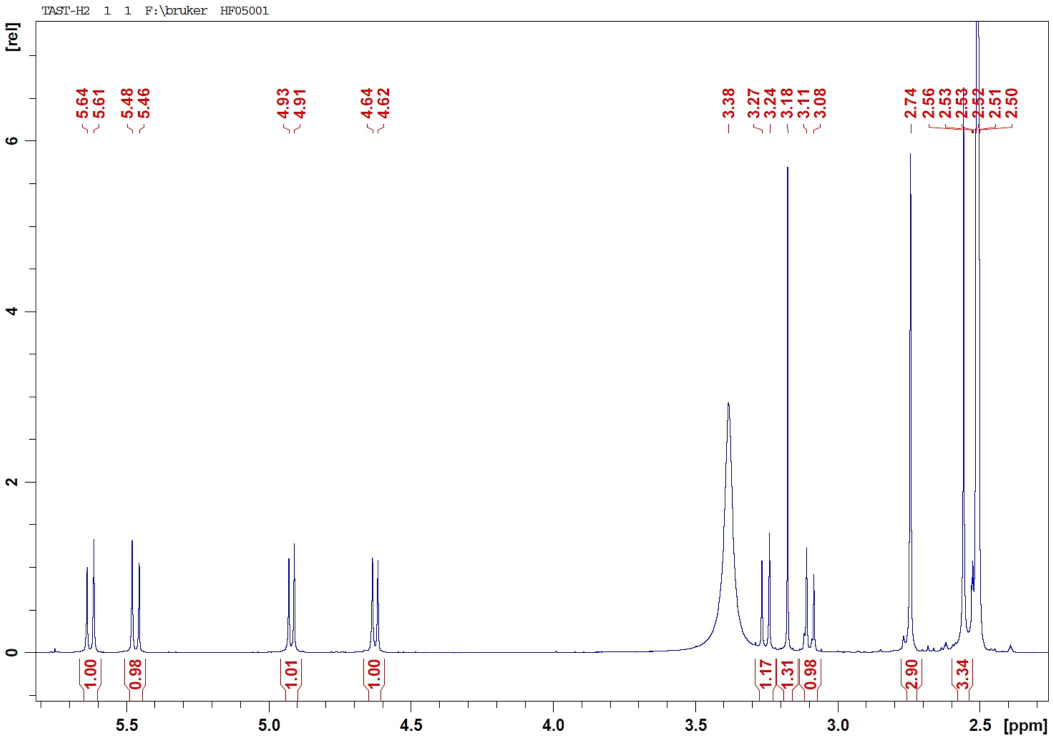

Figure S53. 1H-NMR spectrum of Compound **16**

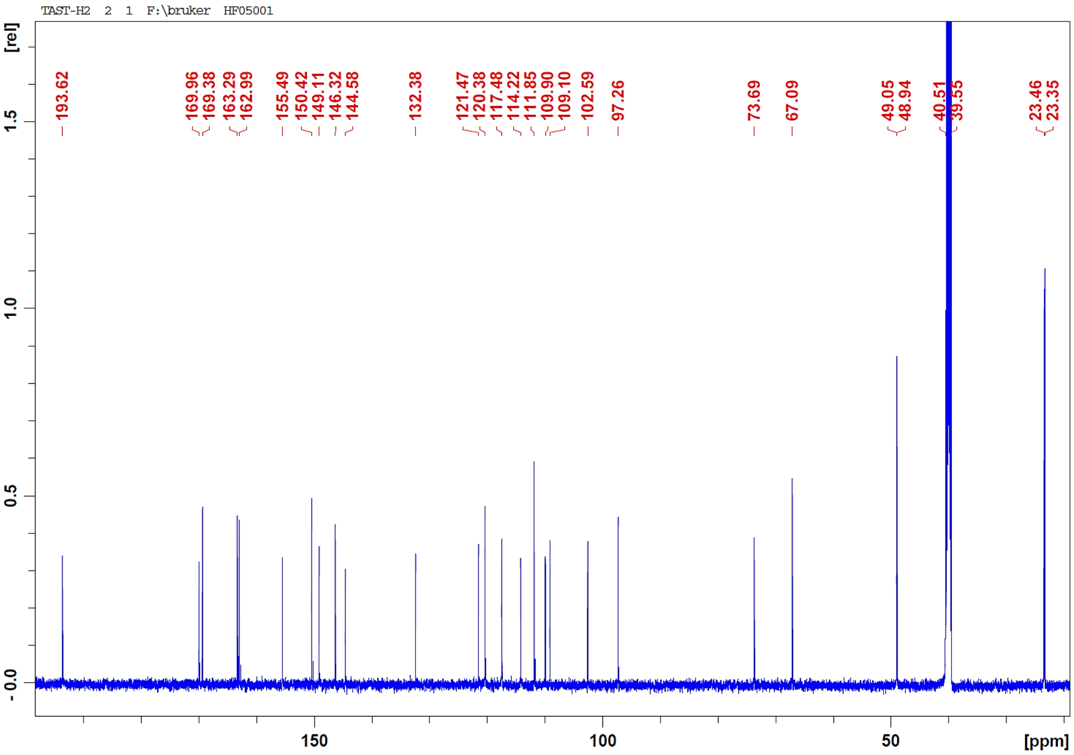

Figure S54. ^{13}C-NMR spectrum of Compound **16**

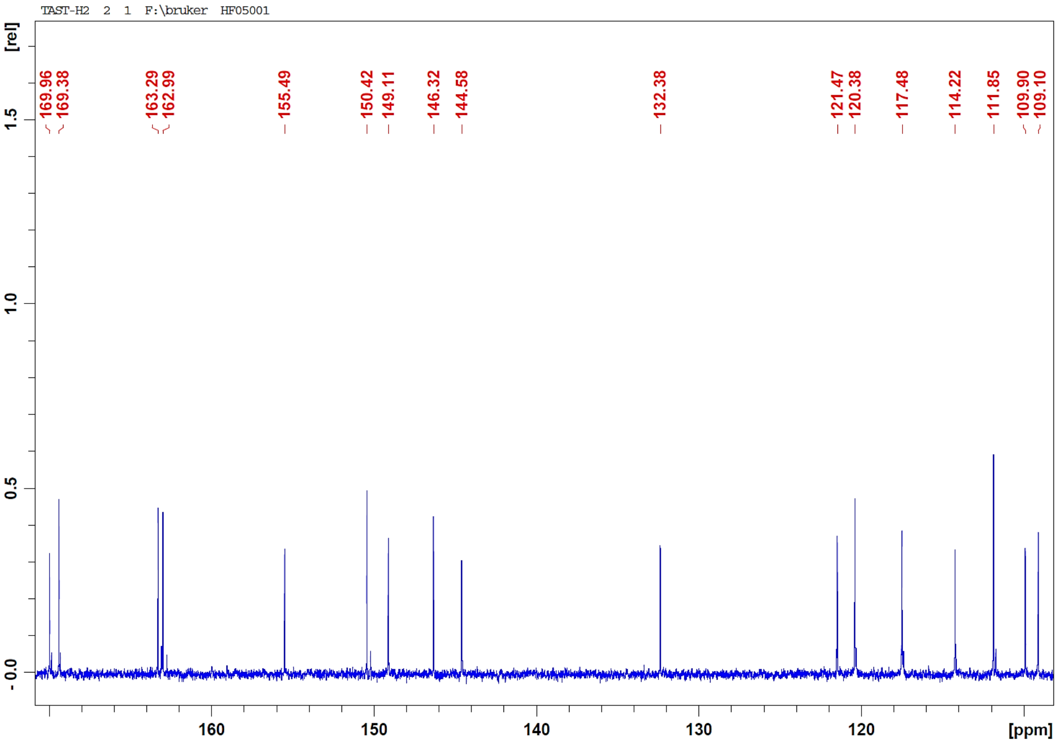

Figure S55. ^{13}C-NMR spectrum of Compound **16**

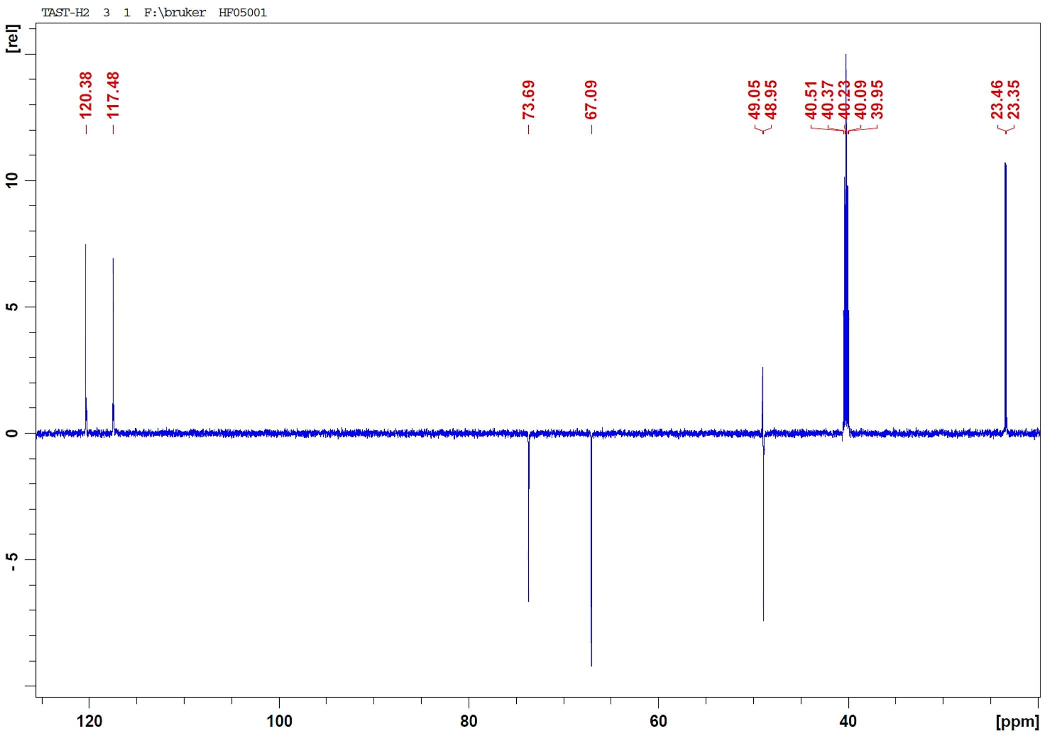

Figure S56. DEPT spectrum of Compound **16**

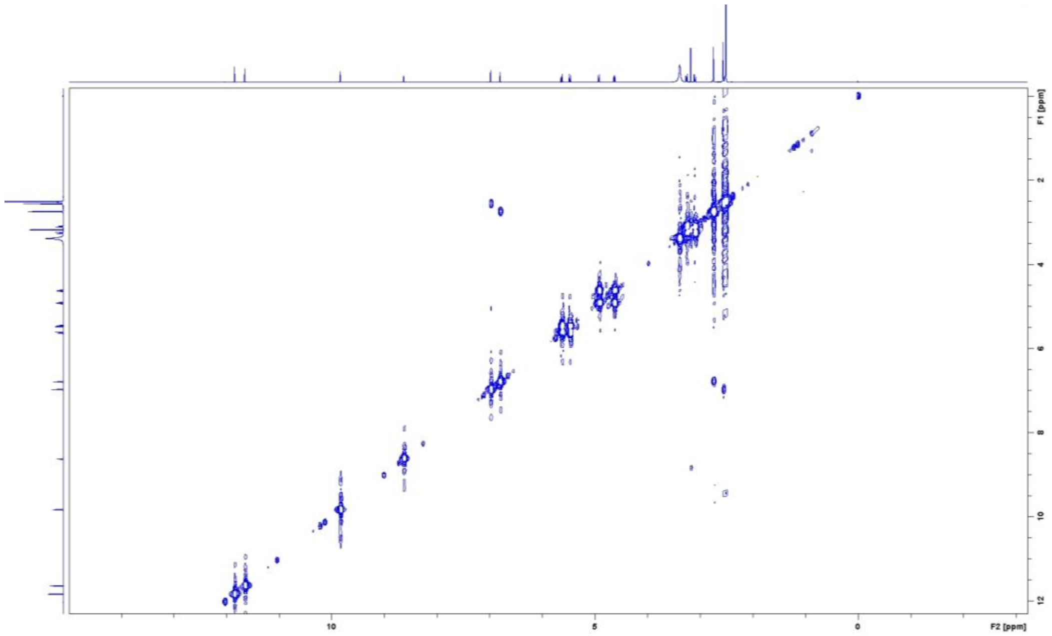

Figure S57. ^1H-^1H COSY spectrum of Compound **16**

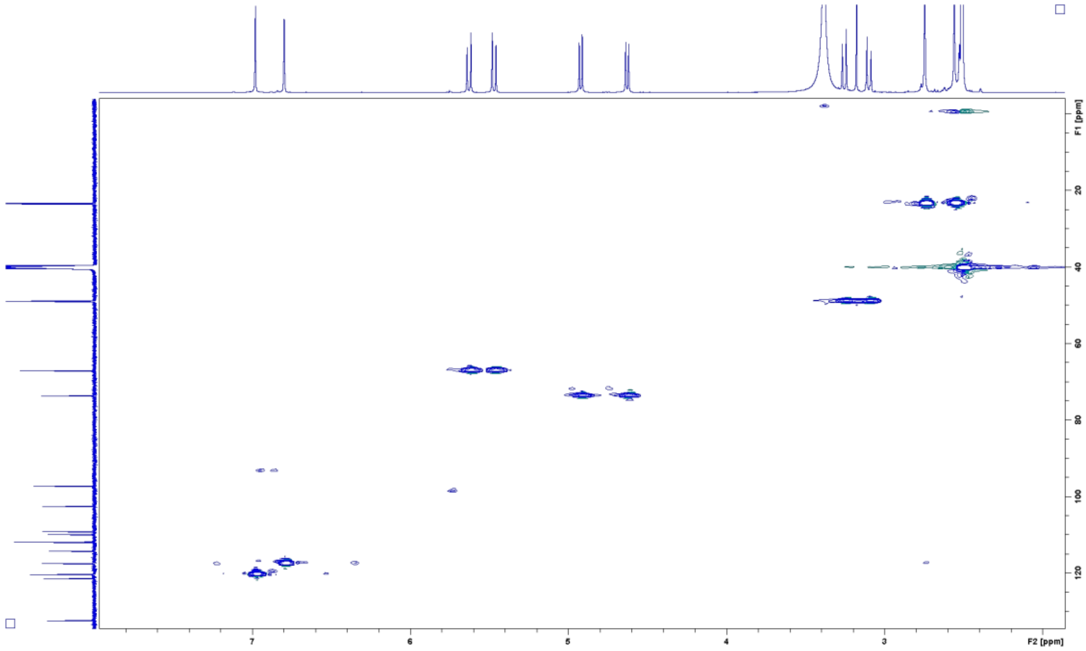

Figure S58. HSQC spectrum of Compound **16**

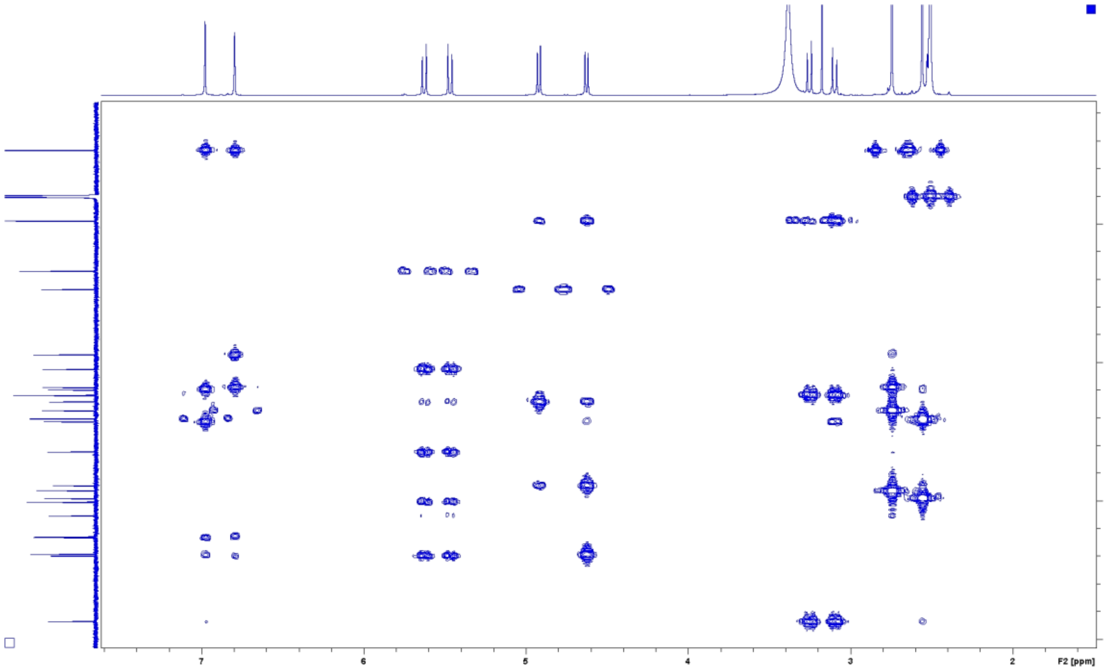

Figure S59. HMBC spectrum of Compound **16**

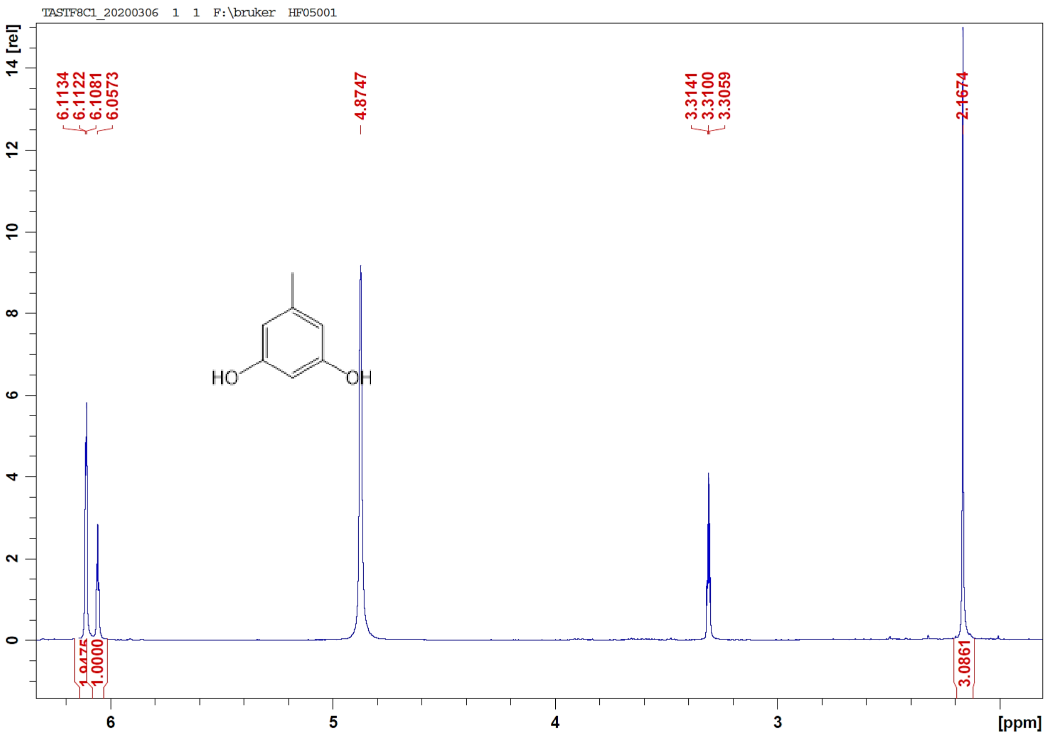

Figure S60. ^{1}H-NMR spectrum of Compound **18**

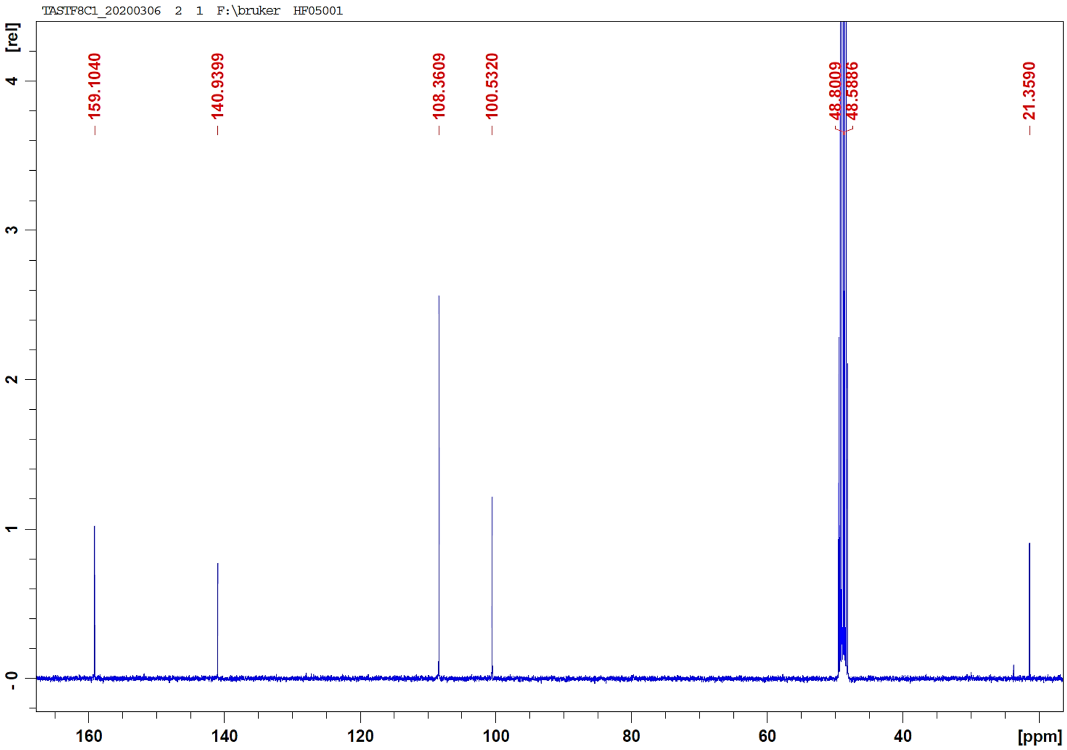

Figure S61. ^{13}C-NMR spectrum of Compound **18**

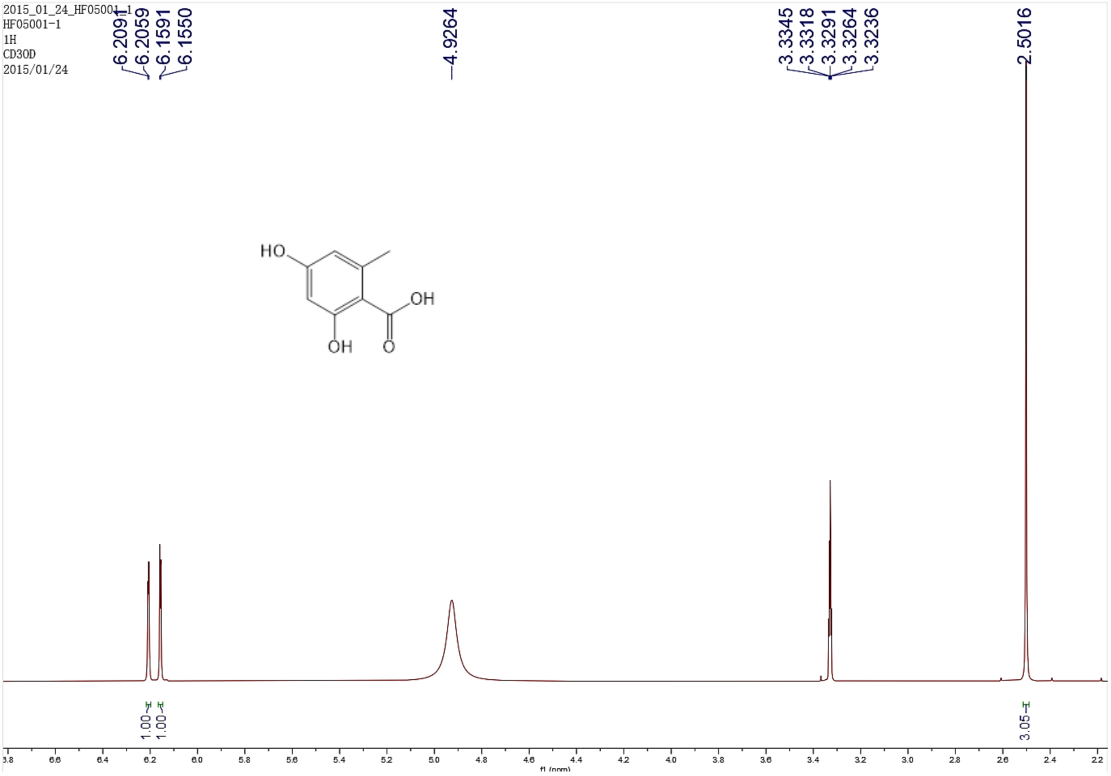

Figure S62. ^{1}H-NMR spectrum of Compound **19**

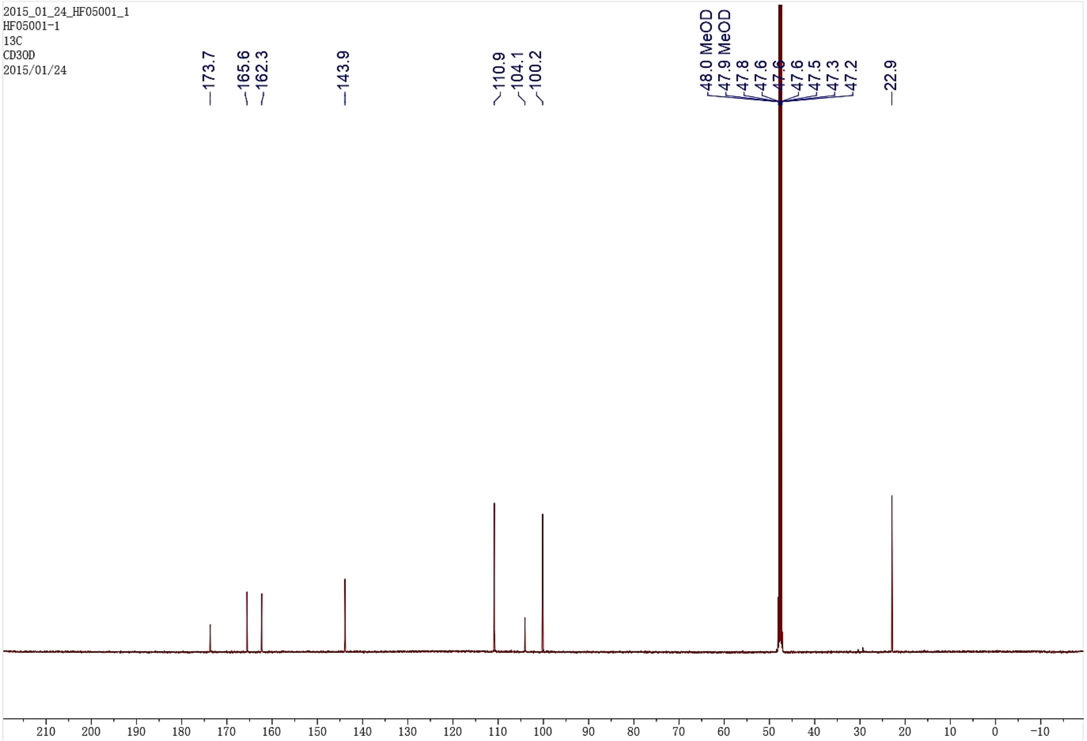

Figure S63. ^{13}C-NMR spectrum of Compound **19**

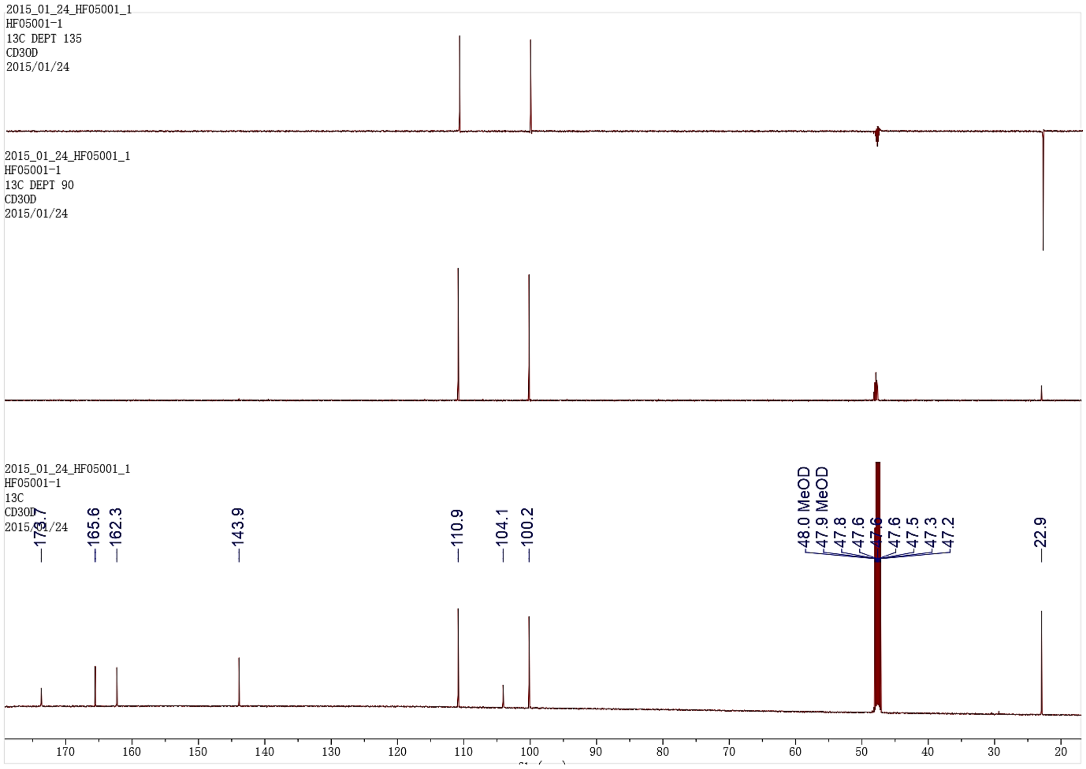

Figure S64. DEPT spectrum of Compound **19**

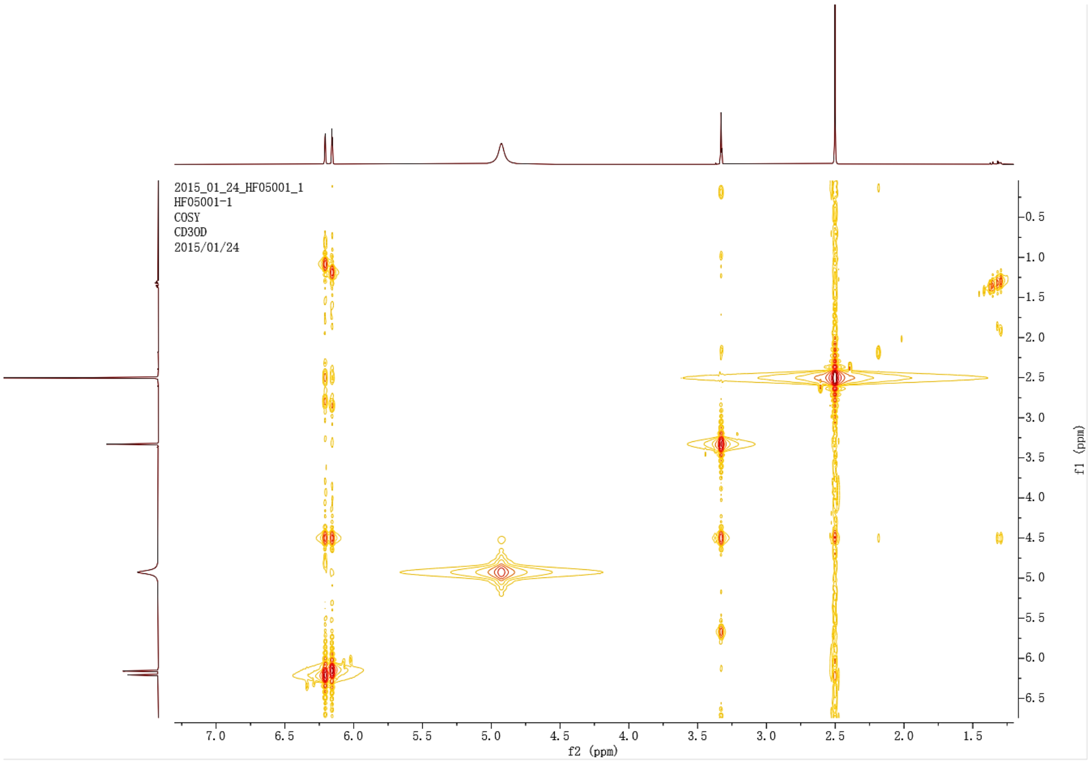

Figure S65. ^{1}H-^{1}H COSY spectrum of Compound **19**

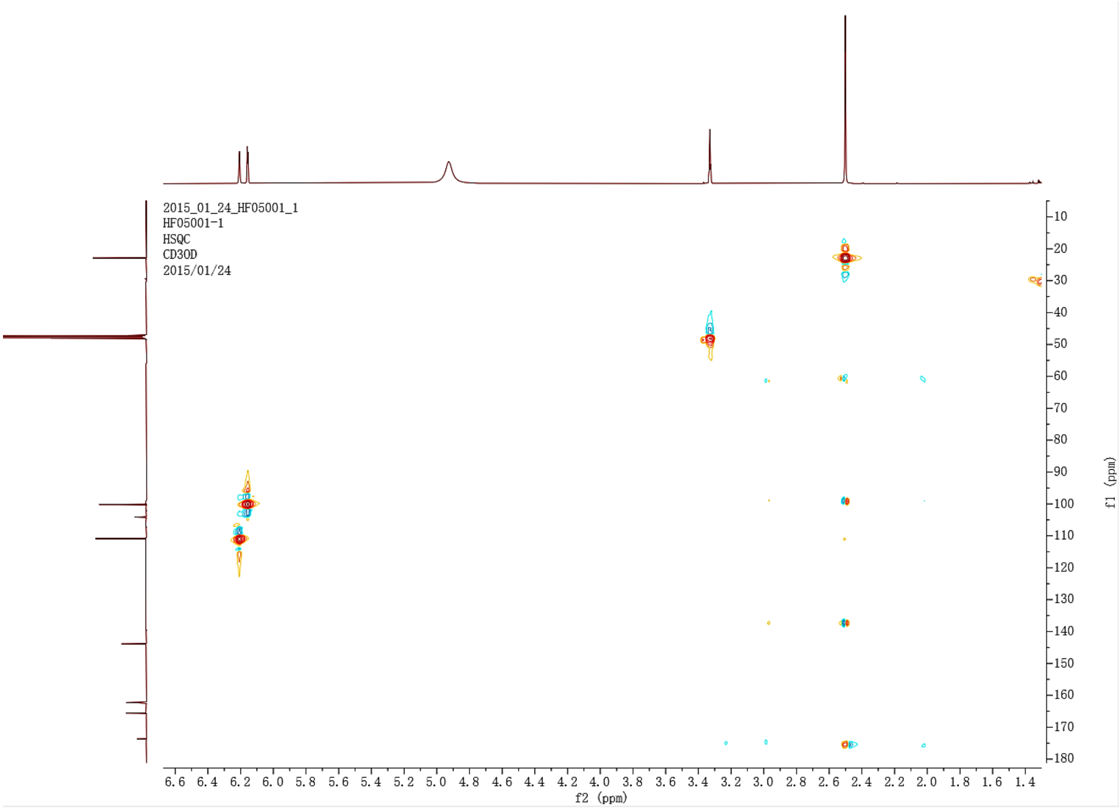

Figure S66. HSQC spectrum of Compound **19**

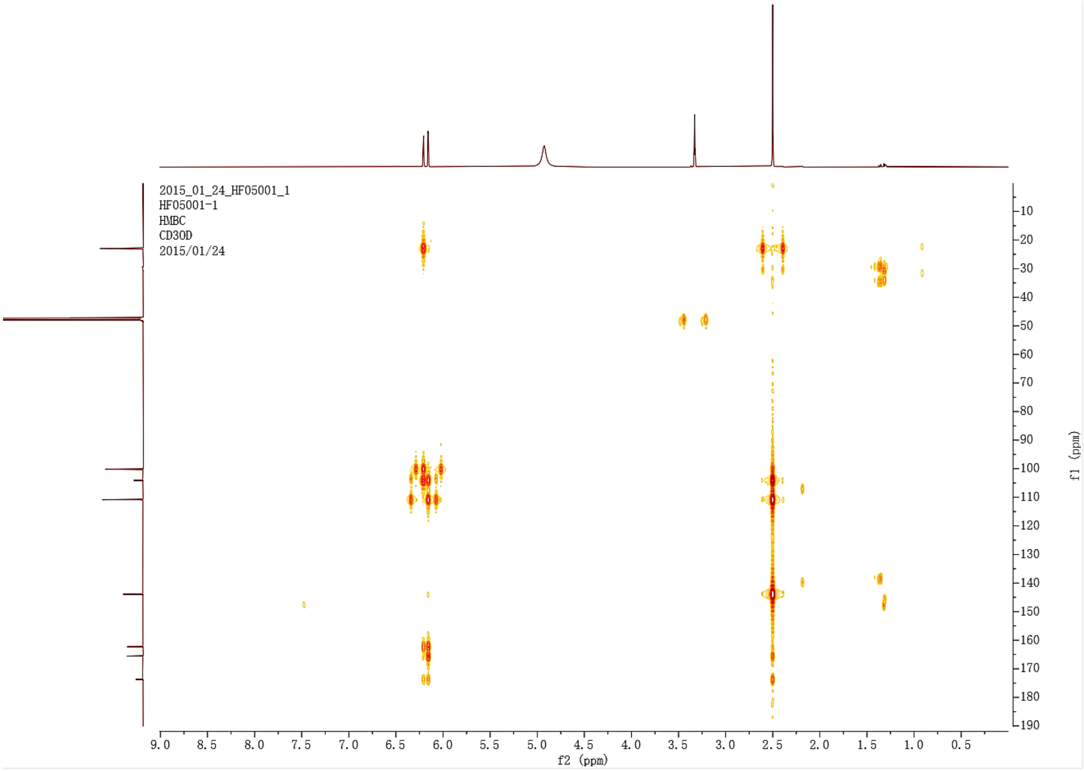

Figure S67. HMBC spectrum of Compound **19**

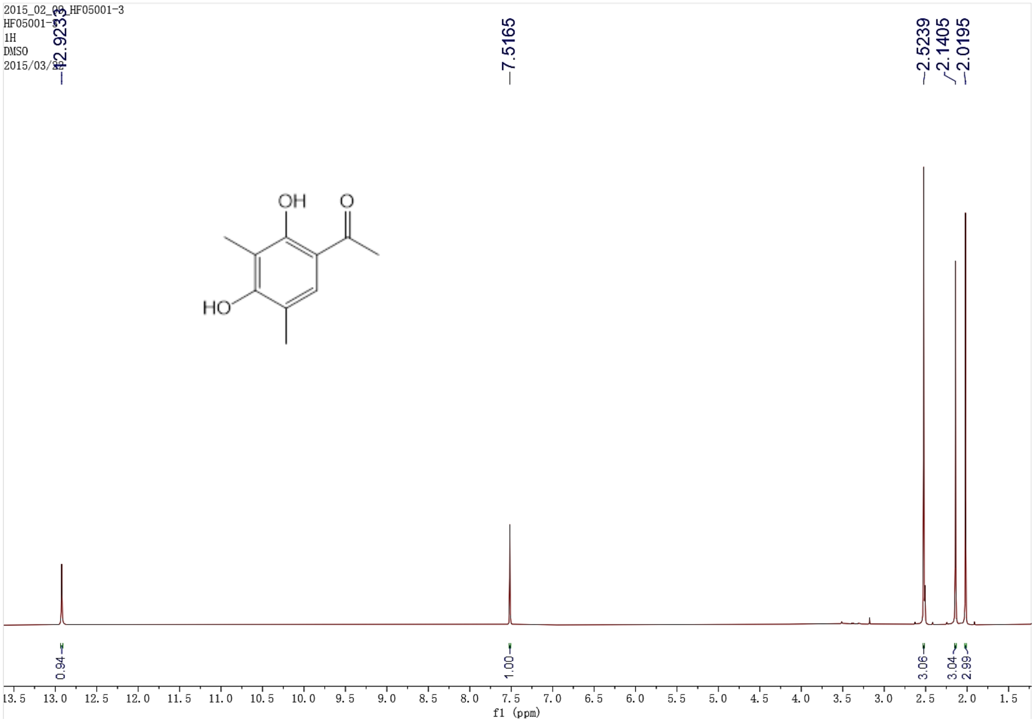

Figure S68. ^{1}H-NMR spectrum of Compound **20**

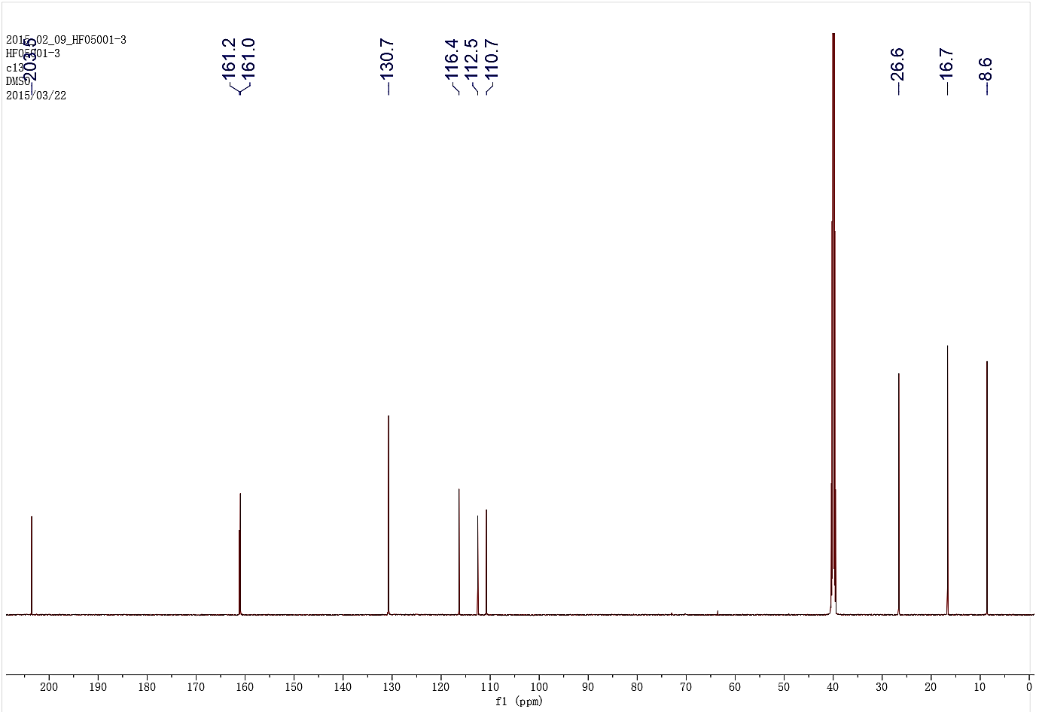

Figure S69. ^{13}C-NMR spectrum of Compound **20**

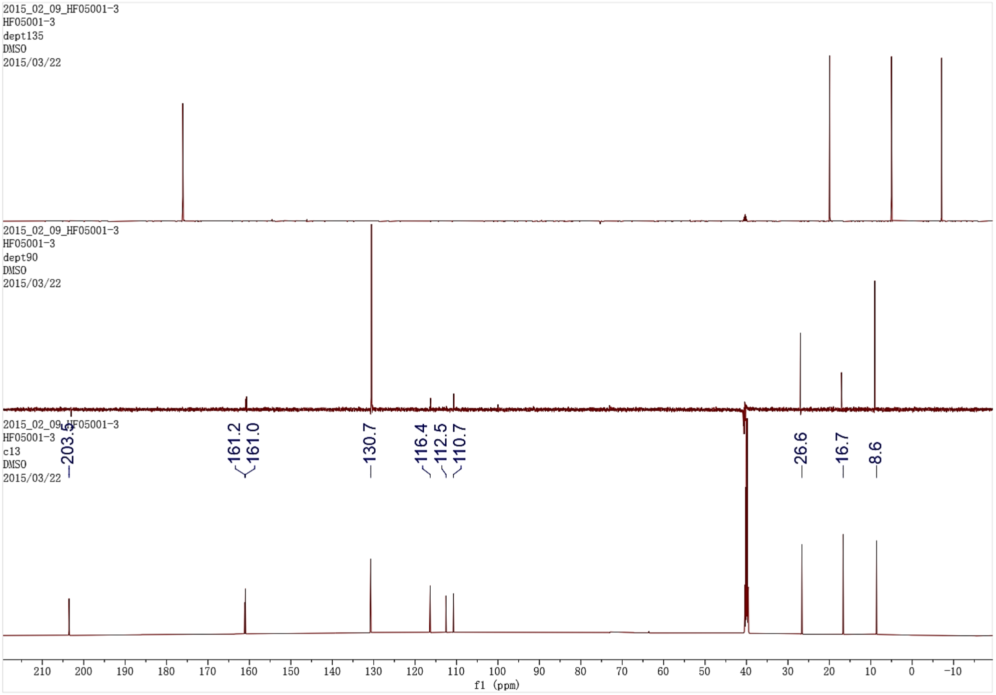

Figure S70. DEPT spectrum of Compound **20**

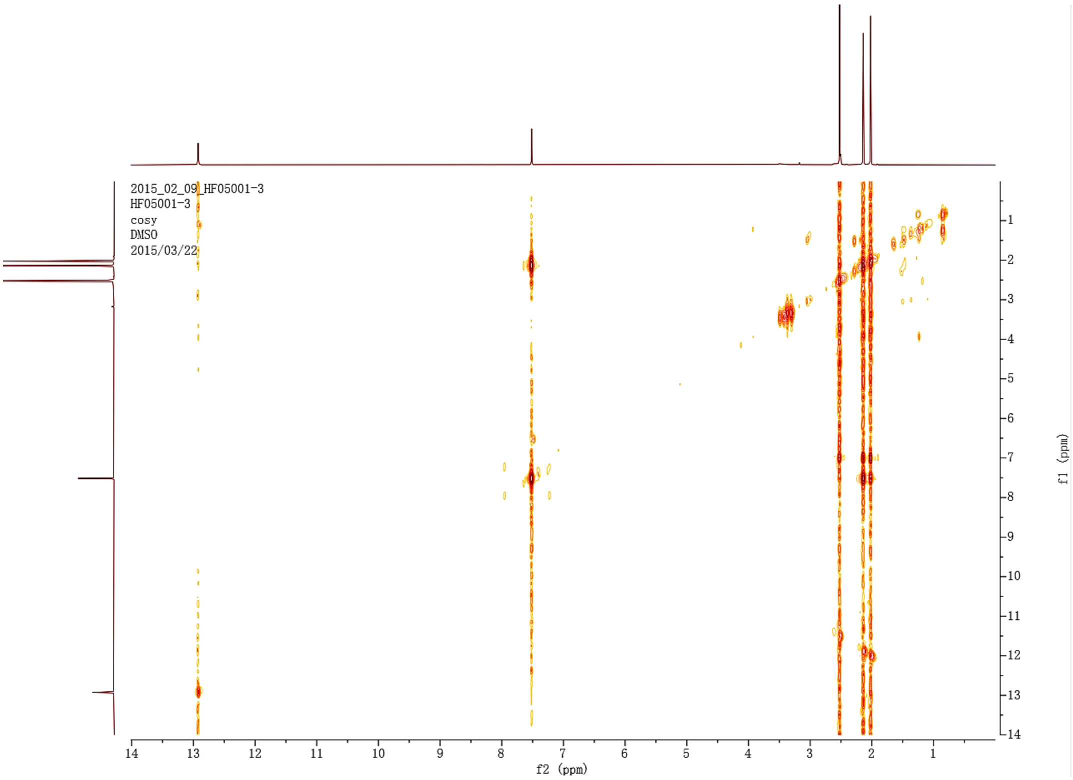

Figure S71. ^{1}H-^{1}H COSY spectrum of Compound **20**

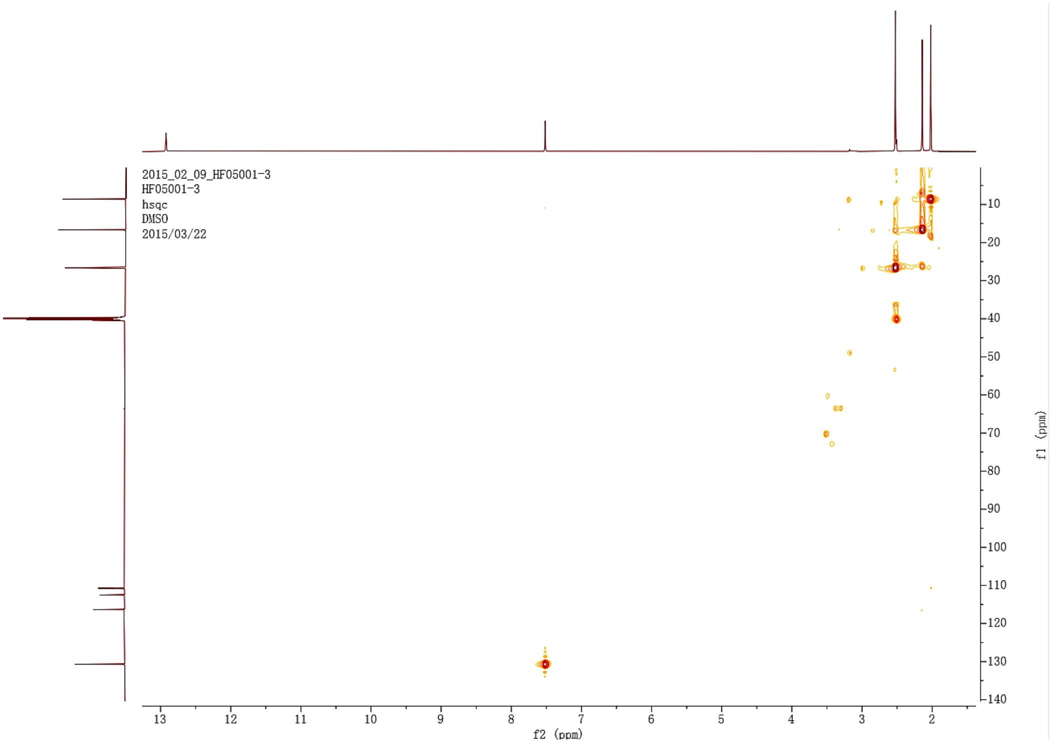

Figure S72. HSQC spectrum of Compound **20**

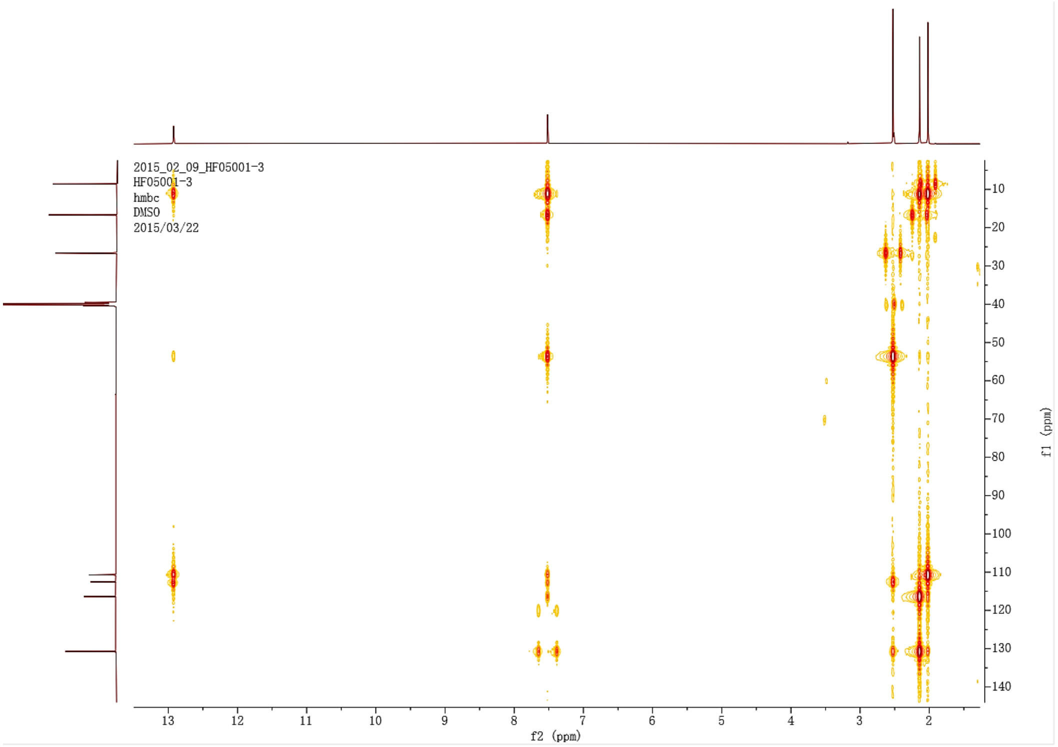

Figure S73. HMBC spectrum of Compound **20**

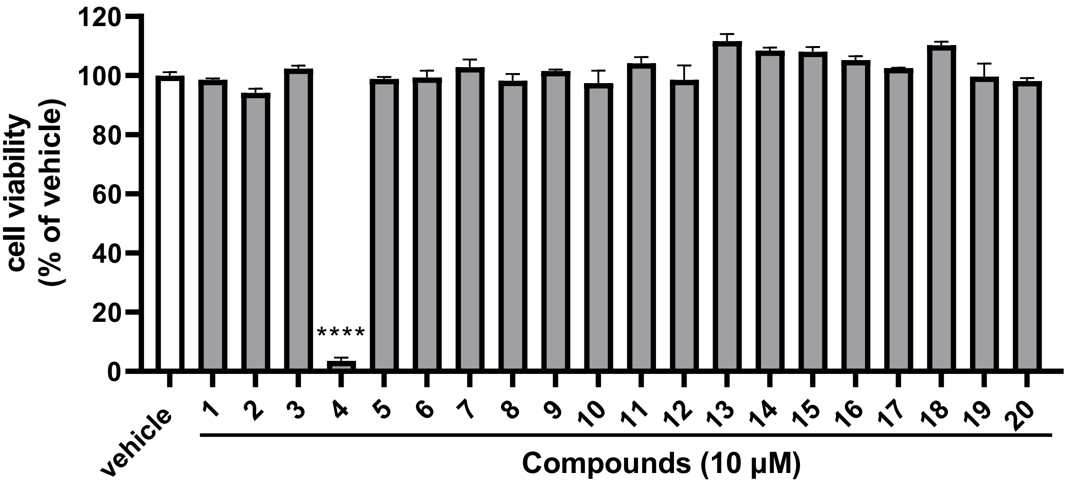

Figure S74: Cytotoxicity effects of compounds 1–20 in RAW264.7 cells. Cell viability was assessed using a CCK-8 assay, with the results presented as a percentage of the vehicle group. **** $p < 0.0001$ versus the vehicle group.

Supplemental Table 1 Detailed DP4+ probability for compound 17. Isomer is 9*S**,12*S**,14*S**,16*S**.

Functional	Solvent?		Basis Set		Type of Data	
mPW1PW91	PCM		6-311G(d)		Unscaled Shifts	
	Isomer 1	Isomer 2	Isomer 3	Isomer 4	Isomer 5	Isomer 6
sDP4+ (H data)	8.47%	0.08%	87.63%	3.83%	–	–
sDP4+ (C data)	34.66%	59.17%	5.92%	0.25%	–	–
sDP4+ (all data)	35.89%	0.55%	63.44%	0.12%	–	–
uDP4+ (H data)	21.01%	1.22%	14.47%	63.29%	–	–
uDP4+ (C data)	91.28%	7.92%	0.48%	0.32%	–	–
uDP4+ (all data)	98.11%	0.50%	0.36%	1.03%	–	–
DP4+ (H data)	10.54%	0.01%	75.10%	14.35%	–	–
DP4+ (C data)	87.03%	12.89%	0.08%	0.00%	–	–
DP4+ (all data)	99.35%	0.01%	0.64%	0.00%	–	–
